# Methanogenic partner influences cell aggregation and signalling of *Syntrophobacterium fumaroxidans*

**DOI:** 10.1007/s00253-023-12955-w

**Published:** 2024-01-13

**Authors:** Anna Doloman, Maaike S Besteman, Mark G Sanders, Diana Z Sousa

**Affiliations:** 1 Laboratory of Microbiology, Wageningen University & Research, Stippeneng 4, 6708 WE Wageningen, The Netherlands; 2 Laboratory of Food Chemistry, Wageningen University, Bornse Weilanden 9, 6708 WG Wageningen, The Netherlands; 3 Centre for Living Technologies, Eindhoven-Wageningen-Utrecht Alliance, Princetonlaan 6, 3584 CB Utrecht, The Netherlands

**Keywords:** *Syntrophobacterium fumaroxidans*, Biofilms, Methanogenic co-cultures, Cell signalling, Anaerobic digestion

## Abstract

**Abstract:**

For several decades, the formation of microbial self-aggregates, known as granules, has been extensively documented in the context of anaerobic digestion. However, current understanding of the underlying microbial-associated mechanisms responsible for this phenomenon remains limited. This study examined morphological and biochemical changes associated with cell aggregation in model co-cultures of the syntrophic propionate oxidizing bacterium *Syntrophobacterium fumaroxidans* and hydrogenotrophic methanogens, *Methanospirillum hungatei* or *Methanobacterium formicicum*. Formerly, we observed that when syntrophs grow for long periods with methanogens, cultures tend to form aggregates visible to the eye. In this study, we maintained syntrophic co-cultures of *S. fumaroxidans* with either *M. hungatei* or *M. formicicum* for a year in a fed-batch growth mode to stimulate aggregation. Millimeter-scale aggregates were observed in both co-cultures within the first 5 months of cultivation. In addition, we detected quorum sensing molecules, specifically N-acyl homoserine lactones, in co-culture supernatants preceding the formation of macro-aggregates (with diameter of more than 20 μm). Comparative transcriptomics revealed higher expression of genes related to signal transduction, polysaccharide secretion and metal transporters in the late-aggregation state co-cultures, compared to the initial ones. This is the first study to report in detail both biochemical and physiological changes associated with the aggregate formation in syntrophic methanogenic co-cultures.

**Keypoints:**

*• Syntrophic co-cultures formed mm-scale aggregates within 5 months of fed-batch cultivation.*

*• N-acyl homoserine lactones were detected during the formation of aggregates.*

*• Aggregated co-cultures exhibited upregulated expression of adhesins- and polysaccharide-associated genes.*

**Graphical abstract:**

**Figure:**
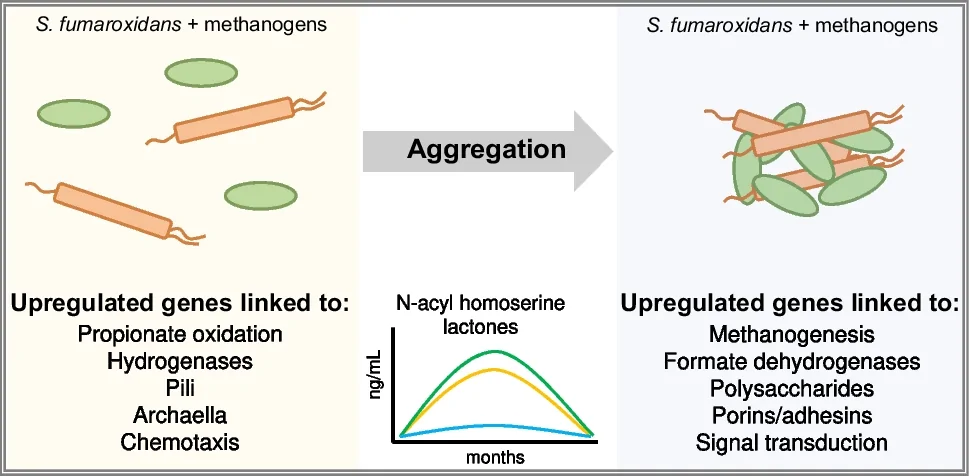

**Supplementary Information:**

The online version contains supplementary material available at 10.1007/s00253-023-12955-w.

## Introduction

High-rate anaerobic wastewater treatment applies granular biofilms to achieve stable and efficient production of biogas (Lettinga et al. 1983). Comprised of tightly interconnected bacteria and archaea of diverse metabolic functionalities, granular anaerobic biofilms (GAB) carry the full anaerobic digestion of organic substrates through hydrolysis, acidogenesis, acetogenesis and methanogenesis. Since the discovery of GAB in the 1980s, most of the studies focused on deciphering the physico-chemical factors affecting the formation and stability of these aggregates (Feldman et al. 2017). To contrast, studies of physiological and molecular principles governing the assembly and organization of microorganisms within the granules have received far less attention (Dang et al. 2022; Chen et al. 2022). This knowledge gap is especially standing out in comparison to the information collected from the organization of related aerobic granular biofilms or activated sludge (Wilén et al. 2018). Above all, most of the knowledge in the field of biofilm research is almost explicitly focused on the surface-attached microbial growth, with vertical or horizontal cell expansion, in either environmental or clinical settings, but not on the suspended aggregate growth, as is the case for GAB (Sauer et al. 2022).

From the studies of aerobic and facultatively anaerobic biofilms, it is known that intracellular signalling (with second messenger molecules, like cyclic-AMP and cyclic-di-GMP) and quorum sensing (QS) play a crucial role in regulating the microbial assembly (Papenfort and Bassler 2016). Through inter- and intracellular communication, microorganisms can synchronize their activity and regulate gene expression within the whole community (Eberl et al. 1996; McNab et al. 2003; Egland et al. 2004; Gantner et al. 2006; Evans et al. 2018; Prentice et al. 2022). Depending on the amount of the QS signalling molecules, such as N-acyl homoserine lactones (AHLs), microorganisms can switch back and forth from an active dispersion growth to a sedentary biofilm-producing mode. Although well-studied QS systems are still restricted to the bacterial communities, a recent report points to the presence of AHL-analogous signalling activity in biofilm-forming archaea (Charlesworth et al. 2020).

In the case of suspended biofilms, like GAB and its aerobic counterpart, the occurrence of QS has been hypothesized based on the detection of AHLs in organic extracts from both sludge and bioreactor media. It has been observed that granulation, considering both aerobic and anaerobic granular systems, is generally correlated with increased levels of AHLs in the sludge phase (although amounts of specific AHLs increased only after the first aggregates are formed) (Chen et al. 2019; Tan et al. 2014; Yan et al. 2021). Furthermore, the addition of synthetic AHLs led to an increased production of extracellular polymeric substances (EPS) by the mixed microbial community within the aerobic and anaerobic sludges, with longer acyl chains (C8-C12) homoserine lactones having a more positive influence on the EPS production in the matured GAB (Lv et al. 2018; Ma et al. 2018; Mit Prohim et al. 2023). It was also shown that microbial communities within aerobic sludge can respond differently to AHLs with varying acyl chain length within a broad concentration range from 10 ng/L to 100 μg/L (Wang et al. 2021). For example, a steady supply of C6-homoserine lactones in high quantity negatively affected the aggregate-forming ability of aerobic sludge and destabilized microbial cell-to-cell adhesions (Shi et al. 2022). Therefore, QS might indeed be relevant for the suspended microbial aggregation, and AHLs can be influencing the biosynthesis and accumulation of EPS in the sludge of mixed microbial communities.

While available knowledge of QS signalling within aerobic and facultatively anaerobic clinically relevant microorganisms can be used as a starting point to learn about the potential QS mechanisms within GAB, identifying the differences in the attached and suspended biofilm-forming mechanisms is critical (Sauer et al. 2022). However, there is a limited number of pure or co-culture studies of obligately anaerobic microorganisms (both bacteria and archaea) forming either attached or suspended biofilms (Krumholz et al. 2015; Cong et al. 2021), which could be useful models to start understanding the molecular mechanisms of GAB formation. Moreover, limited knowledge on QS-mediated biofilm formation in Archaea (Fröls 2013; Orell et al. 2013; Charlesworth et al. 2020) (that can contribute up to 50% by relative abundance to the GAB microbial population) makes molecular understanding of the GAB formation mechanisms even more challenging. Latest studies of QS-associated genes in anaerobic consortia support a correlation for the presence of QS-based interactions between fatty-acid oxidizing bacteria and methanogenic archaea (Yin et al. 2020). However, experimental evidence for these interactions is still lacking.

Here, we present a first attempt to fill in this knowledge gap by looking into the molecular mechanisms behind formation of granular biofilms in the co-cultures of fatty-acid oxidizing bacteria and methanogenic archaea. Co-cultures comprised of propionate-oxidizing *Syntrophobacterium fumaroxidans* (formerly *Syntrophobacter fumaroxidans*) and hydrogenotrophic methanogens, *Methanobacterium formicicum* or *Methanospirillum hungatei,* were maintained for a year in a fed-batch mode to promote aggregate formation. QS signalling molecules, such as N-acyl-homoserine lactones, were identified in the supernatants from the aggregating co-cultures, but were no longer present soon after the mm-scale aggregates matured. Differential gene expression analyses of co-cultures of *S. fumaroxidans* and methanogens in the early- and late-aggregation states pointed to aggregation-associated biochemical changes in the activity of all three microorganisms, especially with regard to the expression of genes in the polysaccharide production and signal transduction systems.

## Materials and methods

### Cultivation of microorganisms

Pure cultures of *Methanospirillum hungatei* strain JF1 (DSM 864^T^), *Methanobacterium formicicum* strain MF (DSM 1535) and *Syntrophobacterium fumaroxidans* (formerly *Syntrophobacter fumaroxidans* strain MPOB, DSM 10017^T^) were obtained from the German Collection of Microorganisms and Cell Culture (DSMZ, Braunschweig, Germany). Microbial cultivations were performed in bicarbonate-buffered mineral salt medium prepared as described previously (Stams et al. 1993). Boiled and N_2_-flushed medium (46 ml) was dispensed into 117 ml serum vials, which were sealed with butyl rubber septa and crimped with aluminium caps. Vials’ headspace was flushed with 80/20 (v/v) H_2_/CO_2_ (for growing pure cultures of methanogens) or 80/20 (v/v) N_2_/CO_2_ (for syntrophic co-cultures and pure *S. fumaroxidans* MPOB cultures) and finally pressurized to 1.5 bar. Medium was autoclaved at 120°C for 20 min. Before inoculation medium was supplemented with filter-sterilized vitamins solution (Stams et al. 1993) and reduced with Na_2_S.xH_2_O (*x*=9–11 mol) (added to a final concentration of ~ 1mM). Pure cultures of *S. fumaroxidans* were grown on 20 mM sodium propionate as an electron donor and 15 mM sodium sulfate as an electron acceptor. Co-cultures of *S. fumaroxidans* and methanogens were grown on 20 mM sodium propionate alone.

Syntrophic co-cultures were constructed by mixing 10% v/v inoculum of the pre-grown pure culture of *S. fumaroxidans* and 10% (v/v) of one of the pre-grown pure cultures of methanogens. All co-cultures were prepared in triplicates. Pure cultures of methanogens were incubated on a shaker platform (180 rpm), while co-cultures and pure cultures of *S. fumaroxidans* were not shaken during incubation. Cultivations of pure cultures were done by transferring 10% (v/v) of an actively growing culture into a bottle with fresh medium. All cultures grew at 37°C in the dark.

### Aggregate formation experiment

Aggregate formation experiments were performed in 250-mL serum bottles with 100 mL mineral medium. Bottles were inoculated with the 10% (v/v) of exponentially growing cultures of *S. fumaroxidans* and *M. formicicum* or *M. hungatei* (co-cultures Sf-Mf and Sf-Mh) in triplicate. Co-cultures were maintained in a fed-batch mode, by routinely exchanging 50% of the medium with fresh medium containing 40 mM of propionate (to achieve 20 mM propionate in the cultivation media) (Figure S1). Media replacement was done when propionate concentration in the medium reached about 3–4 mM. Since co-cultures were grown non-shaking, each medium exchange was done as carefully as possible removing the top liquid part of the serum bottle with a syringe, while trying not to disturb any precipitated/aggregated cells at the bottom of the bottle. Each medium exchange denoted a new “Cycle” in the cultivation, allowing to track the number of nearly complete growth cycles of the co-cultures. When pure cultures of *S. fumaroxidans* and each of the methanogens were first mixed in the same bottle (10% v/v of each co-culture member), “Cycle 0” commenced. Transfer of 10% of the grown together co-cultures from Cycle 0 to the new bottle with fresh medium and 20 mM propionate commenced “Cycle 1” (also referred to as “early-aggregation state co-cultures”). The first 50% medium exchange in the Cycle 1 bottle at the 80–90% of propionate consumption denotes the start of “Cycle 2.” The scheme of exchanging 50% of media and numbering of progressions is then maintained. Cultures purity was routinely checked with light microscopy.

### Extraction of RNA

Total RNA extractions were done from the cells of triplicates of the early- (Cycle 1) and late-aggregation state (Cycles-20/23) co-cultures and pure cultures of *S. fumaroxidans* and two methanogens in the mid-exponential growth (~ 10 mM propionate or 70% (v/v) H_2_ consumed). Cell pellets were harvested from 110 mL of each culture by centrifugation at 10,000g at 4 °C (Sorvall Legend XTR, Thermo Fisher, Waltham, MA) under sterile conditions, washed in the sterile cold TE buffer, snap-frozen in liquid nitrogen and stored at −70 °C. Lysis and protein precipitation was performed using the solutions and enzymes from MasterPure™ Gram Positive DNA Purification Kit (Bioresearch Technologies, UK). Briefly, lysozyme (1 μL) incubation was done at 37°C for 20 min, followed by addition of 4 uL of β-mercaptoethanol, sonication using Bendelin SONOPULS HD 3200 ultrasonic homogenizer (6 cycles of 20 s pulse 30 s pause) and proteinase K incubation at 60°C for 15 min. Protein precipitation was performed according to the kit specifications. Automated RNA purification was performed using Maxwell® 16 MDx instrument and LEV simplyRNA Purification Kit (Promega, Madison, WI). RNA was sequenced at Novogene (Novogene, UK) on the NovaSeq6000 (Illumina, San Diego, CA), yielding 150 bp paired end reads.

### Transcriptomics

Obtained transcriptome reads were trimmed by bbduk.sh of BBmap (v38.84) (ktrim=r, k=23, mink=7, hdist=1, tpe, tbo, qtrim=rl, trimq=30, ftm=5, maq=20, minlen=50, hdist=1), followed by fastQC quality check (v0.11.9). Three reference genomes were annotated with KEGG (Kanehisa et al. 2016), InterPro scan (Paysan-Lafosse et al. 2023) and Prokka v1.14.6 (Seemann 2014): *S. fumaroxidans* (RefSeq GCF_000014965.1), *M. hungatei* (RefSeq GCF_000013445.1) and *M. formicicum* (RefSeq GCF_029848115.1). Sequences of hydrogenases were additionally checked with conserved domain search (Lu et al. 2020) and HydDB (Søndergaard et al. 2016) for the specific catalytic subunits. All transcripts were mapped against all three reference genomes using bbsplit.sh and mapped genes were counted using samtools view (version 1.10) (-SF 260, cut -f 3). Mapped counts were further analysed in Rstudio (v4.0.2), separately for each organism. The Rmd script used for data analysis is supplied (Supplementary file 1). To determine gene expression ranking within each of the two conditions, counts were normalized to Transcripts Per Million (TPM). The Bioconductor package DESeq2 v1.30.16 (Love et al. 2014) was used for additional normalization to allow differential expression analysis between the samples. To determine significant differential expression, we considered a fold-change ≥ 1.5, *p*-value adjusted by the Benjamini and Hochberg method to ≤ 0.05 (Benjamini and Hochberg 1995). Data visualization was performed in R, using DESeq2 normalized counts. Raw reads were deposited to the European Nucleotide Archive with the study accession number ERP148018.

### Scanning electron microscopy (SEM)

Stationary phase grown aggregated co-cultures from Cycles 30/34 were subject to scanning electron microscopy. Centrifugation of the samples was avoided during sampling to prevent unnatural clumping of microbial cells and thus, interference with detection of any aggregates. Aliquots of culture samples (or single aggregates) were directly mounted on coverslips coated with Poly-L-Lysine (Corning BioCoat, Corning Life Sciences, Tewksbury, MA) and fixed with 3% (v/v) glutaraldehyde and 1% (v/v) OsO_4_ for 1 h at room temperature. Next, samples were dehydrated in graded ethanol solutions in water (10, 30, 50, 70, 80, 90, 96, 100%) for 10 min each and critical point dried with liquid carbon dioxide using an automated critical point dryer Leica EM CPD300 (Leica, Wetzlar, Germany). Cells were studied with FEI Magellan 400 scanning electron microscope.

### Fluorescent microscopy

Syto 16 (Invitrogen, Waltham, MA) was applied to stain DNA of all the cells in the samples. Aliquots of 200 μL were sampled from stationary phase grown aggregated co-cultures and washed with 1.5 mL PBS buffer (1.8 g L^−1^ Na_2_HPO_4_, 0.223 g L^−1^ NaH_2_PO_4_, 8.5 g L^−1^ NaCl, pH 7.2) by centrifuging for 3 min at 4000 g at 10°C. The staining was done on the pellets resuspended 1 mL PBS, by adding 40 μL of 96% ethanol and 1 μL of Syto16 (1 mM solution in DMSO). Samples were incubated for 3 h covered with aluminium foil, in the dark, at room temperature. Stained samples were visualized under the fluorescent microscope Nikon Eclipse Ti2 with 100× objective using green fluorescence filter (excitation 465/495 nm, emission 515/555 nm) to select for Syto 16 fluorescence and blue fluorescence filter (excitation 340/380 nm, emission 435/485 nm) to select for the autofluorescence of cofactor F_420_ of the methanogenic cells. Two fluorescent signals were overlayed in the final image processed in NIS Elements AR (version 5.21.03 64-bit).

### Analytical methods

Gaseous compounds (H_2_, CH_4_) were analysed by gas chromatography on a CompactGC^4.0^ (Interscience, Breda, Netherlands) equipped with a thermal conductivity detector. Argon gas was used as a carrier gas at a flow rate of 1 ml min^−1^. Gas sample (0.2 ml) was injected onto a Carboxen 1010 column (3 m × 0.32 mm) followed by a Molsieve 5A column (30 m × 0.32 mm). The temperatures in the injector, column and detector were 100°C, 140°C and 110°C, respectively. The limit for H_2_ detection was 0.01% v/v (0.006mM). Organic acids (formate, acetate, propionate) were quantified using a Shimadzu HPLC (Kyoto, Japan), equipped with a Shodex column (SH-1011), and UV/RID detectors. A flow rate of 1 ml min^−1^ was used with sulphuric acid (0.01 N) as mobile phase and column temperature set at 45 °C. The limits of quantification were 0.2–0.5 mM for all three organic acids.

### Extraction of N-acyl homoserine lactones (AHLs)

Cultures’ supernatants were collected at the late exponential growth phase at fed-batch Cycles 1 to 33 by centrifugation at 10,000 × g for 20 min at 4°C (Sorvall Legend XTR, Thermo Fisher, Waltham, MA) in sterile 50 mL (Greiner, Germany). AHLs were extracted twice from the supernatants by adding equal volumes of ethyl acetate-acetone mixture (4:1, v/v) to a conical flask containing the supernatant, which was then sealed with parafilm and shaken at room temperature at 180 rpm for 1 h. After that, supernatant-extractant mixture was transferred to a separation funnel, and the upper organic phase was collected. Bottom aqueous phase was subject to a second extraction. Pooled extracts from two extractions were dehydrated by passing through an anhydrous NaSO_4_-packed syringe column. Flowthroughs were filtered through a 0.45-μm hydrophilic PTFE syringe filter (BGB, China) and dried on the SpeedVac (Eppendorf, Germany) with high-vacuum settings at 30°C. Dried extracts were stored at −20°C.

### AHL separation with UHPLC

Dried extracts were resuspended in 3mL of HPLC-grade acetonitrile (Sigma Aldrich, St. Louis, MO), and 2 μL was injected on a Vanquish Horizon UHPLC system (Thermo Scientific, San Jose, CA). The autosampler temperature was set at 10 °C. ACN was used as the strong needle wash before and after injection. For separation, an Acquity BEH-C18 column (150 × 2.1 mm, Waters, Milford, MA) with BEH-C18 VanGuard guard column (5 × 2.1 mm, Waters, Milford, MA) were used. Gradient elution was done using mobile phase A (premix 0.1% formic acid in UHPLC-grade water; Biosolve, Valkenswaard, The Netherlands) and mobile phase B (premix 0.1% formic acid in acetonitrile; Biosolve, Valkenswaard, The Netherlands). The flow rate of mobile phases was 0.4 mL × min^−1^. The gradient settings were: 0.00–1.10 min isocratic on 5% B; 1.10–24.88 min linear gradient from 5%B to 70 %B; 24.88–25.97 min linear gradient from 70 % B to 5 % B; and 25.97–31.50 min isocratic on 5% B. The column temperature was set at 45°C and the post-column cooler at 40°C.

### AHL detection with mass spectrometry

AHLs were analysed on a Thermo Q Exactive Focus hybrid quadrupole-orbitrap mass spectrometer (Thermo Scientific, San Jose, CA) equipped with a heated ESI probe (ESI-FTMS). Before analysis, the orbitrap was calibrated in positive ionization mode using Tune 2.11 (Thermo Scientific, San Jose, CA) by injection of Pierce positive ion calibration solutions (Thermo Scientific, San Jose, CA). The parameters of the positive ion mode were as follows: Nitrogen was used as a sheath gas (50 arbitrary units), auxiliary gas (13 arbitrary units) and sweep gas (1 arbitrary unit). FullMS data in the mass in the mass range 170–450 (m/z) was recorded using 70.000 FWHM mass resolution. The source voltages were set at 3.5 kV, the S-lens RF level was set at 50%, the capillary temperature at 263 °C and the auxiliary gas heater temperature at 425 °C. Data acquisition and processing were performed using Xcalibur version 4.3 (Thermo Scientific, San Jose, CA).

Standards of seven AHLs (Table S1), namely N-Butyryl-DL-homoserine lactone (C4-HSL), N-Hexanoyl-L-homoserine lactone (C6-HSL), N-(Ketocaproyl)-d,l-homoserine lactone (3-oxo-C6-HSL), N-Heptanoyl-DL-homoserine lactone (C7-HSL), N-Octanoyl-L-homoserine lactone (C8-HSL), N-(3-Oxodecanoyl)-L-homoserine lactone (3-oxo-C10-HSL) and N-Dodecanoyl-L-homoserine lactone (C12-HSL), were purchased from Sigma-Aldrich (St. Louis, MO) and dissolved in HPLC-grade acetonitrile to prepare standard curves for the UHPLC-MS/MS. Serial dilutions were prepared for each standard ranging from 0.5 to 50 ng/mL. Standards of AHLs were tested twice during each UHPLC-MS/MS sequence: at the beginning and at the end of the sequence. Values from the peak areas averaged from the two injections of the same standard were used to build a standard curve. Standard curves with *R*^2^=0.99 of varying carboxyl chain length AHLs were prepared freshly before each analysis of the culture extracts on the UHPLC-MS/MS. The limit of detection (LOD) for tested AHLs was determined from the calibration curves by applying root mean square error (RMSE) (Andini et al. 2020). The limit of quantification (LOQ) was calculated as 3 times the value of LOD. The LOD varied slightly between MS/MS runs and ranged within 0.2–0.5 ng/mL for different molecules in selective ion mode (SIM) (Table S2, Figure S2). Mass filter used for quantification in SIM mode was set to 5ppm. For validation of the AHL extraction, recovery and identification procedures, the sterile bicarbonate-buffered mineral salt medium was spiked with synthetic C4-HSL (5 ng/μL) and processed as a separate sample alongside the co-culture supernatants. Recovered amount of the C4-HSL was then calculated from the MS/MS chromatograms by comparing the areas of the peaks to the areas in the standard curves of the C4-HSL from the same UHPLC-MS/MS sequence.

## Results

### Physiological and morphological analysis of early- and late-aggregation state co-cultures

**Fig. 1:**
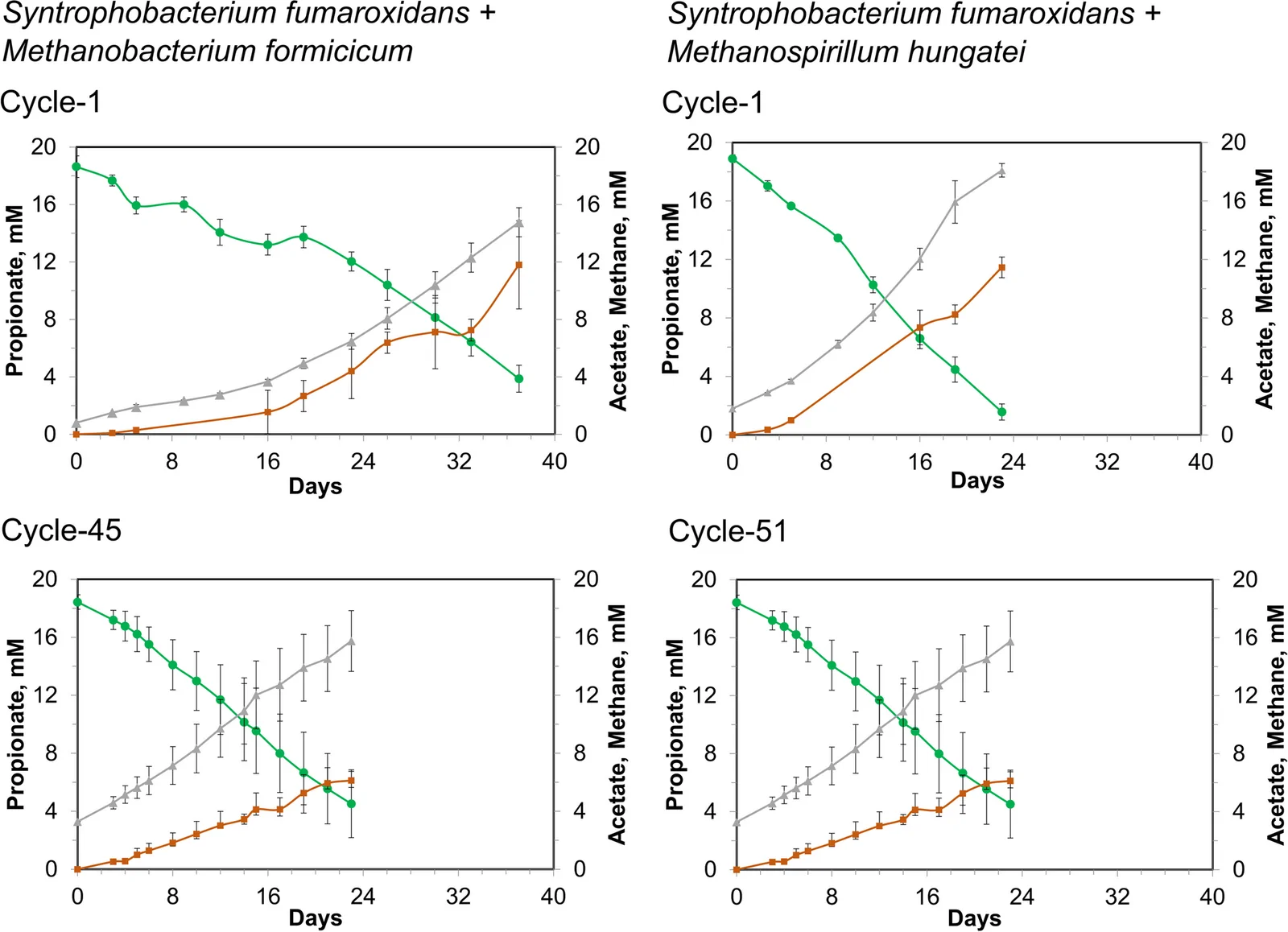
Propionate (green), methane (orange) and acetate (grey) measured in the co-cultures of *S. fumaroxidans* and *M. formicicum* (left panel) or *M. hungatei* (right panel). Cycle 1 corresponds to the early-aggregation state co-cultures, while Cycles 45 and 51 correspond to the late-aggregation state

In this study, we successfully achieved aggregation of two syntrophic propionate-oxidizing microbial co-cultures comprised of (1) *Syntrophobacterium fumaroxidans* with *Methanobacterium formicicum* (noted Sf-Mf) and (2) *Syntrophobacterium fumaroxidans* with *Methanospirillum hungatei* (noted Sf-Mh). In both syntrophic co-cultures, *S. fumaroxidans* performed the oxidation of propionate to hydrogen and acetate, while methanogens converted hydrogen and CO_2_ into methane (Fig. 1). Both methanogens, *M. formicicum* and *M. hungatei*, are also known to be able to use formate as an electron donor, in addition to hydrogen (Schauer et al. 1982). While acetate and methane were produced in all the co-cultures (Fig. 1), neither formate nor hydrogen was above the instrumental sensitivity level of 0.5 mM (formate) or 0.006 mM (hydrogen) at any point of co-culture growth. The consistent production of methane, without significant lag phase, demonstrates the efficient syntrophic relationship in the co-cultures studied here.

**Fig. 2:**
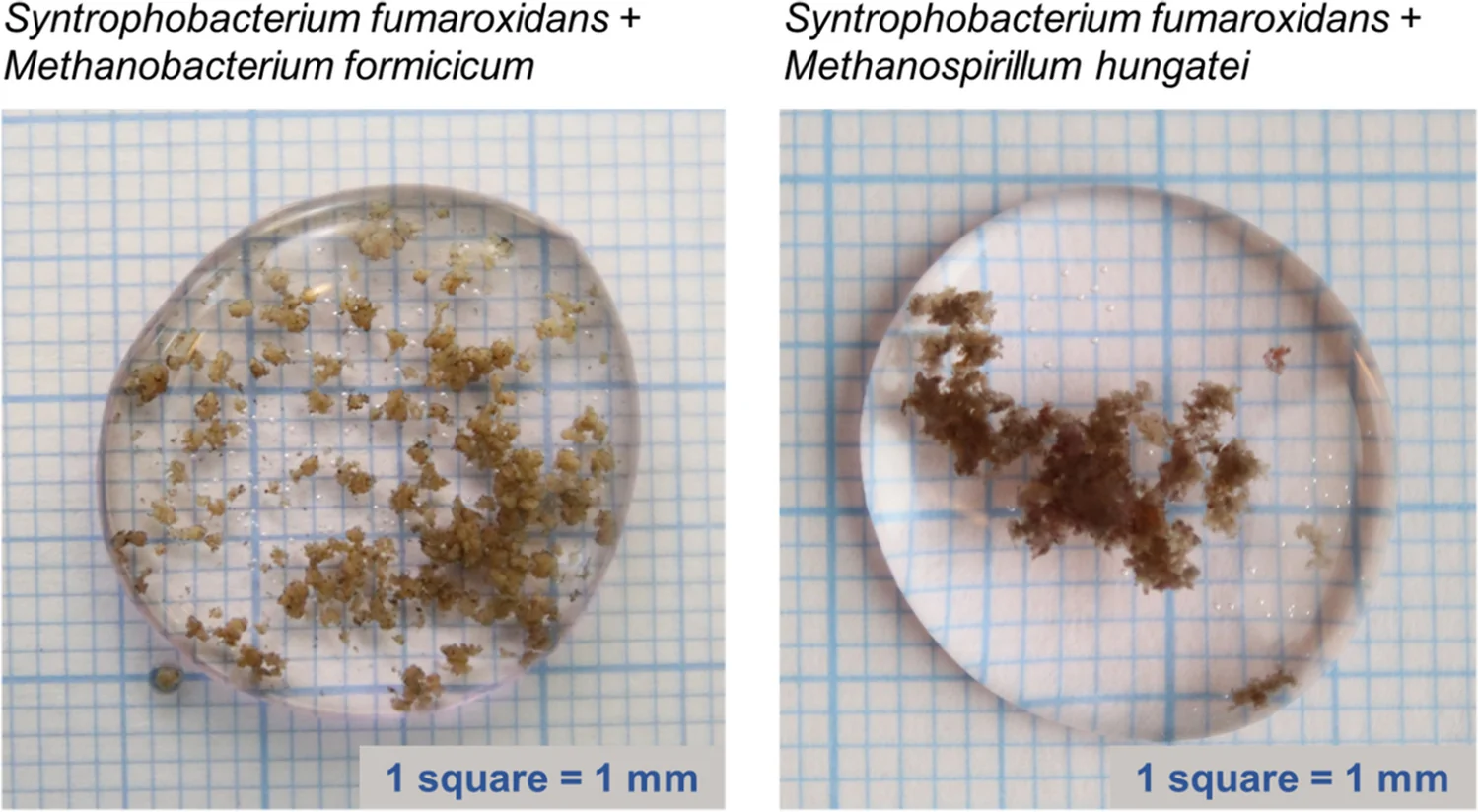
Photographs (non-magnified) of the aggregates formed after 1 year of continuous growth of *S. fumaroxidans* co-cultures with *M. formicicum* (Cycle 45) and with *M. hungatei* (Cycle 51)

Pure cultures of *S. fumaroxidans* and *M. hungatei* did not aggregate at any stage of their growth, while pure cultures of *M. formicicum* self-aggregated in the early stationary phase (Figure S3). Meanwhile, Sf-Mf co-cultures formed small aggregates (less than 10 μm in diameter), which were visible under the 100× magnification in the light microscope, already at Cycle 0. However, they were not visible to the naked eye until Cycle 8 (Figure S3). Sf-Mh co-cultures produced visible aggregates later, at Cycle 13. Overall, it took 5 months for both co-cultures to produce mm-scale aggregates visible to the naked eye (Fig. 2). Using the same volumetric loading of the inoculum from early- and late-aggregation state co-cultures (Figure S1), we saw that both Sf-Mf and Sf-Mh co-cultures had increased propionate oxidation rates in Cycle 45/51, compared to the co-cultures from Cycle 1: from 0.55 ± 0.03 to 0.70 ± 0.09 (mM_propionate_.day^−1^) for Sf-Mf and from 0.79 to 1.03 ± 0.12 (mM_propionate_.day^−1^) for Sf-Mh (Fig. 1).

**Fig. 3:**
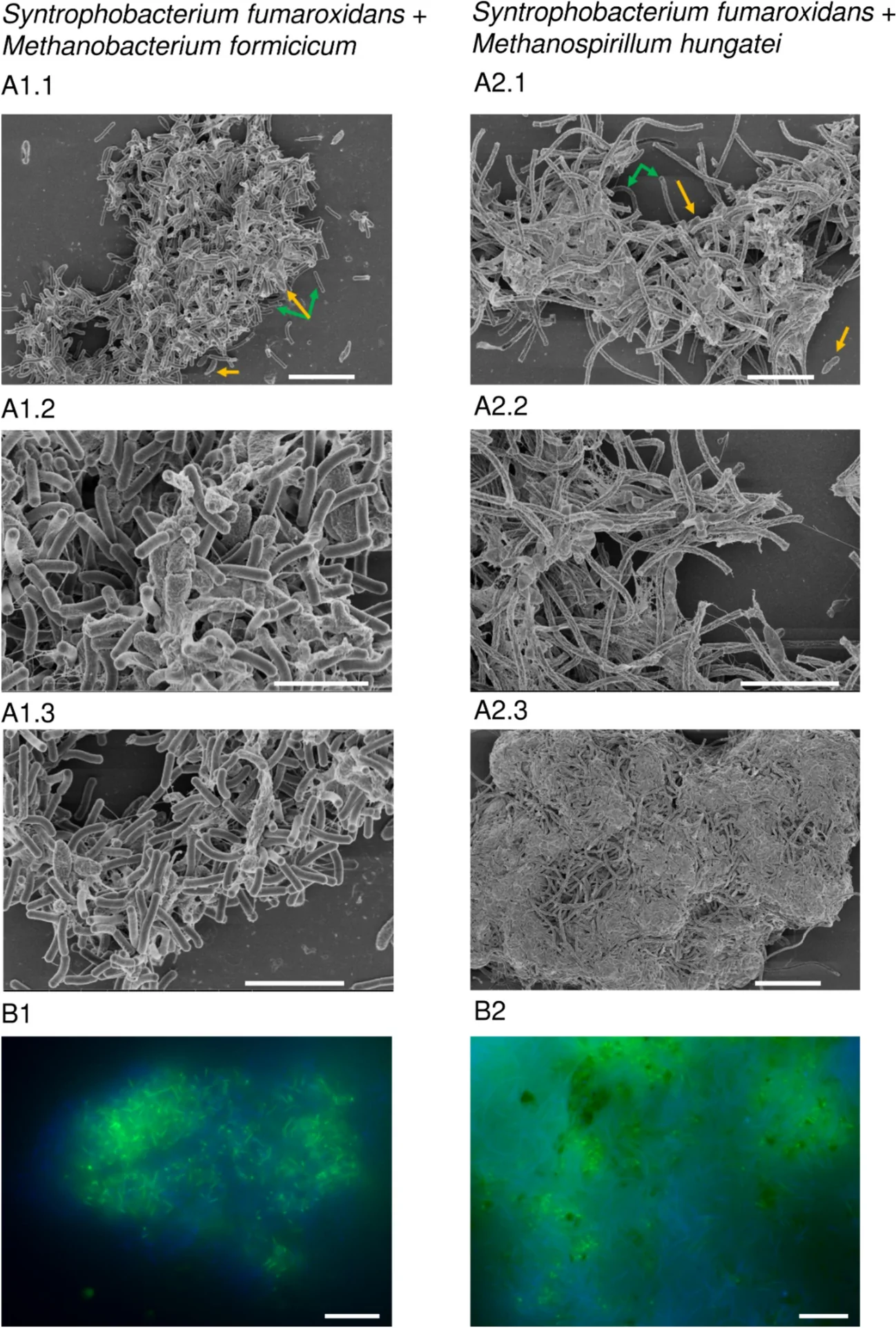
**A** Scanning electron microscopy imaging of *S. fumaroxidans* (yellow arrow) aggregates with *M. formicicum* (1, sampled at Cycle 30) or *M. hungatei* (2, sampled at Cycle 34) (green arrow). **B** Overlayed epifluorescent imaging of the syntrophic aggregates with *M. formicicum* (1) or *M. hungatei* (2). Green florescence comes from the Syto 16 staining, while blue fluorescence comes from the autofluorescence of cofactor F_420_ (see “Materials and methods” for details). Images A1.1, A2.1-2 have a scale bar of 5 μm; A1.3 has a 3 μm scale bar; A2.3 and B1-2 have a 10-μm scale bar

Both aggregated Sf-Mf and Sf-Mh co-cultures were examined using scanning electron microscopy (SEM) and fluorescent microscopy at the Cycle 30/34 (Fig. 3). Overlaying autofluorescence of the methanogenic cofactor F_420_ with the fluorescence signal from general DNA dye Syto16 allowed to pinpoint location of the two microbial species in co-cultures. This revealed that the two species are intertwined or closely associated within the clusters.

### Identification and characterization of N-acyl-homoserine lactones in the syntrophic co-cultures

Supernatants from the methanogenic co-cultures, pure cultures of *S. fumaroxidans* and pure cultures of the two methanogens were collected at different aggregate formation stages to check for the presence of extracellularly secreted N-acyl-homoserine lactones (AHLs) (Materials and Methods, Figure S4, S5). All cultures were sampled at the late exponential growth stages. The analysis of the supernatants from the pure cultures and co-cultures at different aggregation stages revealed the presence of varying quantities of four AHLs (Table 1). C7-HSL, C8-HSL and C12-HSL were found in most extracts, both in the pure cultures of *M. formicicum* and *M. hungatei*, and their respective co-cultures with the syntroph. C12-HSL was present in the highest quantities in some samples (Cycle 9, Sf-Mh co-cultures), although biological replicates of the extracts demonstrated a high variability in the amounts of the molecule (Table 1). 3-Oxo-C6-HSL was found in most of the samples but was below limit of quantification (less than 0.5 ng/mL), despite having a symmetric peak shape and a matching retention time to the 3-oxo-C6-HSL in the standards. We also observed in many chromatograms’ presence of the peaks with retention time and mass similar to C4-HSL: these peaks at low concentrations exhibited irregular and non-symmetric shape and were below limits of quantification. Therefore, we excluded analysis of C4-HSL in the co-culture supernatants.

**Table 1:** Concentration and type of the identified AHL compounds in the extracts from late exponential growth phase pure cultures of *M. formicicum* and *M. hungatei* and their correspondent co-cultures with *S. fumaroxidans* sampled at late exponential phase of different aggregate maturation stages (Cycles 1 to 33). Biological replicates are presented separately (r1-2). *ND*, not detected, *bLOQ*, below limit of quantification

AHL (ng/mL)	*S. fumaroxidans* + *M. formicicum*								*S. fumaroxidans* + *M. hungatei*								*M.* *formicicum*		*M.* *hungatei*	
	Cycle 1		Cycle 6		Cycle 19		Cycle 29		Cycle 1		Cycle 9		Cycle 22		Cycle 33					
	r1	r2	r1	r2	r1	r2	r1	r2	r1	r2	r1	r2	r1	r2	r1	r2	r1	r2	r1	r2
3-oxo-C6-HSL	bLOQ		bLOQ		bLOQ		ND		bLOQ		bLOQ		bLOQ		bLOQ		bLOQ		bLOQ	
C7-HSL	ND		3.9	4.4	ND		ND		ND		bLOQ		ND		ND		4.2	8.8	7.1	2.7
C8-HSL	ND		6.4	115.5	ND		ND		ND		bLOQ		ND		ND		ND	0.9	ND	0.9
C12-HSL*	ND		4.1	1.3	3.5	ND	ND		ND		180.1	11.7	ND		ND		8.3	8.6	0.5	24.5

*Incomplete dissolution of the C12-HSL compound might lead to an overestimation of the actual quantity in the co-culture extracts, since full dissolution was assumed for calibration curves

### Differential gene expression of the early- and late-aggregation state syntrophic co-cultures

For transcriptome analysis, total RNA was extracted from triplicates of both types of syntrophic co-cultures in the early- (Cycle 1) and late-aggregation states (Cycle 20 for Sf-Mf and Cycle 23 for Sf-Mh).

As a result of mapping the transcriptome reads to the correspondent microorganism, we observed a change in the ratio of the syntroph to methanogen during the co-cultivation and maturation of the aggregates (Table S3). While transcripts affiliated with methanogens were prevalent in either of the co-cultures throughout the co-cultivation, it was especially pronounced in the early-aggregation state (almost 1:4 for syntroph:methanogen ratio). As the co-culture aggregates matured, the number of syntroph-affiliated transcripts increased, especially for the tight aggregates of Sf-Mf (1:1.5 ratio).

When comparing the four sets of transcriptomes from the methanogenic co-cultures, all triplicate samples clustered separately depending on the age (Figure S6) and methanogen used for the co-culture (Figure S7). We observed that expression of *S. fumaroxidans* genes was less affected by the methanogenic partner (5% statistically significantly differentially expressed genes) than by the aggregation state of the co-cultures (20% statistically significantly differentially expressed genes) (Figures S8–S11).

The most prominent changes in the *S. fumaroxidans* gene expression that could be correlated with the methanogenic partner were observed in the late-aggregation state co-cultures. In Sf-Mh Cycle 23 co-cultures, *S. fumaroxidans* had a tenfold statistically significantly upregulated expression of the periplasmic formate dehydrogenase (Sfum_0035-0037) and an operon containing isoquinoline 1-oxidoreductase (Sfum_1729-1732), compared to the Sf-Mf co-cultures at Cycle 20. By comparison, in the Sf-Mf Cycle 20 co-cultures, *S. fumaroxidans* had a tenfold statistically significantly upregulated [FeFe] hydrogenase (Sfum_0843-0848), operons of vitamin B12 transporters (Sfum_0491-0495) and genes for the biosynthesis of tryptophan (Sfum_1771-1778), compared to the Sf-Mh Cycle 23 co-cultures (Supplementary data 1).

**Fig. 4:**
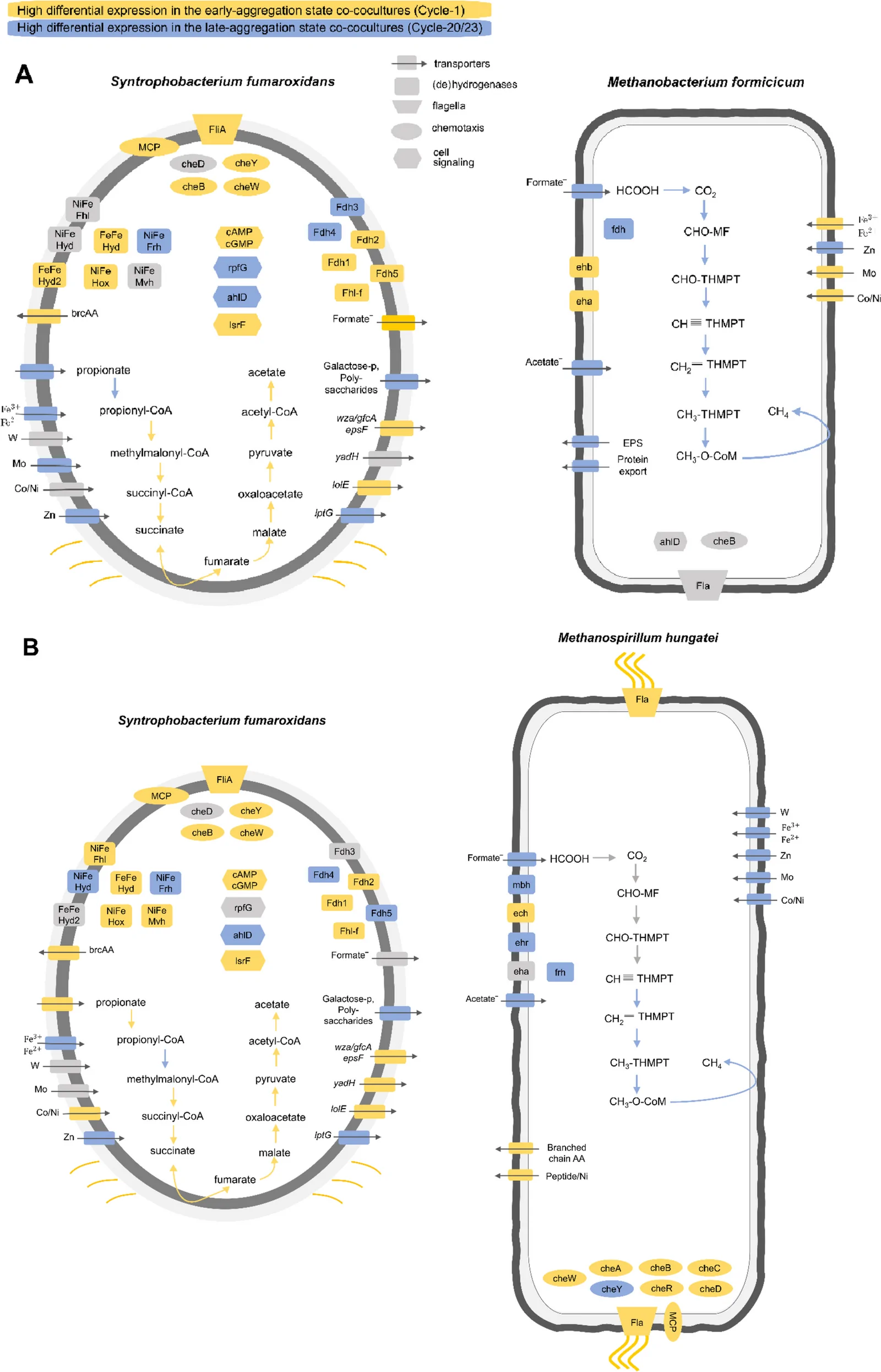
Differentially expressed genes (*p* < 0.01, logFoldChange > 1.5) in the co-cultures of *S. fumaroxidans* with **A** *M. formicicum*, **B** *M. hungatei*. Genes are coloured based on their overexpression in the early- (yellow) or late-aggregation state (blue) co-cultures, else with a comparable expression in both conditions (grey). Carbon utilization pathways (oxidation of propionate and production of methane) have conversion steps coloured based on the differential expression of correspondent genes in one of the conditions. The rest of the genes are grouped into five distinct groups (with markers of different shapes) based on their cellular functionality: transporters, (de)hydrogenases, flagella-associated genes, chemotaxis-associated genes and cell signalling associated genes. Appendages on the cell surface represent pili (in *S. fumaroxidans*) or flagella (*Fla*, *Fli*) and are coloured according to the differential expression in the co-culture condition. Transporter genes include metals transporters (with the correspondent metal labelled on top of the marker), propionate, acetate and formate transporters, branched-chain amino acids (brcAA), peptides and proteins transporters, polysaccharide biosynthesis/export proteins (*wza*/*gfcA*), uncharacterised EPS export genes, genes part of the type II secretion system (*epsF*), galactose transporters, inner membrane transport permeases (y*adH*), lipoprotein-releasing system transmembrane protein (*lolE*), lipopolysaccharide export protein (*lptG*). Metallocluster-defined hydrogenases of *S. fumaroxidans*: membrane-bound *NiFe Fhl*, *NiFe Hyd*, *FeFe Hyd2* and cytoplasmic *FeFe Hyd*, *NiFe Hox*, *NiFe Frh* and *NiFe Mvh*. Membrane-bound formate dehydrogenases of *S. fumaroxidans Fdh2*, *Fdh3*, *Fdh5* and cytoplasmic *Fdh1*, *Fdh4*, *Fhl-f*. Methanogen-specific membrane-bound hydrogenase *mbh*, energy-converting hydrogenase (*eha, ehb, ehr*), NiFe hydrogenase *ech*, coenzyme F420 hydrogenase *frh* and cytoplasmic formate dehydrogenase *frh*. Archaeal flagellar genes clusters of *M. hungatei* and *M. formicicum* (*Fla*); methyl-accepting chemotaxis protein (*MCP*), chemotaxis proteins (*cheABCD*, *cheY*, *cheR*, *cheW*). Cell signalling and quorum sensing associated proteins: cyclic di-GMP phosphodiesterase response regulator (*rpfG*), cyclic nucleotide-binding proteins (*cAMP*, *cGMP*), N-acyl homoserine lactone hydrolase (*ahlD*), 3-hydroxy-5-phosphonooxypentane-2,4-dione thiolase (AI-2 receptor, *lsrF*)

Gene expression of *S. fumaroxidans* in the early- and late-aggregation states in both Sf-Mh and Sf-Mf co-cultures revealed differences in propionate oxidation/methanogenesis metabolisms, metal and amino acid transport and signal transduction depending on the aggregate maturation state (Fig. 4, Figure S7-S15 and Supplementary data 1). Additionally, genes associated with chemotaxis, flagella and pili biosynthesis, production/secretion of polysaccharides and turn-over of quorum sensing-affiliated molecules were also differentially expressed. It is worth noting that a high number of the statistically significantly differentially expressed genes in late-aggregation state co-cultures have unknown function/classification.

## Discussion

In this investigation of the aggregate-forming ability of syntrophic propionate-oxidizing co-cultures, we compare aggregate morphology and biochemical make-up of early- and late-aggregation state co-cultures of *S. fumaroxidans* and methanogens. Although there are episodical reports of aggregate formation in syntrophic propionate-oxidizing co-cultures (de Bok et al. 2002; Worm et al. 2011; Krumholz et al. 2015; Cong et al. 2021), no mechanistic understanding of the phenomenon exists. In this study, we monitored the formation of biofilm aggregates of *S. fumaroxidans* and *M. formicicum* or *M. hungatei* for over 1 year in fed-batch cultivation. Maturation of the co-cultures aggregates enriched for the syntrophic cells, as can be noted from the higher number of *S. fumaroxidans*-specific transcripts in each co-culture in the late-aggregation state (Cycle 20/23), compared to the early-aggregation state co-cultures (Cycle 1). Long-term adaptation to the growth in a syntrophic relationship and *S. fumaroxidans* enrichment in the Cycle 20/23 co-cultures can explain the improved (+30%) propionate oxidation rates compared to the early (Cycle 1) co-cultures. Below we discuss specific differences in the metabolism and gene expression of the two syntrophic co-cultures in early- and late-aggregation stages.

### Propionate oxidation and methanogenesis metabolism

Long-term presence of either of the methanogenic partners with *S. fumaroxidans* led to similar gene expression levels in the main energy metabolism of the syntroph in both late-aggregation state co-cultures (Fig. 4, Figure S12). While genes required for the initial activation of the fatty acid in *S. fumaroxidans* (Sfum_3926-Sfum_3933) were statistically significantly upregulated in the late-aggregation state co-cultures with *M. formicicum*, the rest of the genes encoding enzymes for propionate oxidation via methyl-malonyl pathway were slightly upregulated in the early-aggregation stages with either of the methanogens, compared to the later aggregation stages. Similarly, most of the hydrogenases of *S. fumaroxidans* with either of the methanogens were also slightly upregulated in the early co-cultures, compared to the later ones (expression of the membrane-bound [NiFe] Fhl-h (Sfum_1791-1794), [NiFe] Hox (Sfum_2712-2716), [NiFe] Mvh1,2 (Sfum_3535-3537, Sfum_3954-3957)). However, expression of coenzyme F420-reducing hydrogenases (Sfum_2221-2224; Sfum_3954-3958) was statistically significantly higher in the late-aggregation state Sf-Mf co-cultures, compared to the aggregates from the Cycle 1. Regardless of the aggregate maturation state, *S. fumaroxidans* had a statistically significantly upregulated [FeFe] hydrogenase (Sfum_0843-0848) in the co-cultures with *M. formicicum*, compared to the co-cultures with *M. hungatei* (Supplementary Data 1).

The hydrogen production by the syntroph and its’ expression of hydrogenases (upregulated in the early-aggregation state co-cultures) matches the transcription patterns of the hydrogenases in both methanogens, most of which were statistically significantly upregulated in the early co-cultures, compared to the later ones (Fig. 4, Figure S13, S14). Overall, genes involved in hydrogenotrophic methanogenesis of both *M. hungatei* and *M. formicicum*, as well as archaeal acetate transporters, were upregulated in the late-aggregation state co-cultures. This might suggest an increased need for the carbon source (acetate) for methanogenic cell synthesis in the late aggregated co-cultures.

In both late-aggregation state co-cultures, methanogens also upregulated formate transporters and some formate dehydrogenases (Mhun_0075, Mhun_1811, Mform_01717-01720), while *S. fumaroxidans* upregulated tungsten/molybdenum-containing formate dehydrogenases FDH3, FDH4 (also FDH5 for co-cultures with *M. hungatei*) and a formate transporter (Sfum_2707). Moreover, *S. fumaroxidans* had a significantly upregulated expression of periplasmic formate dehydrogenase (Sfum_0035-0037) in the late-aggregation state co-cultures with *M. hungatei*, compared to the co-cultures with *M. formicicum* (Supplementary Data 1). We hypothesize that upregulated formate transporters and formate dehydrogenases of *M. formicicum* and the syntroph in the late-aggregation state compared to the early-aggregation state could mean that formate transport by the passive diffusion through the cell membrane was not taking place anymore in the late-aggregation state co-cultures, and instead formate transporters were activated to pump formate inside (in case of methanogens) or outside (in case of the syntroph) of the cell. Closer proximity of the methanogenic and syntrophic cells in the aggregates potentially allows a faster formate exchange resulting in lower concentration outside of the cell, making it less favourable for passive diffusion to occur. However, we could not prove this is exactly the case since formate was below detection limit throughout the fed-batch cultivation (even during early cycles).

### Flagella, type IV pili and chemotaxis

The strikingly high expression of *S. fumaroxidans* flagella, type IV pili and chemotaxis genes in the Cycle 1 co-cultures with either of the methanogens (Figure S15) suggests the importance of swimming/swarming functions for the initial contact with methanogens. In addition to this, flagella can also play a role in cell adhesion (Craig et al. 2019). In bacteria and archaea, type IV pili were found to be important for cell-to-cell, cell-to-surface and even cell-to-substrate adhesion (Rakotoarivonina et al. 2002; Pohlschroder and Esquivel 2015; van Wolferen et al. 2018).

In both early-aggregation state Sf-Mh and Sf-Mf co-cultures, *S. fumaroxidans* had a significantly higher expression of the complete set of type IV pili-encoding genes (Figure S15): *pilBMNOQVWXY1Z*, sensor histidine kinase PilS, response regulator PilR and twitching-mobility proteins PilT, MglA (Sfum_2553-2557, 1694, 1695, 0538-0540,0119, 2092). Lower expression of these genes in the late-aggregation state co-cultures might mean pili genes were no longer essential, as “first contact” with the methanogenic partner and cell-to-cell adhesion has already been established. Something similar has been reported in the studies on surface attachment in *Caulobacter crescentus*, where irreversible cell adhesion to the surface resulted in a significantly repressed activity of pili (Ellison et al. 2017). Interestingly, the major pilin protein PilE of *S. fumaroxidans* (Sfum_0126), which was twice more upregulated in the Cycel 1 Sf-Mf co-cultures, has a 64% homology to the geopilin of *Geobacter sulfurreducens* PCA (previously recorded as *pilA*, NCBI accession AAR34870.1). Geopilin of *Geobacter* was shown to be crucial for the cells’ attachment to insoluble Fe (III) oxides and contributed to the extracellular electron transfer (Reguera et al. 2005). Higher differential expression of the gene encoding geopilin (GSU1496) led to a tenfold improved Fe (III) oxide reduction in laboratory-evolved *Geobacter* strains (Tremblay et al. 2011). Whether PilE of *S. fumaroxidans* plays a crucial role in the attachment of the syntroph to the methanogenic cells remains to be tested.

The genome of *M. formicicum* has an incomplete archaella operon, comprised of *flaIJ* (Mform_00687-00688) and *flaK* with a hypothetical flagellin-encoding gene, with amino acid homology to a class III signal peptide (Mform_01543-01544). In other *Euryarchaeota*, FlaK regulates flagellar assembly and FlaI is predicted to have a membrane-spanning C-terminal domain that may interact with the membrane-bound FlaJ (Ghosh and Albers 2011). While *flaIJ* and *flaK* of *M. formicicum* were actively transcribed in both early- and late-aggregation state Sf-Mf, the hypothetical flagellin-encoding gene in the operon with *flaK* was significantly upregulated in the early-aggregation state Sf-Mf co-cultures (Figure S16). In Sf-Mh co-cultures, *M. hungatei* also had an actively transcribed complete operon of archaella genes *flaFGHIJ* (Mhun_0101-0105) and separately encoded three flagellins FlaB (Mhun_1238, Mhun_3139-3140). In early-aggregation state Sf-Mh co-cultures, *M. hungatei* had an upregulated expression *flaF* (Figure S17), which anchors the archaellum in the cell envelope to the archaeal S-layer (Banerjee et al. 2015). In the late-aggregation state co-cultures, on the contrary, the two of archaeal flagellins *flaB* (Mhun_3139-3140) were upregulated, with Mhun_3140 being among the 15 most expressed genes in Sf-Mh co-cultures (Figure S17, Supplementary Data 1). Recent studies have provided evidence that archaellum of *M. hungatei* (comprised of *flaB* gene product of Mhun_3140) has conductive properties (Poweleit et al. 2016; Walker et al. 2019). It is thus possible that aggregation of *M. hungatei* with the syntroph facilitates extracellular or direct electron exchange via archaeal conductive appendages.

Numerous chemotaxis genes of *M. hungatei* (62 in total) were also differentially expressed in either late- or early-aggregation state co-cultures (Figure S18). However, since multiple genes encoded for the homologous proteins, it is hard to evaluate the specific function they have in the two co-culture aggregation stages. The only currently known chemotactic attraction of *M. hungatei* towards acetate (Migas et al. 1989) would be indeed relevant to both aggregation stages, since acetate was present in high quantities throughout the fed-batch cultivation (Fig. 1). For example, after Cycle 4, both methanogenic co-cultures had 16–18 mM acetate at the start of each cycle and 30–35 mM of acetate at the end of the cycle. Thus, it may well be possible that chemotaxis genes were in general highly expressed in *M. hungatei*, regardless of the aggregation progress with *S. fumaroxidans*, albeit the actual function is performed by homologous proteins. Chemotaxis-associated operon containing *cheBYW* and methyl-accepting proteins in *S. fumaroxidans* (Sfum_1645-1652) were also among the highest differentially expressed genes between the two aggregate maturation stages (Figure S15).

### Expression of adhesins and polysaccharide-associated genes

We identified eight adhesins of *S. fumaroxidans* which were upregulated in the late-aggregation state co-cultures (Figure S15). Specifically, genes for fibronectin type III proteins (Sfum_0949, Sfum_2299, Sfum_1109, Sfum_0329) and putative outer membrane adhesin-like proteins (Sfum_2357, Sfum_2359, Sfum_2362) were two- to fourfold higher expressed in the later Sf-Mf and Sf-Mh co-cultures, compared to the early-aggregation state co-cultures. Adhesins and adhesive glycoproteins like fibronectin are large repetitive proteins important for cell-to-cell adhesion and expansion of the bacterial EPS matrix. Deletions of these genes in *Salmonella enterica* resulted in the bacterial phenotypes that are unable to form biofilms (Latasa et al. 2005). It is curious that adhesins of *S. fumaroxidans* were upregulated in the late-aggregation state co-cultures and not in the early ones, where adhesion and establishment of the “first contact” between the cells might be of the primary importance.

The syntroph also had two distinct polysaccharide biosynthesis/export operons that were differentially expressed in either early- (Sfum_2182-2190) or late-aggregation state (Sfum_0972-0979) Sf-Mf and Sf-Mh co-cultures (Figure S15). Operon (Sfum_2182-2190) had several glycosyltransferases, porin and a GDP-L-fucose synthase, while operon Sfum_0972-0979 contained another set of polysaccharide biosynthesis/export proteins (with Cps/CapB family tyrosine kinase involved in the biosynthesis of capsular polysaccharide).

Transcriptome of *M. hungatei* revealed a full pathway for the biosynthesis of the production of UDP-N-acetyl-D-glucosamine from α-D-glucose 6-phosphate (Mhun_2600, Mhun_2852-2855) (Figure S17). All these genes (except *glmM*, phosphoglucosamine mutase, Mhun_2852) were upregulated in the Cycle 1 Sf-Mh co-cultures, compared to the Cycle-23 co-cultures. Instead, the Cycle 23 Sf-Mh co-cultures, *M. hungatei* significantly upregulated expression of the major sheath protein MspA (Mhun_2271), which was the third most highly expressed gene in this condition (Figure S14, Supplementary Data 1).

EPS-associated genes of *M. formicicum*, like genes for the biosynthesis of GDP-D-rhamnose, were mostly upregulated in the late-aggregation state Sf-Mf co-cultures (Figure S16). Notably, in Cycle 20 Sf-Mf aggregates *M. formicicum* had a fourfold higher expression of a large OmcB-like cysteine-rich periplasmic protein with conserved DUF11 domain (Mform_01534, Figure S13). This protein is hypothesized to play a key role as a membrane-bound adhesion protein involved in maintaining cell aggregates (Sumikawa et al. 2019).

### Cell signalling

Since microbial aggregation and biofilm formation is a coordinated microbial process, it was not surprising to see a high expression of the genes involved in the intra- and intercellular signalling (Figure S15, S16, S17). Specifically, Sf-Mh and Sf-Mf co-cultures had differentially expressed *S. fumaroxidans* genes involved in the cycling of intracellular signalling molecule bis-(30–50)-cyclic dimeric guanosine monophosphate (c-di-GMP) (Figure S15). While most of the genes potentially involved in the sensing and synthesis of c-di-GMP (Sfum_0719, Sfum_1516, Sfum_1918, Sfum_2650, Sfum_1020, Sfum_2209) were upregulated in the early-aggregation state co-cultures, a few diguanylate cyclases (Sfum_0975, Sfum_3257) and associated regulatory sensory histidine kinases in polysaccharide biosynthesis operons (Sfum_2621-2622) were upregulated in the late-aggregation state co-cultures (Figure S15). Similar expression pattern for c-di-GMP-associated genes was previously reported for another sulfate reducer, *Desulfovibrio vulgaris* Hildenborough, where expression of diguanylate cyclases was found to be essential for *D. vulgaris* Hildenborough optimal growth and biofilm forming capability (Rajeev et al. 2014). High concentrations of intracellular c-di-GMP can promote biofilm formation by inducing the synthesis of exopolysaccharides and adhesins, while inhibiting motility and activity of flagella (Hengge 2009; Rajeev et al. 2014). Unfortunately, we are currently lacking the measurements of the intracellular concentrations of c-di-GMP in the Sf-Mf and Sf-Mh co-cultures to explicitly correlate the observed gene expression profiles with the co-culture aggregation status.

We were, however, able to detect varying extracellular concentrations of the signalling molecules involved in the intercellular signalling, AHLs, during the year of Sf-Mf and Sf-Mh fed-batch cultivation (Table 1). The concentrations of the C8- and C12-HSL in the co-cultures supernatants fit well into the range reported for the extracts from pure culture of *Methanosaeta harundinacea* 6Ac (Zhang et al. 2012) where concentrations of a β-ketooctanoyl-L-homoserine lactone homologue ranged from 24 ng/mL to 6.5 μg/mL. In Sf-Mf and Sf-Mh co-cultures, the three AHLs (C7-, C8- and C12-HSL) were only detectable in the later cultivation stages (Cycle 6 in Table 1). It is possible, however, that the observed here concentrations are sufficiently high to activate the bacterial adhesion or biofilm formation, as they were above the stimulatory 10 ng/mL threshold reported for the aggregation of the microorganisms in activated sludge (Wang et al. 2021). These three AHLs were below detection limit in the early co-cultures (Cycle 1) or in the co-cultures that already had macroaggregates (after Cycle 13, Table 1). On the contrary, 3-oxo-C6-HSL was detectable throughout the development of the aggregates but was always below limit of quantification (Table 1, Table S2).

Contrary to the case of intracellular signalling and identification of genes related to cycling of intracellular c-di-GMP, search for the genes with intercellular AHL sensing/synthesis domains was not trivial. We were not able to find any genes homologous to the known AHL synthases (Rosemeyer et al. 1998) in either of the three studied here microorganisms. A single report exists to date describing the ability of a methanogen, *M. harundinacea* 6Ac, to synthesize a QS molecule structurally similar to C12-HSL (N-carboxyl-C_12_-HSL) (Zhang et al. 2012). The genomes of all three microorganisms studied here possess multiple homologs to the novel identified AHL synthase in *M. harundinacea* 6Ac, currently annotated as PAS domain S-box proteins, with up to 30% protein identity. However, none of those PAS domain S-box proteins are homologous to the LuxI autoinducer synthase of *Erwinia chrysanthemi* (GenBank accession number AAM46699), which was used to probe for the AHL synthase in *M. harundinacea* 6Ac study.

We did identify genes needed for sensing of the extracellular AHL and autoinducer-2 (AI-2) in *S. fumaroxidans* and *M. formicicum*. In both Cycle 1 Sf-Mf and Sf-Mh co-cultures, *S. fumaroxidans* had an upregulated expression of an AI-2 receptor (3-hydroxy-5-phosphonooxypentane-2,4-dione thiolase, lsrF, Sfum_2464) that can be involved in the degradation of phosphor-AI-2 molecule. To contrast, genes for the AHL hydrolysis (*ahlD*, Sfum_3579) were slightly upregulated in the late-aggregation state co-cultures (1.5–2-fold, Figure S15). An AHL hydrolase, homologous to the known bacterial ones, was also found in *M. formicicum* (Mform_01488) but was also only slightly upregulated in the late-aggregation state Sf-Mf co-culture (less than 1.5-fold, Figure S16). Therefore, it is possible that decreased levels of AHLs reported in Table 1 might be explained by the activity of these AHL hydrolases (Mform_01488 and Sfum_3579) in the late-aggregation state Sf-Mf co-cultures.

Apart from genes associated with the turn-over of the known signalling molecules like AHLs and c-di-GMPs, all three microorganisms studied here possess numerous poorly characterized genes with predicted cell signalling-associated functions. Both methanogens had highly upregulated peptide transporter systems and “autotransporter porins” in the late-aggregation state co-cultures (“Secretion” set for *M. formicicum* on Figure S16, “Signal transduction” set for *M. hungatei* on Figure S18). These proteins might be involved in the extracellular exchange of information, potentially cross-kingdom (Li et al. 2018). Porins of *M. formicicum* had a conserved DUF11 domain (Mform_00118, Mform_01307, Mform_01534, Mform_02114), hypothesized to be a part of the archaeal S-layer structure. DUF11-containing proteins were previously reported to play a key role in stabilizing the cell aggregates in *Methanothermobacter* sp. CaT2 (Sumikawa et al. 2019). Considering that pure cultures of *M. formicicum* tend to self-aggregate, we can hypothesize that DUF11 containing proteins in this microorganism might be also involved in the stabilization of the aggregating cells. This hypothesis is plausible since the most differentially and highly expressed protein of *M. formicicum* in the late-aggregation state Sf-Mf co-cultures, Mform_01534, has a 41% protein identity to the aggregation-defining protein of *Methanothermobacter* sp. CaT2 (GenBank WP_158498096.1).

Transcriptome of *S. fumaroxidans* had a few poorly characterized signalling genes, like outer membrane adhesin-like proteins Sfum_2357 and Sfum_2359, which were upregulated in the later aggregated co-cultures and might be involved in the recognition of the syntroph by a partner methanogen (Figure S15). However, amino acid sequences of these genes had only a low homology (15%) to the PilD of another syntroph, *Pelotomaculum thermopropionicum*, where it was reported to directly activate expression of methanogenesis genes and hydrogenases in the methanogenic partner *Methanothermobacter thermautotrophicus* (Shimoyama et al. 2009). Apart from these genes, *S. fumaroxidans* had a few other genes potentially needed for the partner recognition and characterized to produce cell surface glycoprotein-containing proteins (Sfum_0949, 0329, 0804). All three were highly upregulated and differentially expressed in both late-aggregation state Sf-Mf and Sf-Mh co-cultures. However, more experimental evidence is needed to pinpoint the exact role of these signal transduction/receiving proteins.

With this morphological and biochemical study of the syntrophic aggregates, we set the stage for the exploration of this heavily understudied field of suspended methanogenic biofilms. Knowing how methanogenic communities switch and adapt to the attached/aggregated lifestyle greatly improves our fundamental understanding of these ubiquitous microbial communities that live at the lowest thermodynamic energy limits.

## Supplementary information

ESM 1 (PDF 4193 kb)

ESM 2 (XLSX 6344 kb)

## Data Availability

All data supporting the findings of this study are available within the paper and its two Supplementary Information files. Processed and normalized RNA-seq data is available in the Supplementary file 2 (spreadsheet).
