# Supplementary material for: Methanogenic partner influences cell aggregation and signalling of Syntrophobacterium fumaroxidans
Source: Appl Microbiol Biotechnol. 2024 Jan 13;108:127. doi: 10.1007/s00253-023-12955-w (PMC10787695; doi:10.1007/s00253-023-12955-w)
Supplement: Supplementary file 1 [file 253_2023_12955_MOESM1_ESM.pdf]

SUPPORTING INFORMATION

Applied Microbiology and Biotechnology

Methanogenic partner influences cell aggregation and signaling of *Syntrophobacterium fumaroxidans*

Doloman, Anna[1*]; Besteman, Maaike S[1].; Sanders, Mark G[2].; Sousa, Diana Z[1,3].

[1] Laboratory of Microbiology, Wageningen University & Research, Stippeneng 4, 6708 WE Wageningen, The Netherlands

[2] Laboratory of Food Chemistry, Wageningen University, Bornse Weilanden 9, 6708 WG Wageningen, The Netherlands

[3] Centre for Living Technologies, Eindhoven-Wageningen-Utrecht Alliance, Princetonlaan 6, 3584 CB Utrecht, The Netherlands

* Correspondent author, anna.doloman@wur.nl

Number of pages: 43

Number of figures: 18

Number of tables: 3

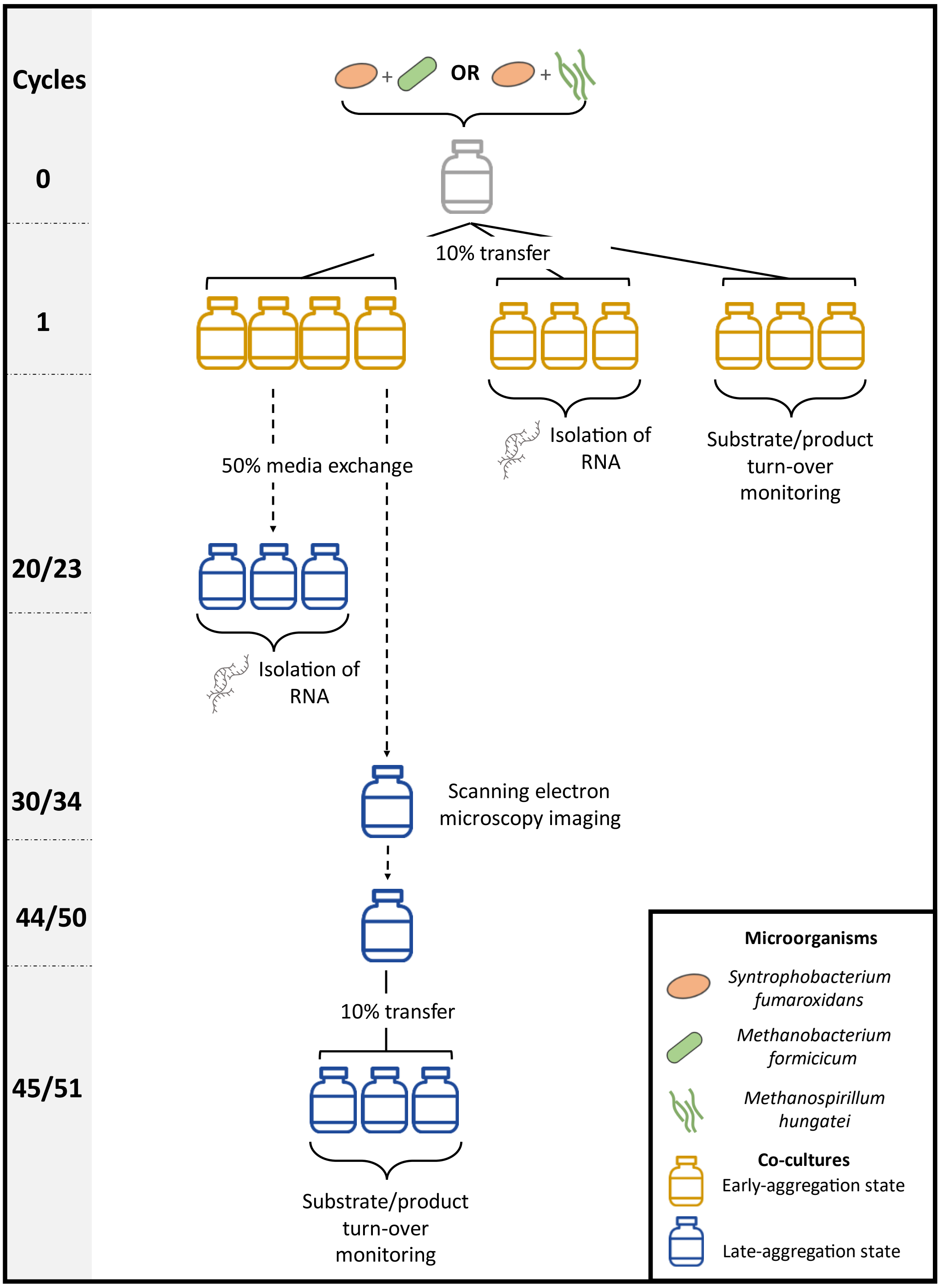

Figure S1. General scheme for the maintenance and sampling of the co-cultures *of Syntrophobacterium fumaroxidans* and two methanogens, *Methanobacterium formicicum* and *Methanospirillum hungatei*.

Table S1. List of the N-Acyl homoserine lactones used as standards for the UHPLC-MS/MS assays.

**Abbreviation**	**Full name**	**Chemical formula**	**Mass accuracy, [M+H] +, m/z**
C4-HSL	N-Butyryl-DL-homoserine lactone	$C_8H_{13}NO_3$	172.09669
C6-HSL	N-Hexanoyl-L-homoserine lactone	$C_{10}H_{17}NO_3$	200.12773
3-oxo-C6-HSL	N-(Ketocaproyl)-d,l-homoserine lactone	$C_{10}H_{15}NO_4$	214.10733
C7-HSL	N-Heptanoyl-DL-homoserine lactone	$C_{11}H_{19}NO_3$	214.14377
C8-HSL	N-Octanoyl-L-homoserine lactone	$C_{12}H_{21}NO_3$	228.15944
3-oxo-C10-HSL	N-(3-Oxodecanoyl)-L-homoserine lactone	$C_{14}H_{23}NO_4$	270.16977
C12-HSL	N-Dodecanoyl-L-homoserine lactone	$C_{16}H_{29}NO_3$	284.22202

Table S2. AHL standards injected and analyzed in UHPLC-MS/MS in the SIM mode (170-450 m/z mass window) with correspondent concentration ranges, standard curves values (ax+b; R^2), retention times, limits of detection (LOD) and limits of quantification (LOQ).

**Compound**	**Concentration range, ng/mL**	**ax+b**	**R^2**	**Retention time, min**	**LOD, ng/mL**	**LOQ, ng/mL**
C4-HSL	0.832 – 53.949	41436x + 2242	0.994	3.55 ± 0.14	0.538	1.614
C6-HSL	0.499 – 33.034	258877x + 89079	0.995	8.98 ± 0.15	0.291	0.873
3-oxo-C6-HSL	0.548 – 36.304	181258x + 84709	0.990	5.22 ± 0.3	0.457	1.371
C7-HSL	0.524 – 34.695	213360x + 208655	0.998	11.9 ± 0.14	0.219	0.656
C8-HSL	0.538 – 35.654	267132x + 143344	0.998	14.53 ± 0.14	0.203	0.608
3-oxo-C10-HSL	0.676 – 44.798	341526x + 90538	0.999	16.14 ± 0.42	0.142	0.427
C12-HSL	0.548 – 36.304	140954x + 138123	0.999	22.87 ± 0.16	0.177	0.530

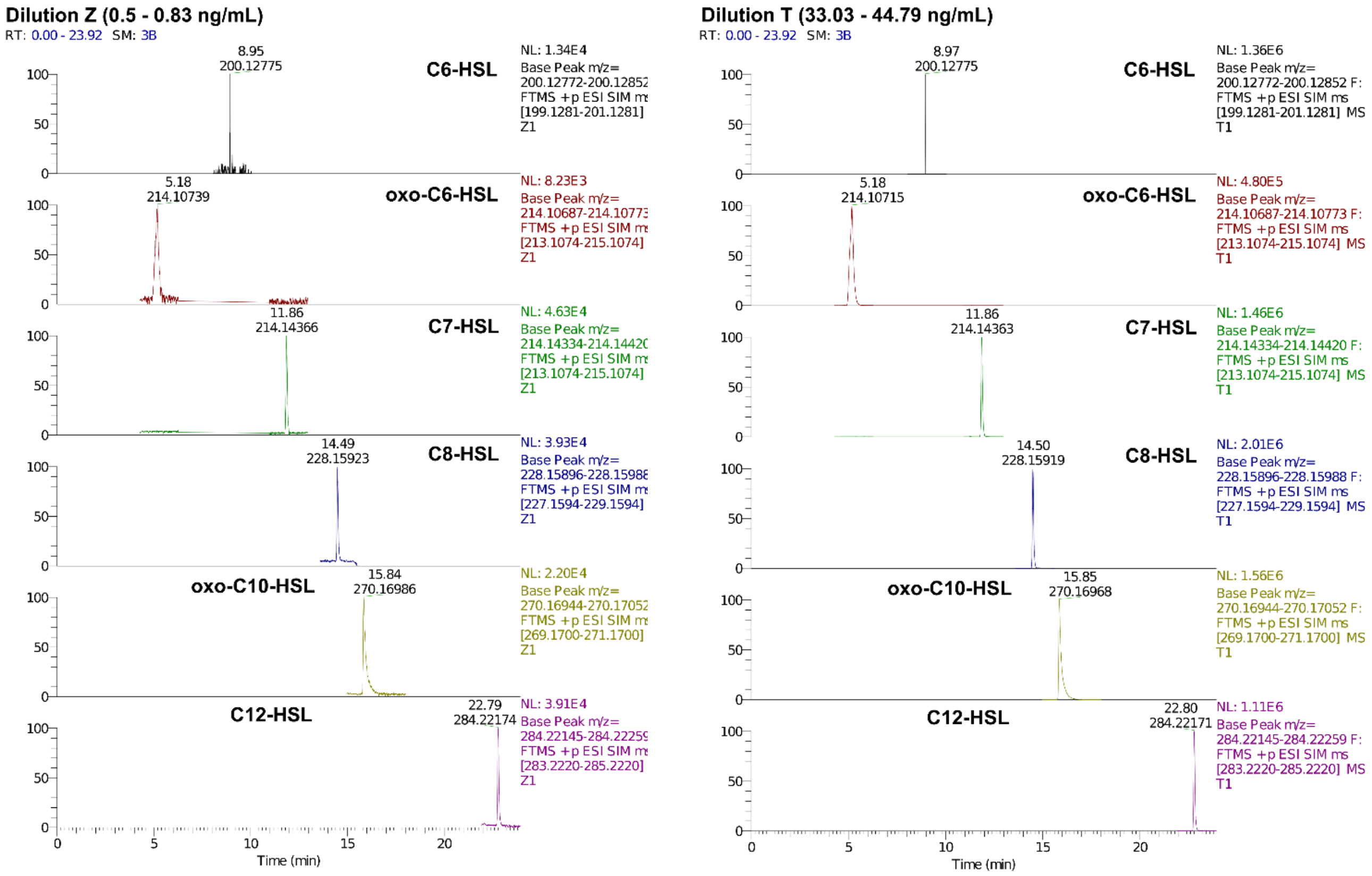

Figure S2. Chromatograms of AHLs analyzed in SIM mode on UHPLC-MS/MS. Two concentrations were chosen as an example: the smallest (Z, left) and the highest (T, right).

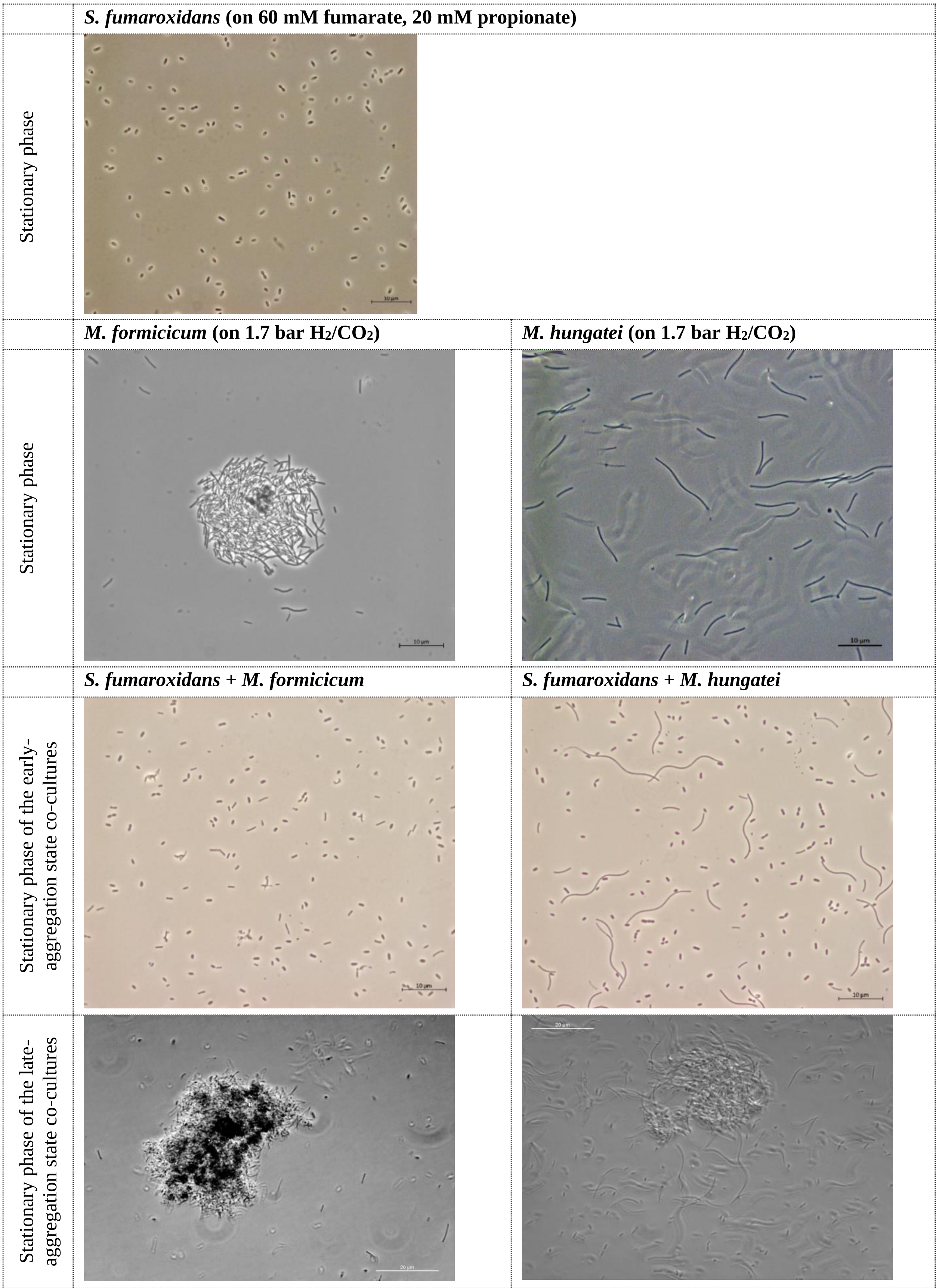

Figure S3. Light microscopy of the pure cultures (A) of *S. fumaroxidans*, *M. formicicum*, and *M. hungatei* and their respective co-cultures (B) at early- and late-aggregation states. Late-aggregation state Sf-Mf co-cultures

were viewed at Cycle-8 and Sf-Mh at Cycle-13. Scale bars are 10 μM in all the images, except for the bottom ones of co-cultures in the late-aggregation state (20 μM instead).

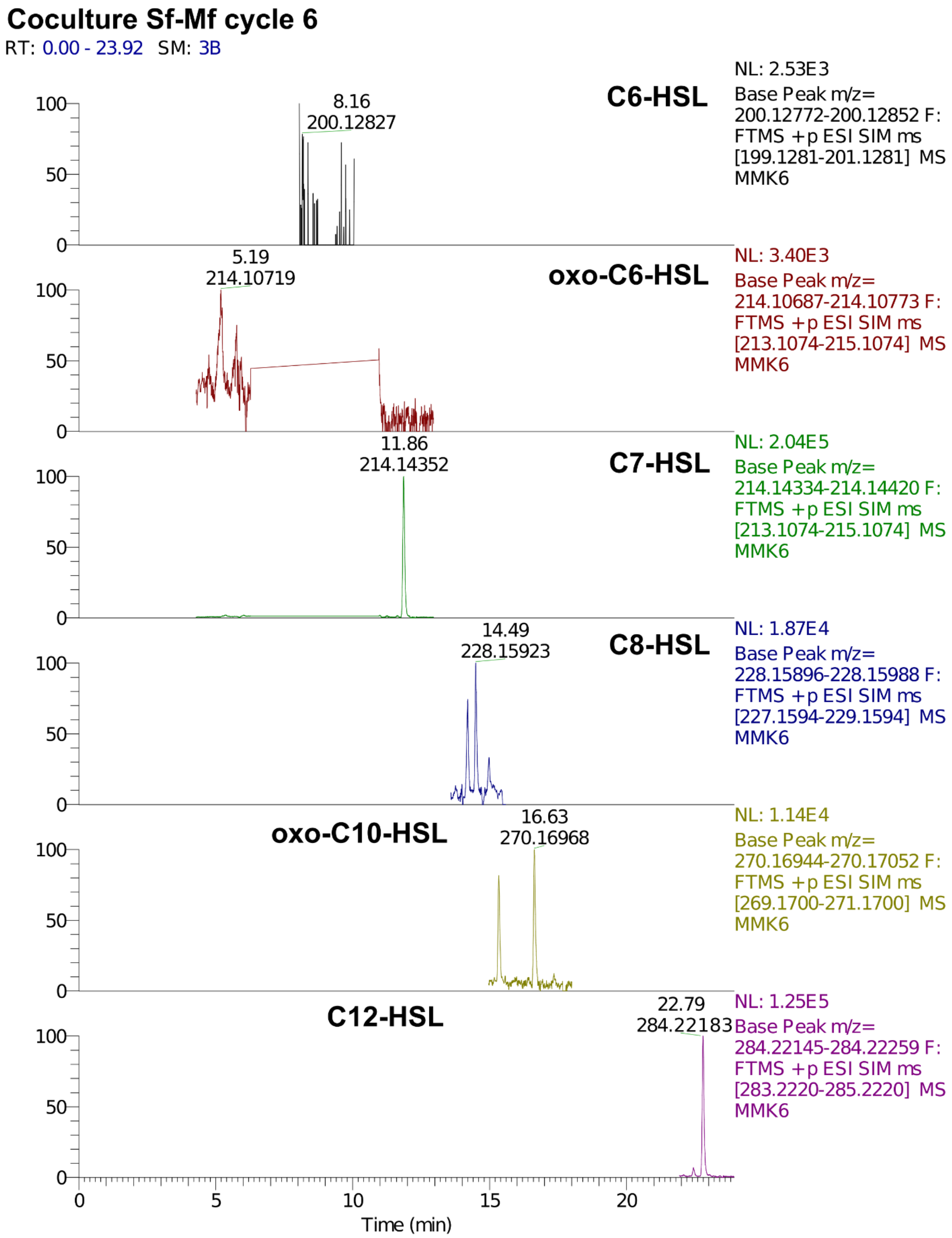

Figure S4. Chromatograms of analyzed *S. fumaroxidans* and *M. formicicum* co-culture supernatants from Cycle-6 in SIM mode on UHPLC-MS/MS.

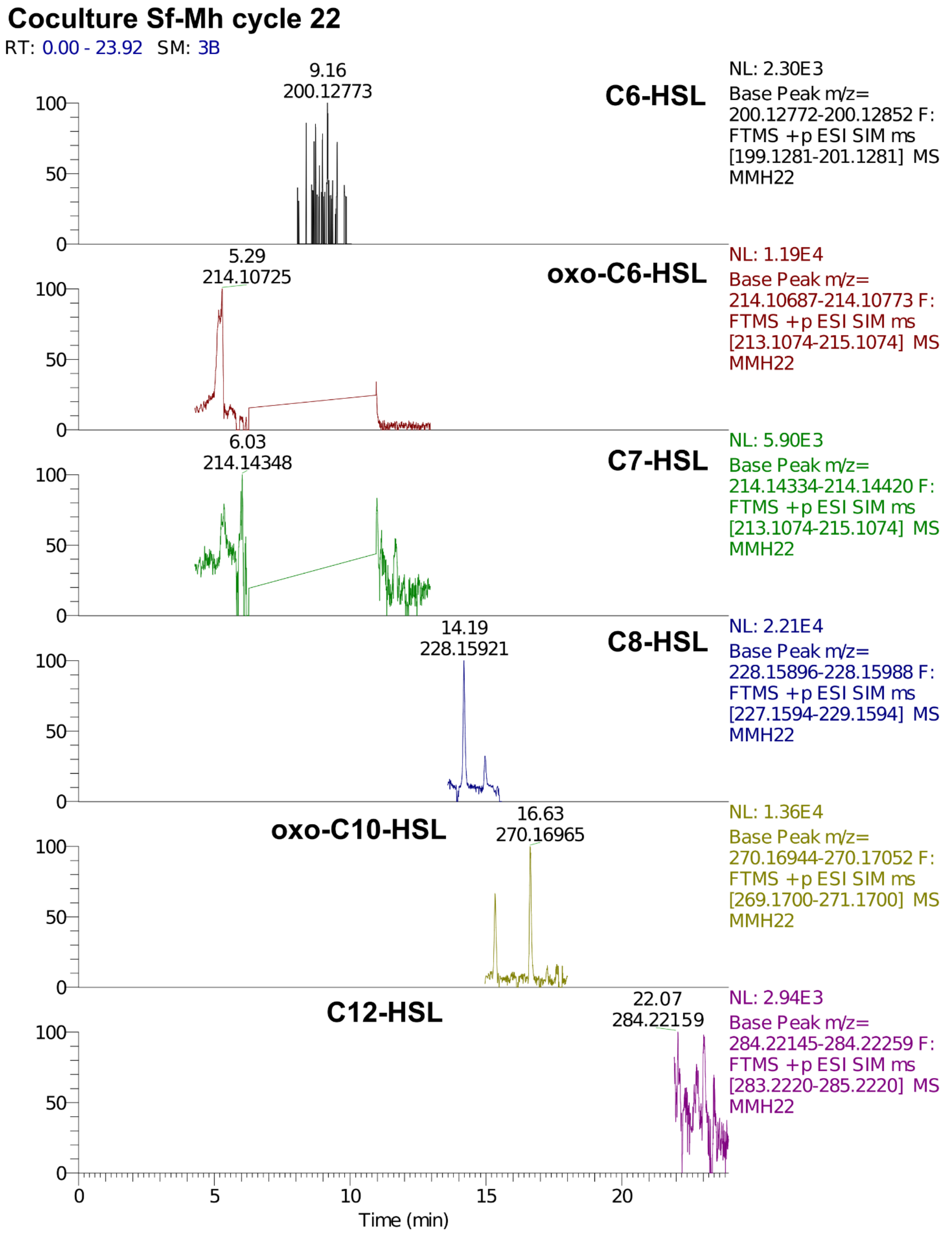

Figure S5. Chromatograms of analyzed *S. fumaroxidans* and *M. hungatei* co-culture supernatants from Cycle-22 in SIM mode on UHPLC-MS/MS.

Table S3. Transcriptome reads from the triplicates of the co-cultures of *S. fumaroxidans* with *M. formicicum* (Sf_Mf) or *M. hungatei* (Sf_Mh) at the early- (Cycle-1) or late-aggregation (Cycle-20/23) states.

Sample	Replicate	% *S. fumaroxidans* reads	% Methanogen reads
Sf-Mf (cycle-1)	1	27.7	72.3
	2	29.9	70.0
	3	29.3	70.7
Sf-Mf (cycle-20)	1	38.1	61.9
	2	42.0	58.0
	3	42.7	57.3
Sf-Mh (cycle-1)	1	11.3	88.7
	2	12.4	87.6
	3	12.5	87.5
Sf-Mh (cycle-23)	1	23.2	76.8
	2	18.8	81.2
	3	18.5	81.5

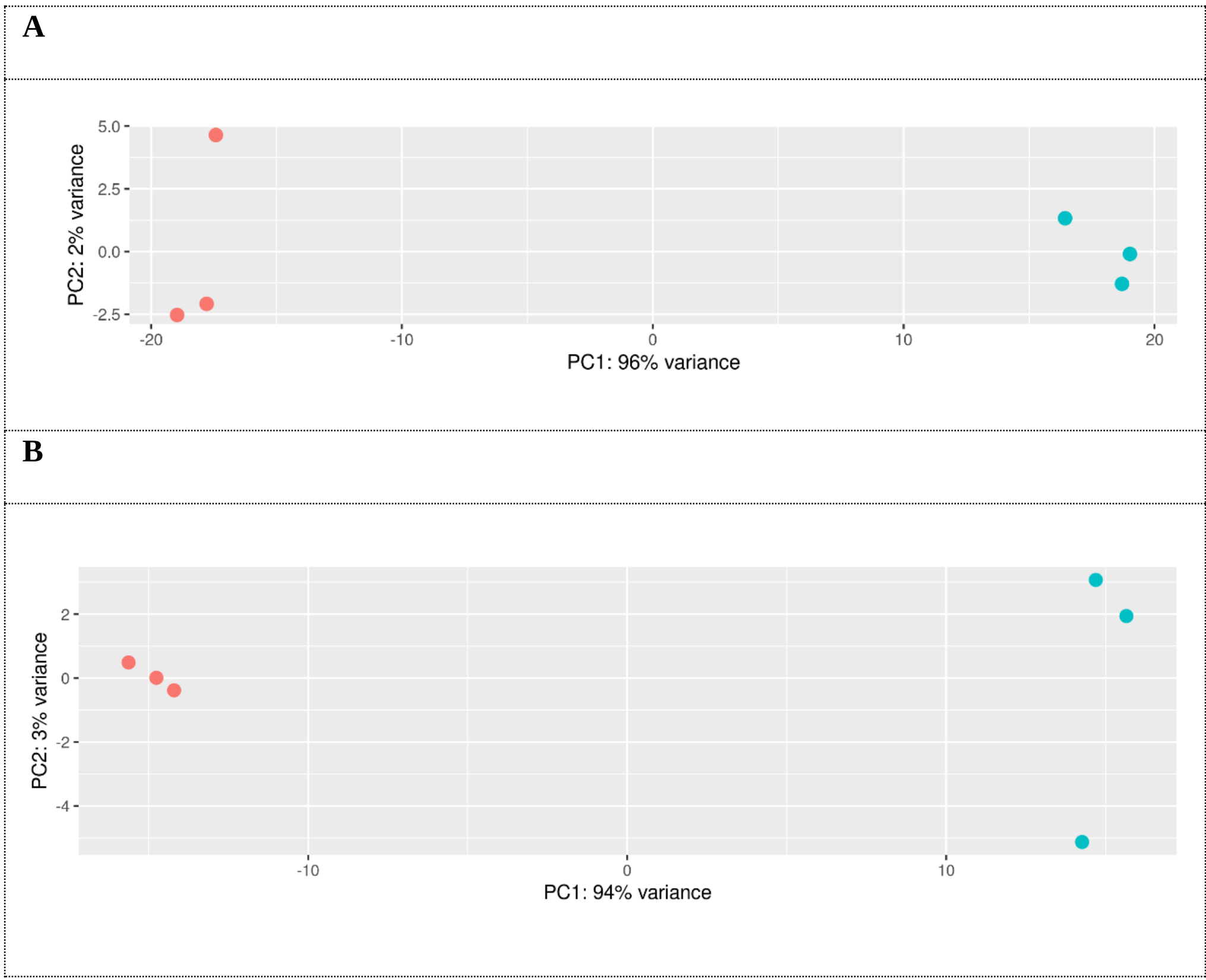

Figure S6. Principal component analysis of the transcriptome samples from the co-cultures of *S. fumaroxidans* with *M. formicium* (A) or *M. hungatei* (B) at the early-aggregation (orange) or late-aggregation (green) state.

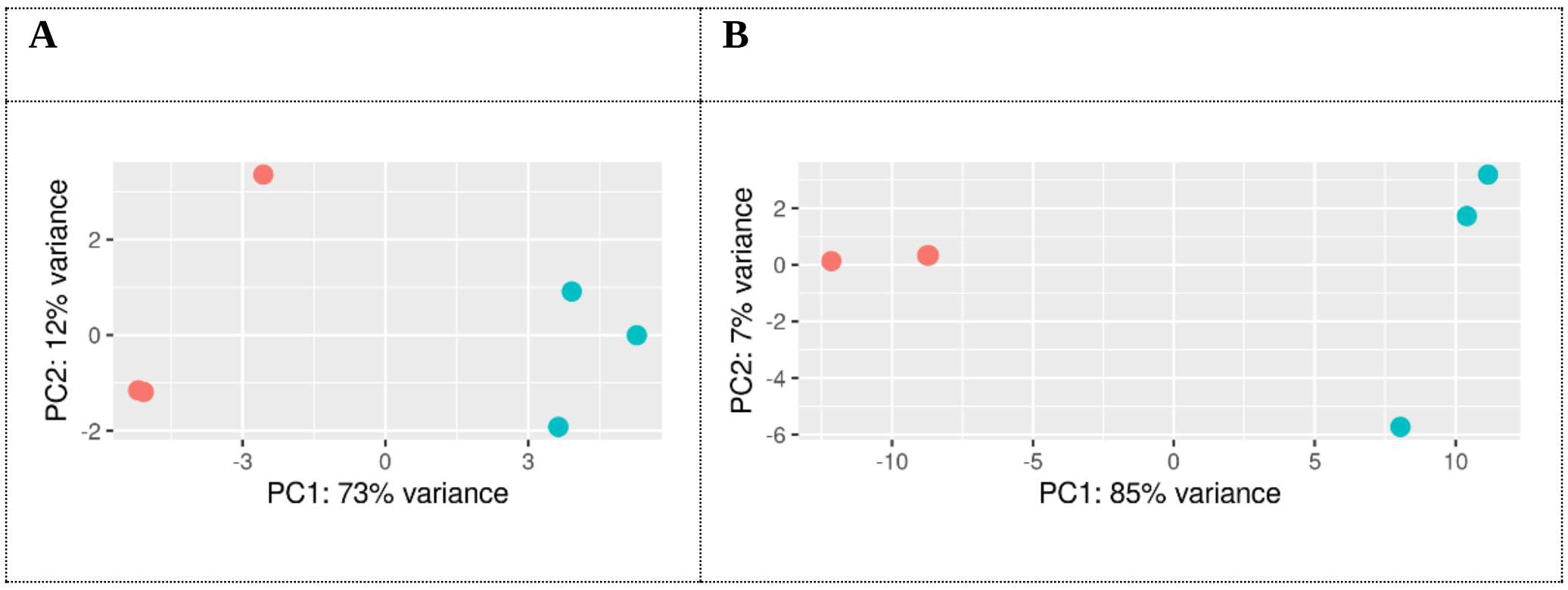

Figure S7. Principal component analysis of the transcriptome samples from the co-cultures of *S. fumaroxidans* with *M. formicium* (orange) or *M. hungatei* (green), with respect to the *S. fumaroxidans* genes at the early-aggregation (A) or late-aggregation (B) state.

Late-aggregation state	Early-aggregation state

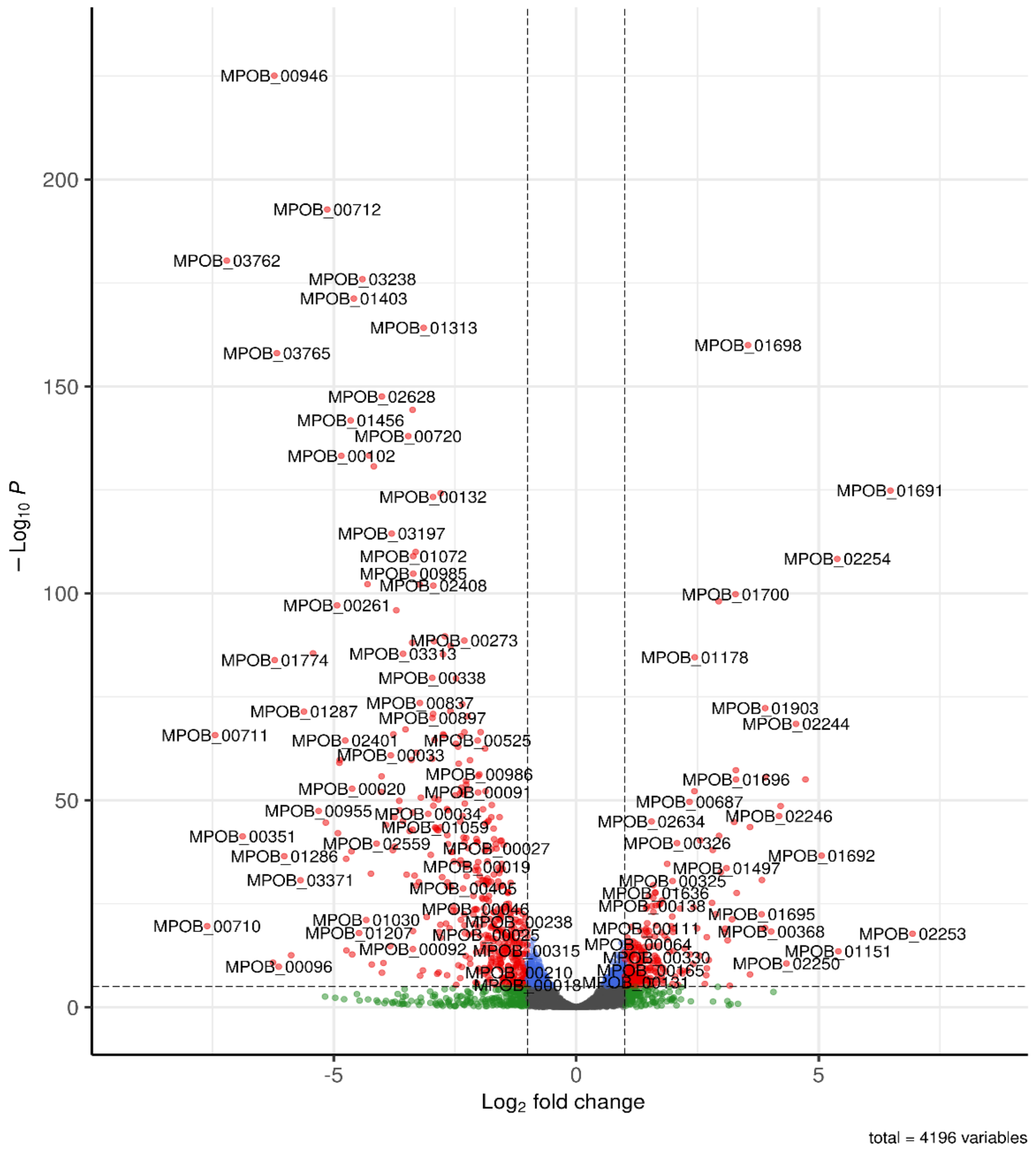

Figure S8. Differential expression of the *S. fumaroxidans* genes in the co-culture with *M. formicicum* in the early- (right) VS late-aggregation state (left). Genes labelled as red are statistically significantly overexpressed in one of the co-culture conditions (Log2FC >1, P_{adj} = 0.001, see Materials and Methods for details).

**Late-aggregation state**	**Early-aggregation state**

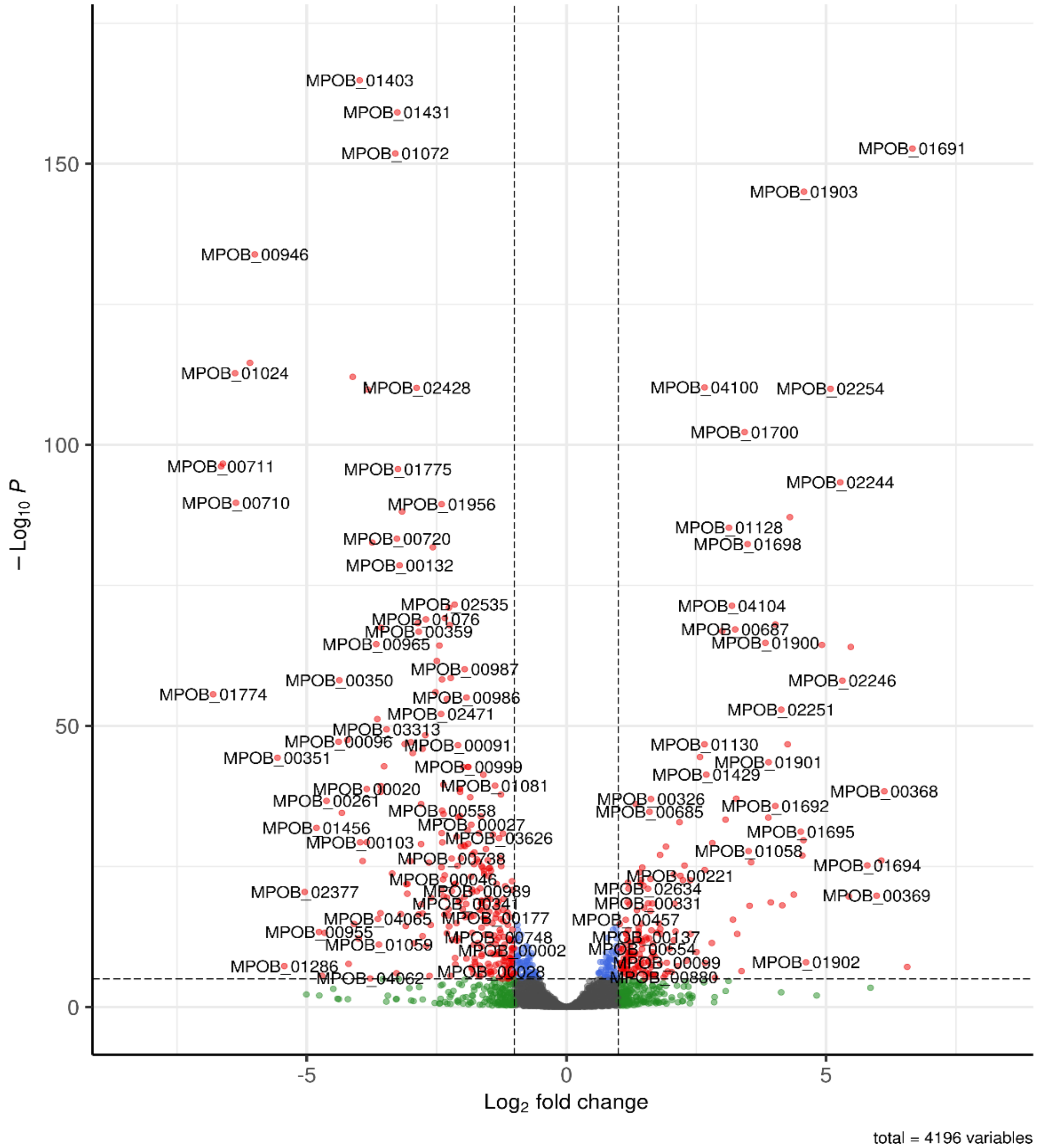

Figure S9. Differential expression of the *S. fumaroxidans* genes in the co-culture with *M. hungatei* in the early- (right) VS late-aggregation state (left). Genes labelled as red are statistically significantly overexpressed in one of the co-culture conditions (Log2FC >1, P_{adj} = 0.001, see Materials and Methods for details).

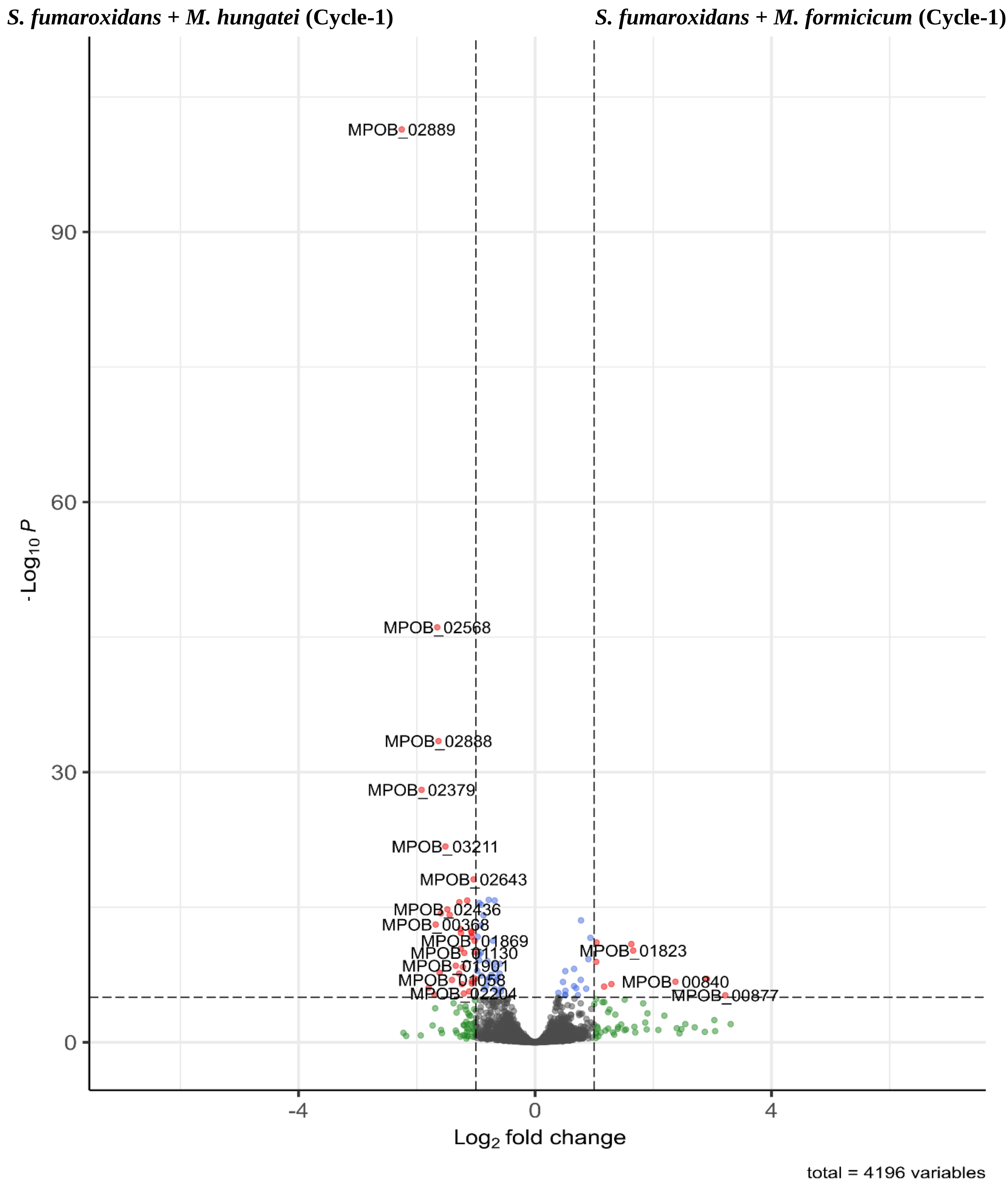

Figure S10. Differential expression of the *S. fumaroxidans* genes in the co-culture with *M. formicicum* (right) or *M. hungatei* (left) in the Cycle-1 co-cultures. Genes labelled as red are statistically significantly overexpressed in one of the co-cultures (Log2FC >1, P_{adj} = 0.001, see Materials and Methods for details).

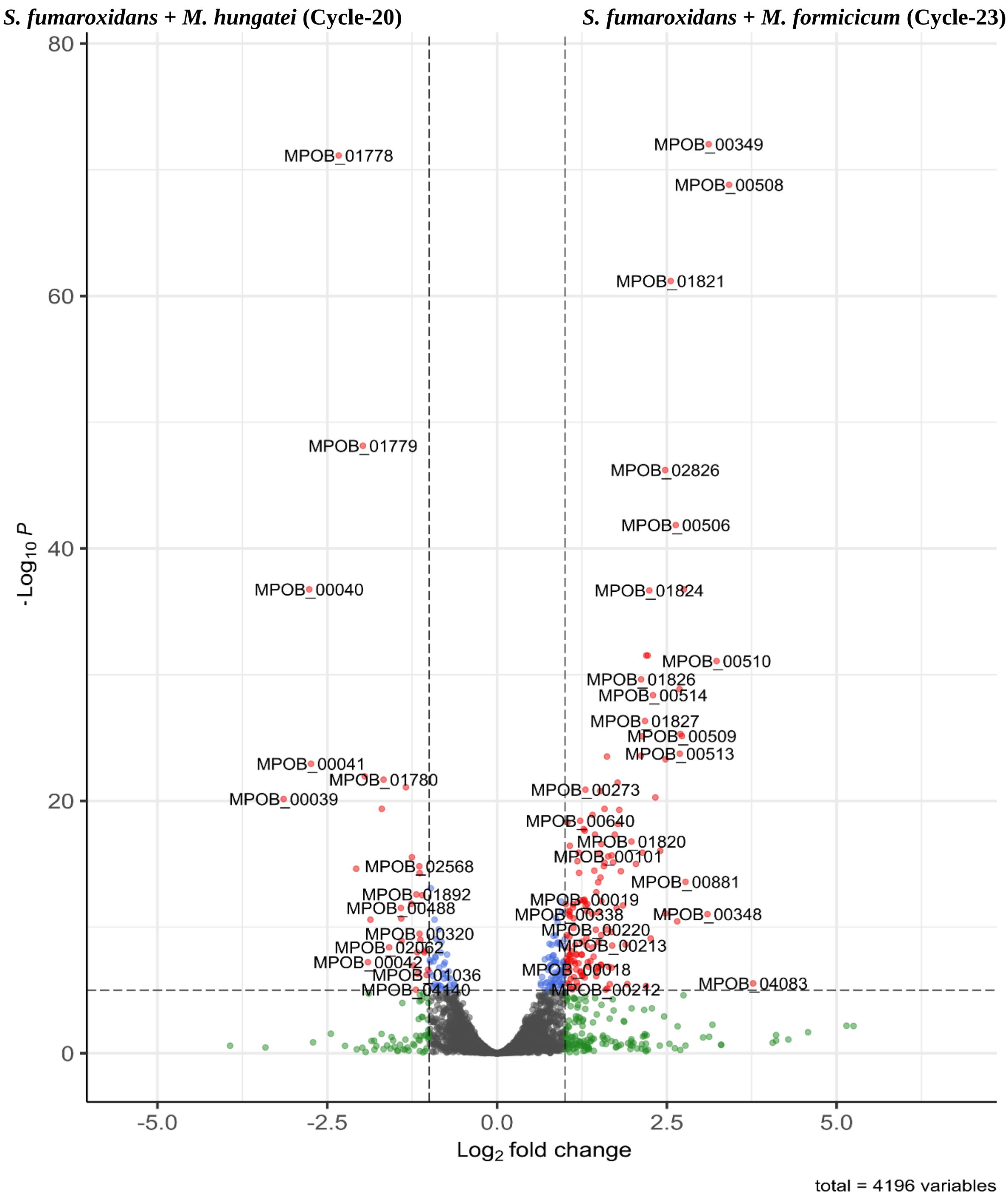

Figure S11. Differential expression of the *S. fumaroxidans* genes in the co-culture with *M. formicicum* (right) or *M. hungatei* (left) in the Cycle-20/23 co-cultures. Genes labelled as red are statistically significantly overexpressed in one of the co-cultures (Log2FC >1, P_{adj} = 0.001, see Materials and Methods for details).

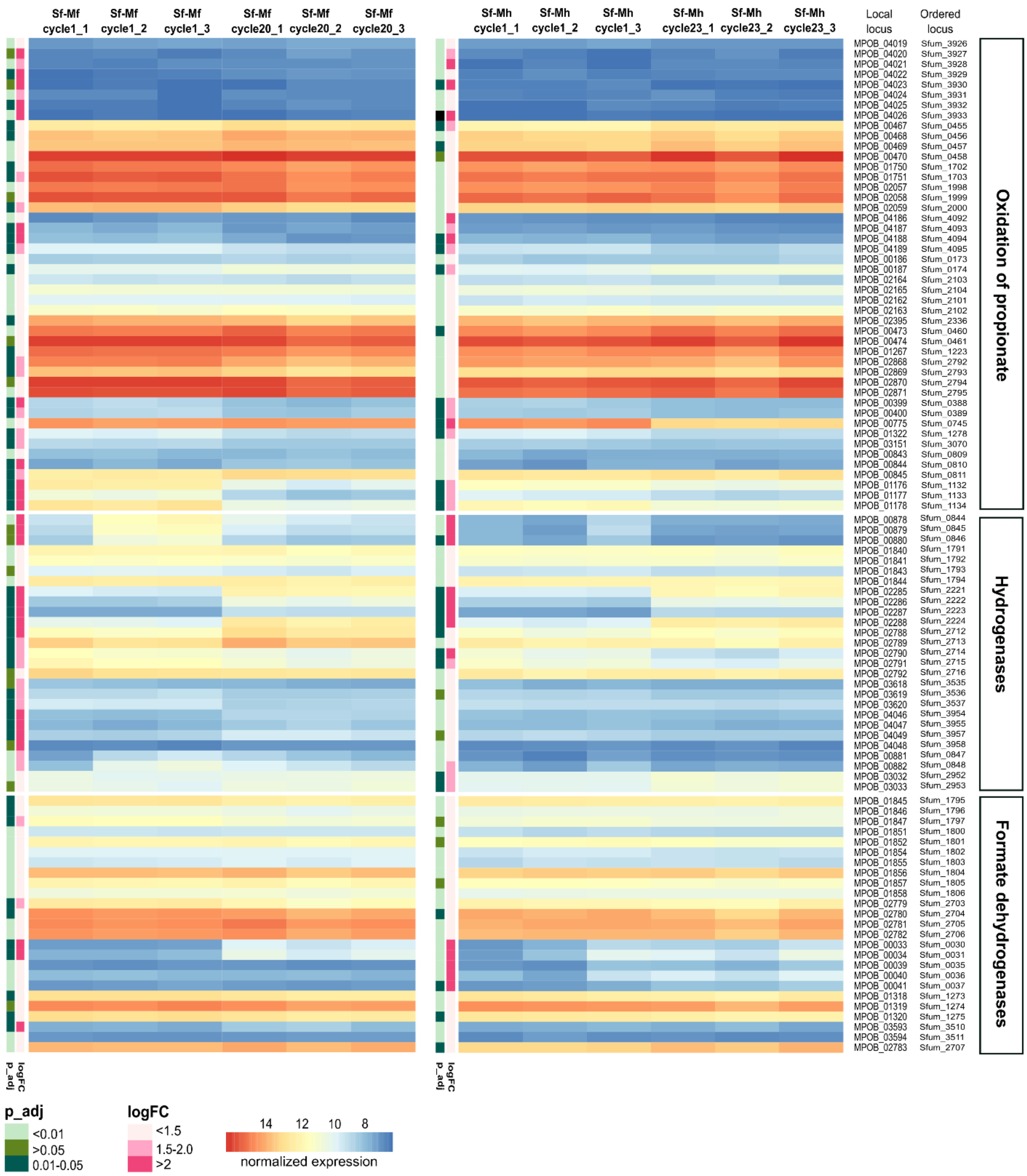

Figure S12. A normalized differential expression of the *S. fumaroxidans* genes for the central metabolism in Sf-Mf and Sf-Mh early- (Cycle-1) and late-aggregation (Cycles-20/23) state co-cultures.

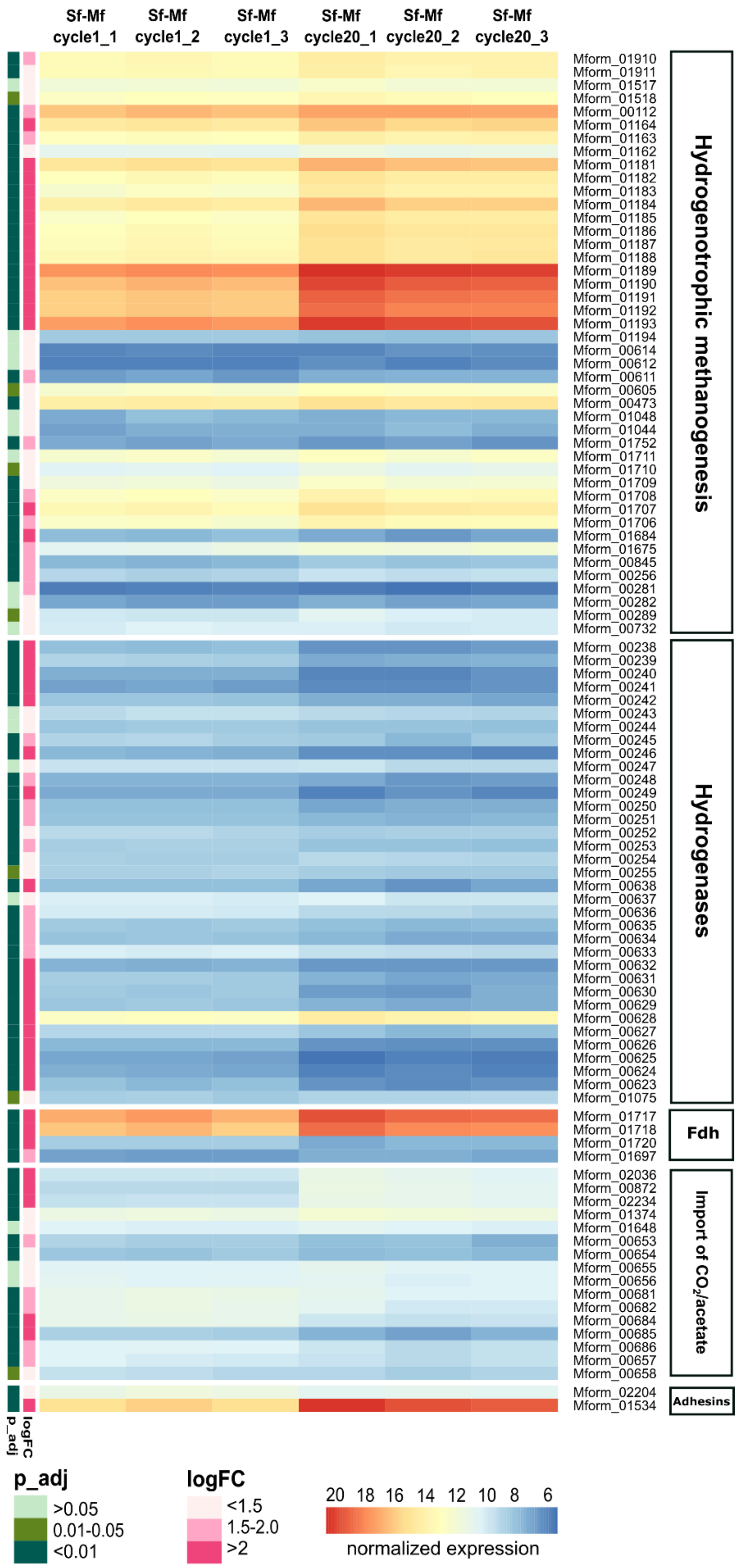

Figure S13. A normalized differential expression of the *M. formicicum* genes for the central metabolism in Sf-Mf early- (Cycle-1) and late-aggregation (Cycle-20) state co-cultures. Fdh = formate dehydrogenases.

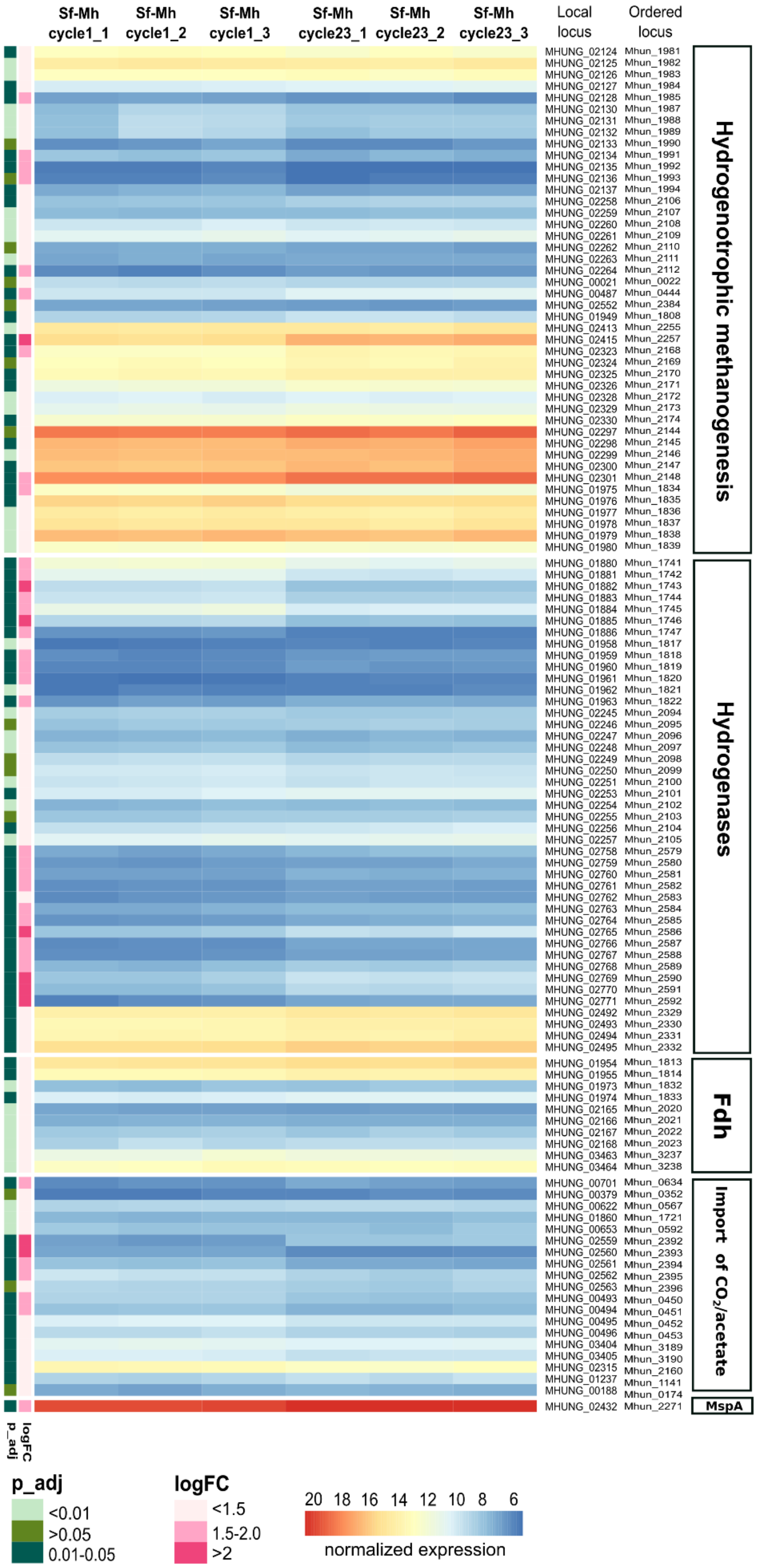

Figure S14. A normalized differential expression of the *M. hungatei* genes for the central metabolism in Sf-Mh early- (Cycle-1) and late-aggregation (Cycle-23) state co-cultures. Fdh: formate dehydrogenases, MspA: major sheath protein.

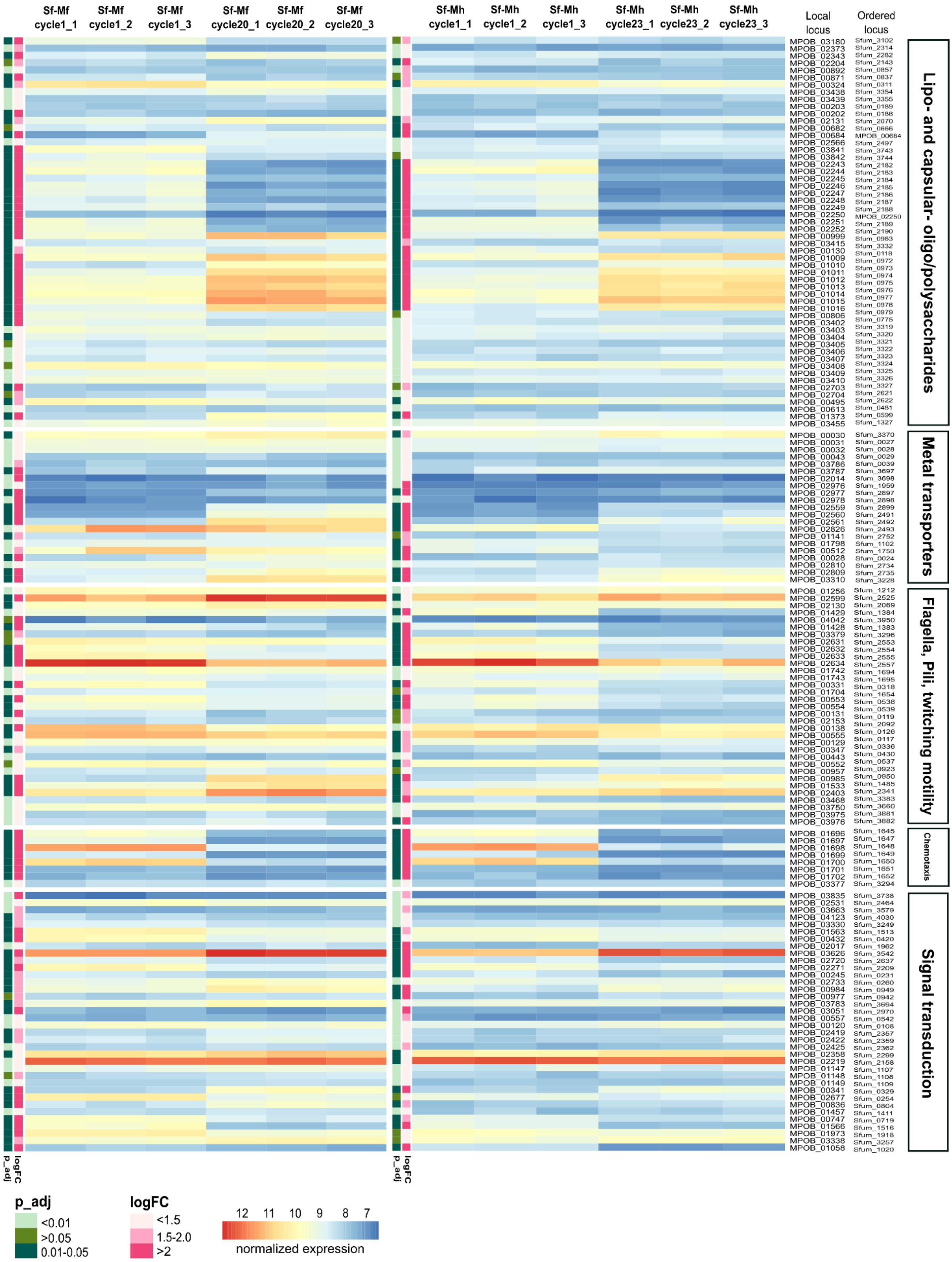

Figure S15. A normalized differential expression of the *S. fumaroxidans* genes for the secondary metabolism in Sf-Mf and Sf-Mh early- (Cycle-1) and late-aggregation (Cycles 20/23) state co-cultures.

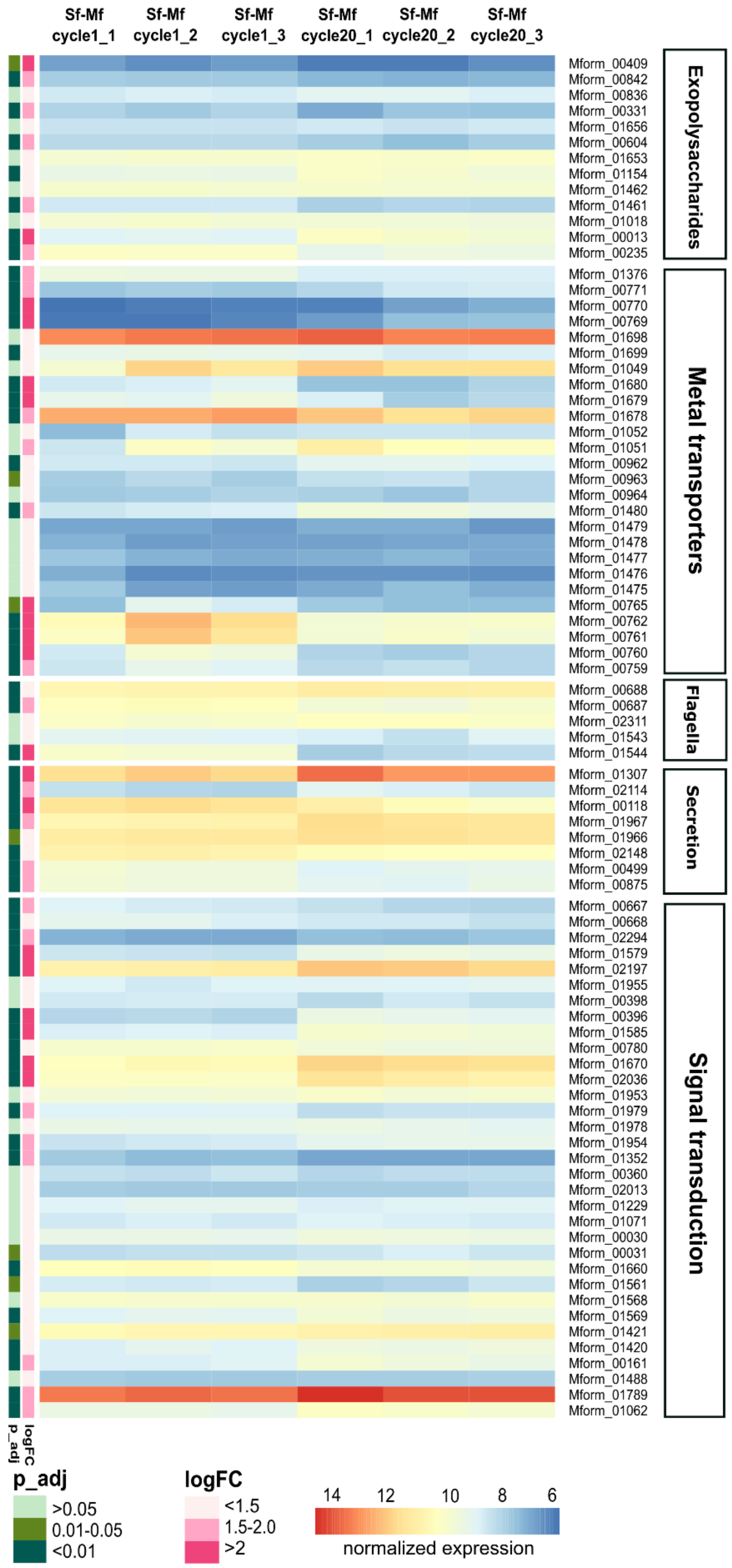

Figure S16. A normalized differential expression of the *M. formicicum* genes for the secondary metabolism in Sf-Mf early- (Cycle-1) and late-aggregation (Cycle-20) state co-cultures.

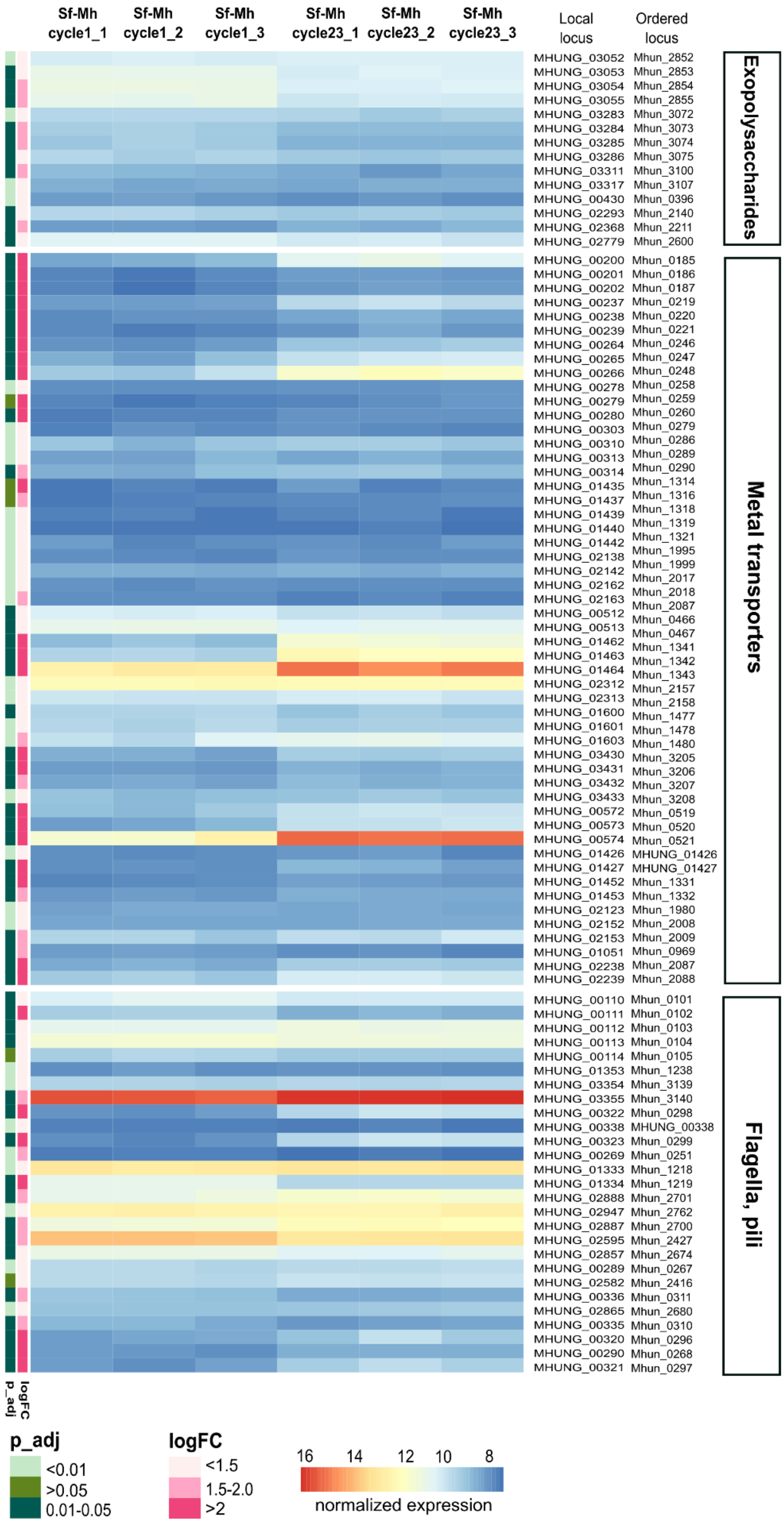

Figure S17. A normalized differential expression of the *M. hungatei* genes for the secondary metabolism (biosynthesis of EPS, metal transporters, regulation of flagella and pili) in Sf-Mh early- (Cycle-1) and late-aggregation (Cycle-23) state co-cultures.

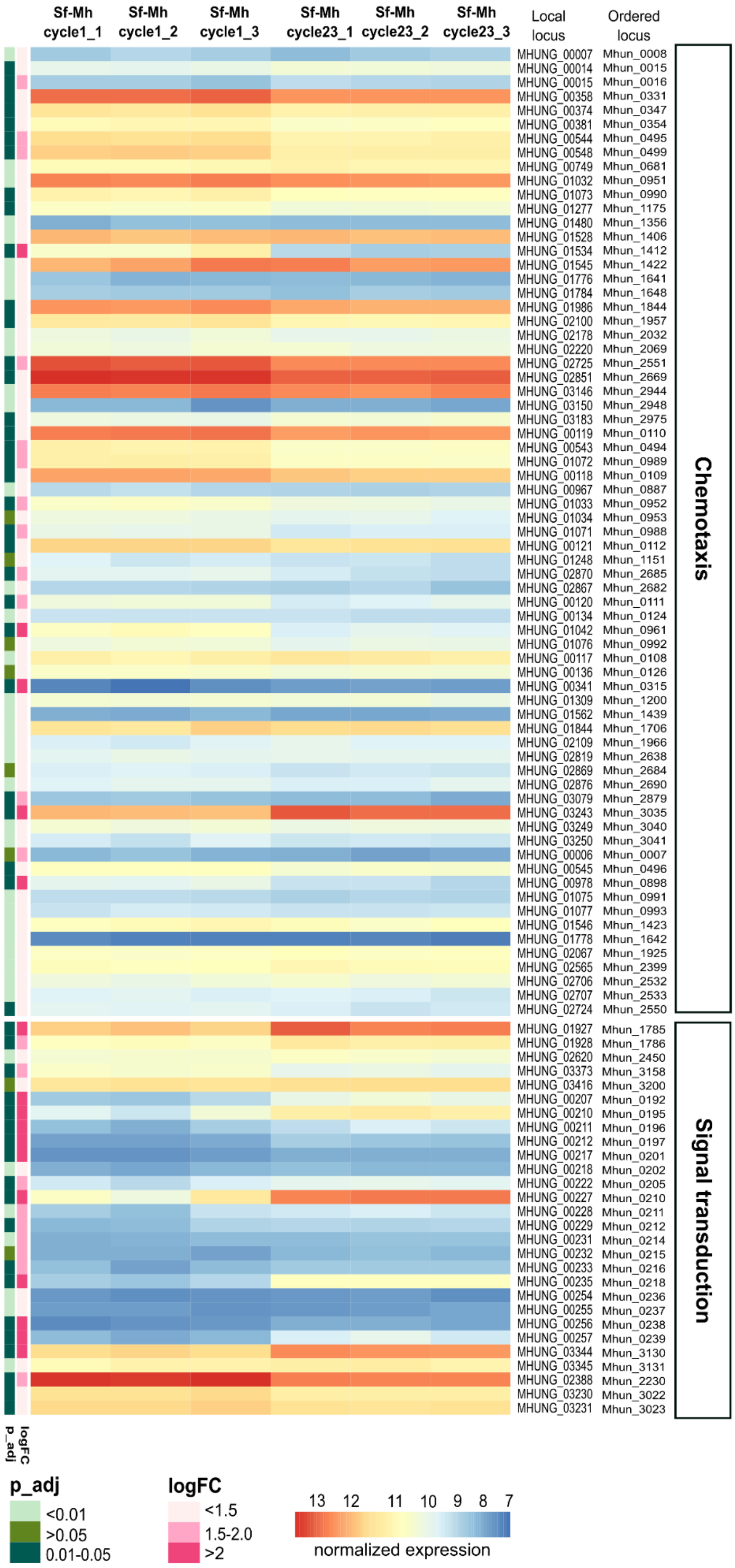

Figure S18. A normalized differential expression of the *M. hungatei* genes for the secondary metabolism (chemotaxis and signal transduction) in Sf-Mh early- (Cycle-1) and late-aggregation (Cycle-23) state co-cultures.

```
---
title: "Transcriptome analysis of 1 year-old aggregates of S.fumaroxidans MPOB with M.hungatei JF-1 or
M.formicicumMF"
author: "Anna Doloman (original script made by Nico Vecchini and Maaike Besteman"
date: "`r strftime(Sys.Date(), '%B %d %Y')`"
output:
  pdf_document: default
  html_document: default
  word_document: default
  always_allow_html: true
---

```{r setup, include=FALSE}
# This chunk contains all the packages and needed to run the differential analysis.

# The code below only applys when your knitting a document in R Markdown. Look up Rmarkdowns for more
information.
knitr::opts_chunk$set(fig.width=12, fig.height=8, echo=FALSE, warning=FALSE, message=FALSE,fig.path='./..
/Figs/')

#First you must install the biocmanager installer. Which is commented below.
#all other packages needed are below.

#biocmagater installer
# if (!requireNamespace("BiocManager", quietly = TRUE))
#     install.packages("BiocManager")
# BiocManager::install(version = "3.12")

#install.packages("tidyverse")
library(tidyverse)
#install.packages("dplyr")
library(dplyr)
#install.packages('svglite')
library(svglite)
#install.packages("janitor")
library(janitor)
#BiocManager::install("DESeq2")
library(DESeq2)
#BiocManager::install("apeglm")
library(apeglm)
#BiocManager::install("Biostrings")
library(Biostrings)
#BiocManager::install("EnhancedVolcano")
library(EnhancedVolcano)
#install.packages("pheatmap")
library(pheatmap)
#install.packages("matrixStats")
#package required for deseq run
```



```{r}
# Loads all the scripts necessary to run analysis
source(here::here("settings.R"))
source(file.path(data.script, "functionsAD.R"))
# load reference gene files
ref_mpob_sequence_genes <- ref.files.genes("MPOB.ffn")
ref_form_sequence_genes <- ref.files.genes("MformWUR.ffn")
ref_hung_sequence_genes <- ref.files.genes("Mhung.ffn")
ref_mpob_sequence <- ref.files("MPOB.ffn", "MPOB.faa")
ref_form_sequence <- ref.files("MformWUR.ffn", "MformWUR.faa")
ref_hung_sequence <- ref.files("Mhung.ffn", "Mhung.faa")
gff_mpob <- gff.org("MPOB.gff")
gff_hung <- gff.org("Mhung.gff")
gff_form <- gff.org("MformWUR.gff")

# # load sequencing data
# # R knows where to find the correct data from the settings.R script
#   # which specifies the location in "data.raw.dir  <- file.path(data.dir, "raw")"
#
source(file.path(data.script, "load_counts_data.R"))
df_counts_mpob <- open.counts.data("MPOB", "MEFAFJCG|NC|PHHPAKOO", ref_mpob_sequence_genes)  ## order is
EPMPDPEG LJADDMKC MEFAFJCG (MIKPEICC) PHHPAKOO
df_counts_hung <- open.counts.data("Mhung", "PHHPAKOO|EPMPDPEG|NC", ref_hung_sequence_genes)
df_counts_form <- open.counts.data("MformWUR", "EPMPDPEG|MEFAFJCG|NC", ref_form_sequence_genes)

# calc transcripts per million
df.tpm.mpob<-tpm_cal(df_counts_mpob,gff_mpob)
df.tpm.form<-tpm_cal(df_counts_form,gff_form)
```
```

````
df.tpm.hung<-tpm_cal(df_counts_hung,gff_hung)
```


```{r TPM analysis Sfum-MPOB in triplicates of cocultures Sf-Mh_cycle1(MMH1) and Sf-Mh_cycle23(MMH23),
eval=T}
# Generate data frames filtering for all genes that are below 50 transcripts per million

#useful to filter out certain genes, its always based on own criteria.
df.show.mpob<-df.tpm.mpob #%>%
  # optional to filter for all tpm above 50 tpm
  #filter(!rowSums(.[-1] <=50 ))


# adding rank allows to understands the level of expression. Its is also often used in RNA-seq analysis
involving one condition.
# Rank is calculated as the mean of each condition.

#add Rank
#now selecting for the MMH cocultures 1 and 23
df.tpm_rank.1<-df.show.mpob %>%
  mutate(MMH1_mean= (MMH1_1+MMH1_2+MMH1_3)/3) %>%
  mutate(MMH23_mean= (MMH23_1+MMH23_2+MMH23_3)/3)

#rank condition MMH1
df.tpm_rank.mpob.MMH1<-df.tpm_rank.1[order(df.tpm_rank.1$MMH1_mean, decreasing = TRUE),]%>%
  distinct(locus_tag, .keep_all = TRUE)%>%
  rownames_to_column(var="Rank") %>%
  mutate(Rank=as.numeric(Rank)) %>%
  dplyr::rename(Rank_MMH1=Rank) %>%
  select(2,1)

#rank Condition MMH23
df.tpm_rank.mpob.MMH23<-df.tpm_rank.1[order(df.tpm_rank.1$MMH23_mean, decreasing = TRUE),]%>%
  distinct(locus_tag, .keep_all = TRUE)%>%
  rownames_to_column(var="Rank") %>%
  mutate(Rank=as.numeric(Rank)) %>%
  dplyr::rename(Rank_MMH23=Rank) %>%
  select(2,1)


# joins the dataframe with Rank
df.tpm_mpob_MMH<- df.show.mpob %>%
  left_join(df.tpm_rank.mpob.MMH1) %>%
  left_join(df.tpm_rank.mpob.MMH23) %>%
  select(1, 8:13, 23,24)

# add to the rank and tpm also all extra information.
df.tpm.analysis.mpob.mmh<- df.tpm_mpob_MMH %>%
  left_join(ref_mpob_sequence,by="locus_tag") %>%
  left_join(gff_mpob,by="locus_tag") #%>%
  #left_join(hmm_ko_wolfei,by="locus_tag") %>%
  #Can use other annotation data
  #left_join(inter_wolfei,by="locus_tag") #%>%
  #left_join(keg.ontho.list,by="ko") #%>%
  #select(c(1:3,6,8,11,14,21:23,39))

# stores rds for later use.
write_rds(df.tpm.analysis.mpob.mmh,file.path(data.tables.dir,"tpm_analysis.mpob.mmh.may1.rds"))


```

```{r TPM analysis of Sfum-MPOB in triplicates of cocultures Sf-Mf_cycle1(MMK1) and Sf-Mf_cycle20(MMK23) }
# Generate data frames filtering for all genes that are below 50 transcripts per million

#useful to filter out certain genes, its always based on own criteria.
df.show.mpob<-df.tpm.mpob #%>%
  # optional to filter for all tpm above 50 tpm
  #filter(!rowSums(.[-1] <=50 ))

# adding rank allows to understands the level of expression. Its is also often used in RNA-seq analysis
involving one condition.
# Rank is calculated as the mean of each condition.

#add Rank
#now selecting for the MMK cocultures 1 and 20
df.tpm_rank.2<-df.show.mpob %>%
  mutate(MMK1_mean= (MMK1_1+MMK1_2+MMK1_3)/3) %>%
  mutate(MMK20_mean= (MMK20_1+MMK20_2+MMK20_3)/3)
````

```
#rank condition MMK1
df.tpm_rank.mpob.MMK1<-df.tpm_rank.2[order(df.tpm_rank.2$MMK1_mean, decreasing = TRUE),]%>%
  distinct(locus_tag, .keep_all = TRUE)%>%
  rownames_to_column(var="Rank") %>%
  mutate(Rank=as.numeric(Rank)) %>%
  dplyr::rename(Rank_MMK1=Rank) %>%
  select(2,1)

#rank Condition MMK20
df.tpm_rank.mpob.MMK20<-df.tpm_rank.2[order(df.tpm_rank.2$MMK20_mean, decreasing = TRUE),]%>%
  distinct(locus_tag, .keep_all = TRUE)%>%
  rownames_to_column(var="Rank") %>%
  mutate(Rank=as.numeric(Rank)) %>%
  dplyr::rename(Rank_MMK20=Rank) %>%
  select(2,1)


# joins the dataframe with Rank
df.tpm_mpob_MMK<- df.show.mpob %>%
  left_join(df.tpm_rank.mpob.MMK1) %>%
  left_join(df.tpm_rank.mpob.MMK20) %>%
  select(1, 14:19, 23,24)

# add to the rank and tpm also all extra information.
df.tpm.analysis.mpob.mmk<- df.tpm_mpob_MMK %>%
  left_join(ref_mpob_sequence,by="locus_tag") %>%
  left_join(gff_mpob,by="locus_tag") #%>%
  #left_join(hmm_ko_wolfei,by="locus_tag") %>%
  #Can use other annotation data
  #left_join(inter_wolfei,by="locus_tag") #%>%
  #left_join(keg.ontho.list,by="ko") #%>%
  #select(c(1:3,6,8,11,14,21:23,39))

# stores rds for later use.
write_rds(df.tpm.analysis.mpob.mmk,file.path(data.tables.dir,"tpm_analysis.mpob.mmk.may1.rds"))
```


```{r TPM analysis Mformicicum in cocultures Sf-Mf_cycle1(MMK1) and Sf-Mf_cycle20(MMK20), eval=T}
# Generate data frames filtering for all genes that are below 50 transcripts per million

#useful to filter out certain genes, its always based on own criteria.
df.show.form<-df.tpm.form #%>%
  # optional to filter for all tpm above 50 tpm
  #filter(!rowSums(.[-1] <=50 ))

# adding rank allows to understands the level of expression. Its is also often used in RNA-seq analysis
involving one condition.
# Rank is calculated as the mean of each condition.

#add Rank
#now selecting for the MMK cocultures 1 and 20
df.tpm_rank.4<-df.show.form %>%
  mutate(MMK1_mean= (MMK1_1+MMK1_2+MMK1_3)/3) %>%
  mutate(MMK20_mean= (MMK20_1+MMK20_2+MMK20_3)/3)

#rank condition MMK1
df.tpm_rank.form.MMK1<-df.tpm_rank.4[order(df.tpm_rank.4$MMK1_mean, decreasing = TRUE),]%>%
  distinct(locus_tag, .keep_all = TRUE)%>%
  rownames_to_column(var="Rank") %>%
  mutate(Rank=as.numeric(Rank)) %>%
  dplyr::rename(Rank_MMK1=Rank) %>%
  select(2,1)

#rank Condition MMK20
df.tpm_rank.form.MMK20<-df.tpm_rank.4[order(df.tpm_rank.4$MMK20_mean, decreasing = TRUE),]%>%
  distinct(locus_tag, .keep_all = TRUE)%>%
  rownames_to_column(var="Rank") %>%
  mutate(Rank=as.numeric(Rank)) %>%
  dplyr::rename(Rank_MMK20=Rank) %>%
  select(2,1)


# joins the dataframe with Rank
df.tpm_form_MMK<- df.show.form %>%
  left_join(df.tpm_rank.form.MMK1) %>%
  left_join(df.tpm_rank.form.MMK20) %>%
  select(1, 14:19,23,24)

# add to the rank and tpm also all extra information.
df.tpm.analysis.form.mmk<- df.tpm_form_MMK %>%
```

````
  left_join(ref_form_sequence,by="locus_tag") %>%
  left_join(gff_form,by="locus_tag") #%>%
  #left_join(hmm_ko_wolfei,by="locus_tag") %>%
  #Can use other annotation data
  #left_join(inter_wolfei,by="locus_tag") #%>%
  #left_join(keg.ontho.list,by="ko") #%>%
  #select(c(1:3,6,8,11,14,21:23,39))

# stores rds for later use.
write_rds(df.tpm.analysis.form.mmk,file.path(data.tables.dir,"tpm_analysis.form.mmk.may1.rds"))

```

```{r TPM analysis of M.hungatei in Sf-Mh_cycle1(MMH1) and Sf-Mh_cycle23(MMH23), eval=T}

#Generate data frames filtering for all genes that are below 50 transcripts per million
df.show.hung<-df.tpm.hung #%>%
  # optional to filter for all tpm above 50 tpm
  #filter(!rowSums(.[-1] <=50 ))

#now selecting for the MMH cocultures 1 and 23
df.tpm_rank.3<-df.show.hung %>%
  mutate(MMH1_mean= (MMH1_1+MMH1_2+MMH1_3)/3) %>%
  mutate(MMH23_mean= (MMH23_1+MMH23_2+MMH23_3)/3)

#rank condition MMH1
df.tpm_rank.hung.MMH1<-df.tpm_rank.3[order(df.tpm_rank.3$MMH1_mean, decreasing = TRUE),]%>%
  distinct(locus_tag, .keep_all = TRUE)%>%
  rownames_to_column(var="Rank") %>%
  mutate(Rank=as.numeric(Rank)) %>%
  dplyr::rename(Rank_MMH1=Rank) %>%
  select(2,1)


#Rank condition T
df.tpm_rank.hung.MMH23<-df.tpm_rank.3[order(df.tpm_rank.3$MMH23_mean, decreasing = TRUE),]%>%
  distinct(locus_tag, .keep_all = TRUE)%>%
  rownames_to_column(var="Rank") %>%
  mutate(Rank=as.numeric(Rank)) %>%
  dplyr::rename(Rank_T=Rank) %>%
  select(2,1)


# joins the dataframe with Rank
df.tpm_hung.mmh <- df.show.hung %>%
  left_join(df.tpm_rank.hung.MMH1) %>%
  left_join(df.tpm_rank.hung.MMH23) %>%
   select(1, 8:13, 23,24)

# add to the rank and tpm also all extra information.
df.tpm.analysis.hung.mmh<- df.tpm_hung.mmh %>%
  left_join(ref_hung_sequence,by="locus_tag") %>%
  left_join(gff_hung,by="locus_tag") #%>%
  #left_join(hmm_ko_hung,by="locus_tag") #%>%
  #left_join(inter_hung,by="locus_tag") #%>%
  #left_join(keg.ontho.list,by="ko") #%>%
  #select(c(1:3,6,8,11,14,21:23,39))

# stores rds for later use.
write_rds(df.tpm.analysis.hung.mmh,file.path(data.tables.dir,"tpm_analysis.hung.mmh.rds"))


```
#Study 1 Differential expression analysis: cocultures of Syntrophobacter fumaroxidans MPOB and
Methanospirillum hungatei JF-1


```{r DEseq for Sfum-MPOB in triplicates of cocultures Sf-Mh_cycle1(MMH1) and Sf-Mh_cycle23(MMH23) data}
#Look at DSEQ guide for how to use this commands http://bioconductor.org/packages/release/bioc/vignettes
/DESeq2/inst/doc/DESeq2.html

#global condition
condition_mpob_mmh<-as.factor(c("null","MMH1","MMH1","MMH1","MMH23","MMH23","MMH23"))
file_mpob_mmh<- colnames(df_counts_mpob)


rep_condt_mpob_mmh<-data_frame(file_mpob_mmh[c(8:13)],condition_mpob_mmh[c(-1)])
colnames(rep_condt_mpob_mmh)<-c("replicates","conditions")


df.counts.mpob.1<-df_counts_mpob %>% remove_rownames %>%
````

````
column_to_rownames(var="locus_tag")

df.counts.mpob.1[is.na(df.counts.mpob.1)] <- 0


##DESeq is coming now
dds_mpob_mmh <- DESeqDataSetFromMatrix(countData = df.counts.mpob.1[,c(7:12)] ,
                              colData = rep_condt_mpob_mmh,
                              design= ~ conditions)
dds_mpob_mmh$conditions <- relevel(dds_mpob_mmh$conditions, ref = "MMH1")

dds_mpob_mmh <- DESeq(dds_mpob_mmh)
resultsNames(dds_mpob_mmh)

###presumably an input for the heatmap (variant stabilized data for the normalized gene expression differences
between the two conditions,
##in this case between MMH1 and MMH23 triplicates)

#Plot PCA
 vsd_mpob_mmh <- vst(dds_mpob_mmh, blind=FALSE)

#tiff("PCA_MMH1vsMMH23_mpob.tiff", units="in", width=10, height=3, res=300)
 plotPCA(vsd_mpob_mmh, intgroup=c("conditions"))
#dev.off()


#--- Continue the DEseq normaized output ---
norm.counts<- counts(dds_mpob_mmh,normalized=TRUE)
df.norm.counts.mpob.mmh<-data.frame(norm.counts)

write.csv(df.norm.counts.mpob.mmh, file ="DEseq_normCounts_mpob_inMMH_flocs.may1.csv", row.names = TRUE)

```


```{r Sfum-MPOB in Sf-Mh_cycle1 VS Sfum-MPOB in Sf-Mh_cycle23}

# TPMs are used here now, calculated earlier


# this a function to obtain the results as a dataframe.
results_MMH1vsMMH23_mpob <- results(dds_mpob_mmh, contrast=c("conditions","MMH1","MMH23"))
#rename the rownames to have a more meaningful name on the Volcanoplot. EPMPDPEG to MPOB
#row.names(results_MMH1vsMMH23_mpob) <- gsub("EPMPDPEG", "MPOB", row.names(results_MMH1vsMMH23_mpob))


#create a volcano plot of the results
#tiff("DEseq_MMH1vsMMH23_mpob.tiff", units="in", width=15, height=15, res=300)
image1=EnhancedVolcano(results_MMH1vsMMH23_mpob,
    lab = rownames(results_MMH1vsMMH23_mpob),
    x = 'log2FoldChange',
    y = 'padj',
    FCcutoff = 1,
    legendPosition = "right")
ggsave("DEseq_MMH1vsMMH23_mpob.svg", plot = image1, width=15, height=15)
#dev.off()

#rename back the locus tags to allow mapping with the previous output in tpm analysis
row.names(results_MMH1vsMMH23_mpob) <- gsub("MPOB", "EPMPDPEG", row.names(results_MMH1vsMMH23_mpob))

#convert the locus_tags from the rows into a column of there own.
df.res.MMH1vsMMH23.mpob<- data.frame(results_MMH1vsMMH23_mpob) %>%
  rownames_to_column(var="locus_tag")



df.res.MMH1vsMMH23.mpob.exp.all<-df.res.MMH1vsMMH23.mpob%>%
  #left_join(df.norm.counts.ace.1,by="locus_tag") %>%
  left_join(df.tpm.analysis.mpob.mmh,by="locus_tag") %>%
  distinct(locus_tag,.keep_all=TRUE) #%>%
   #select(-8,-10,-12:-14,-18,-20,-24:-29)

#store the rds for later use.
write_rds(df.res.MMH1vsMMH23.mpob.exp.all,file.path(data.tables.dir,"Differential_analysis.mpob.mmh.may1.rds"))

write.csv(df.res.MMH1vsMMH23.mpob.exp.all, file ="DEseq_all_mpob_inMMH_flocs.may1.csv", row.names = FALSE)
```
````
````

```
``` {r Heatmaps Sfum-MPOB in Sf-Mh_cycle1 VS Sfum-MPOB in Sf-Mh_cycle23}

#Create a heatmap usable data from normalized vsd_* expression from DEseq
#Make the locus_tags the rownames
##the command left_join below used "all.x=TRUE" before 2023 R version. Now it is deprecated, and you do not
need to pass that argument any more.
mpob_mmh_heatmap_data <- data.frame(assay(vsd_mpob_mmh)) %>%
  rownames_to_column(var="locus_tag")  %>%
  left_join(df.res.MMH1vsMMH23.mpob.exp.all[,c("locus_tag", "baseMean", "log2FoldChange", "padj", "gp_name")],
by="locus_tag") %>%
  #Make the locus_tags the rownames
 'rownames<-'(.$locus_tag) %>%
  select(-"locus_tag") %>%
  mutate_at(c(1:9), as.numeric) %>%
  #Make categories for log2foldchange and adjusted p-values to include in heatmaps
  mutate(logFC = case_when(log2FoldChange < 0.585 & log2FoldChange >= -0.585 ~ '<1.5',
                           log2FoldChange < 1.0  & log2FoldChange >= 0.585 ~ '1.5-2.0',
                           log2FoldChange > -1.0  & log2FoldChange <= -0.585 ~ '1.5-2.0',
                           log2FoldChange >= 1.0 ~ '>2',
                           log2FoldChange <= -1.0 ~ '>2')) %>%
  mutate(p_adj = case_when(padj >= 0.05  ~ '>0.05',
                            padj < 0.05 & padj >= 0.01 ~ '0.01-0.05',
                            padj < 0.01 ~ '<0.01'))

row.names(mpob_mmh_heatmap_data) <- gsub("EPMPDPEG", "MPOB", row.names(mpob_mmh_heatmap_data))

#making a colour palette for the LogFC and p_adj to be used in th heatmap
ann_colors <- list(logFC=c('<1.5'="#fff0f0", '1.5-2.0'="#ffa5c6",'>2'="#ef447b"),
                   p_adj=c('>0.05'="#c5e8c6", '0.01-0.05'="#60851f", '<0.01'="#015a51"))



#### Central metabolism genes of Sfumaroxidans (propionate oxidation, hydrogenases, formate dehydrogenases)

mpob.central <- c("MPOB_04019", "MPOB_04020", "MPOB_04021", "MPOB_04022", "MPOB_04023", "MPOB_04024",
"MPOB_04025", "MPOB_04026",
                  "MPOB_00467", "MPOB_00468", "MPOB_00469", "MPOB_00470", "MPOB_01750", "MPOB_01751",
"MPOB_02057", "MPOB_02058",
                  "MPOB_02059", "MPOB_04186", "MPOB_04187", "MPOB_04188", "MPOB_04189", "MPOB_00186",
"MPOB_00187", "MPOB_02164",
                  "MPOB_02165", "MPOB_02162", "MPOB_02163", "MPOB_02395", "MPOB_00473", "MPOB_00474",
"MPOB_01267", "MPOB_02868",
                  "MPOB_02869", "MPOB_02870", "MPOB_02871", "MPOB_00399", "MPOB_00400", "MPOB_00775",
"MPOB_01322", "MPOB_03151",
                  "MPOB_00843", "MPOB_00844", "MPOB_00845", "MPOB_01176", "MPOB_01177", "MPOB_01178",

                  "MPOB_00878", "MPOB_00879", "MPOB_00880", "MPOB_01840", "MPOB_01841", "MPOB_01843",
"MPOB_01844", "MPOB_02285",
                  "MPOB_02286", "MPOB_02287", "MPOB_02288", "MPOB_02788", "MPOB_02789", "MPOB_02790",
"MPOB_02791", "MPOB_02792",
                  "MPOB_03618", "MPOB_03619", "MPOB_03620", "MPOB_04046", "MPOB_04047", "MPOB_04049",
"MPOB_04048", "MPOB_00881",
                  "MPOB_00882", "MPOB_03032", "MPOB_03033",

                  "MPOB_01845", "MPOB_01846", "MPOB_01847", "MPOB_01851", "MPOB_01852", "MPOB_01854",
"MPOB_01855", "MPOB_01856",
                  "MPOB_01857", "MPOB_01858", "MPOB_02779", "MPOB_02780", "MPOB_02781", "MPOB_02782",
"MPOB_00033", "MPOB_00034",
                  "MPOB_00039", "MPOB_00040", "MPOB_00041", "MPOB_01318", "MPOB_01319", "MPOB_01320",
"MPOB_03593", "MPOB_03594",
                  "MPOB_02783")

#match the list of interesting genes in the order given in vector to select these genes from the vsd of the
DEseq
mpob.central.mmh.ordered <- mpob_mmh_heatmap_data[mpob.central,]

# for selecting rownames of the future heatmap to have other values than only your locus_tags (current
rowname)
mpob.central.mmh.rownames <- mpob.central.mmh.ordered %>%
  rownames_to_column(., var = "locus_tag") %>%
  select(c("locus_tag")) %>%
  unite(., col='rownames', c("locus_tag"))

#print heatmap in screen
heat_central_mpob_mmh <- pheatmap(mpob.central.mmh.ordered[,1:6], cluster_rows=FALSE, show_rownames=TRUE,
         cluster_cols=FALSE,
         main = "Expression of genes from central metabolism of S. fumaroxidans in Sf-Mh",
         labels_row = mpob.central.mmh.rownames[,1])#,
         #annotation_row adds another column with e.g. calculated LFC or p-values
         #annotation_row = mpob.prop.ox.mmh[,c("log2FoldChange", "padj")])
```
```

```
#save heatmap as png
png("20230526_central_MPOB_inMMH.png", width = 3000, height = 5200, res = 250)
pheatmap(mpob.central.mmh.ordered[,1:6], cluster_rows=FALSE, show_rownames=TRUE,
         cluster_cols=FALSE,
         main = "Expression of genes from central metabolism of S. fumaroxidans in Sf-Mh",
         labels_row = mpob.central.mmh.rownames[,1],
         gaps_row = c(46, 73),
         #annotation_row adds another column with e.g. calculated LFC or p-values)
         annotation_row = mpob.central.mmh.ordered[,c("logFC", "p_adj")],
         annotation_colors = ann_colors,
         border_color = NA
         )
dev.off()

###or save the heatmap as svg
imageHeatmap.MPOB.MMH.central = pheatmap(mpob.central.mmh.ordered[,1:6], cluster_rows=FALSE,
show_rownames=TRUE,
         cluster_cols=FALSE,
         main = "Expression of genes from central metabolism of S. fumaroxidans in Sf-Mh",
         labels_row = mpob.central.mmh.rownames[,1],
         gaps_row = c(46, 73),
         #annotation_row adds another column with e.g. calculated LFC or p-values)
         annotation_row = mpob.central.mmh.ordered[,c("logFC", "p_adj")],
         annotation_colors = ann_colors,
         border_color = NA
         )
ggsave("Heatmap_MPOB_MMH_central.svg", plot = imageHeatmap.MPOB.MMH.central, width=15, height=15)

##Secondary metabolism genes in Sfumaroxidans MPOB (EPS, flagella, pili, chemotaxis, metal transporters,
signal tranduction)
mpob.second <- c("MPOB_03180", "MPOB_02373", "MPOB_02343", "MPOB_02204", "MPOB_00892", "MPOB_00871",
"MPOB_00324", "MPOB_03438", "MPOB_03439",
                 "MPOB_00203", "MPOB_00202", "MPOB_02131", "MPOB_00682", "MPOB_00684", "MPOB_02566",
"MPOB_03841", "MPOB_03842",
                 "MPOB_02243", "MPOB_02244", "MPOB_02245", "MPOB_02246", "MPOB_02247", "MPOB_02248",
"MPOB_02249", "MPOB_02250", "MPOB_02251", "MPOB_02252", "MPOB_00999", "MPOB_03415", "MPOB_00130",
"MPOB_01009",
                 "MPOB_01010", "MPOB_01011", "MPOB_01012", "MPOB_01013", "MPOB_01014", "MPOB_01015",
"MPOB_01016", "MPOB_00806",
                 "MPOB_03402", "MPOB_03403", "MPOB_03404", "MPOB_03405", "MPOB_03406", "MPOB_03407",
"MPOB_03408", "MPOB_03409", "MPOB_03410",
                 "MPOB_02703", "MPOB_02704", "MPOB_00495", "MPOB_00613", "MPOB_01373", "MPOB_03455",

                 "MPOB_00030", "MPOB_00031", "MPOB_00032", "MPOB_00043", "MPOB_03786", "MPOB_03787",
"MPOB_02014", "MPOB_02976",
                 "MPOB_02977", "MPOB_02978", "MPOB_02559", "MPOB_02560", "MPOB_02561", "MPOB_02826",
"MPOB_01141", "MPOB_01798",
                 "MPOB_00512", "MPOB_00028", "MPOB_02810", "MPOB_02809", "MPOB_03310",

                 "MPOB_01256", "MPOB_02599", "MPOB_02130", "MPOB_01429", "MPOB_04042", "MPOB_01428",
"MPOB_03379", "MPOB_02631",
                 "MPOB_02632", "MPOB_02633", "MPOB_02634", "MPOB_01742", "MPOB_01743", "MPOB_00331",
"MPOB_01704", "MPOB_00553",
                 "MPOB_00554", "MPOB_00131", "MPOB_02153", "MPOB_00138", "MPOB_00555", "MPOB_00129",
"MPOB_00347", "MPOB_00443",
                 "MPOB_00552", "MPOB_00957", "MPOB_00985", "MPOB_01533", "MPOB_02403", "MPOB_03468",
"MPOB_03750", "MPOB_03975",
                 "MPOB_03976",

                 "MPOB_01696", "MPOB_01697", "MPOB_01698", "MPOB_01699", "MPOB_01700", "MPOB_01701",
"MPOB_01702","MPOB_03377",

                 "MPOB_03835","MPOB_02531", "MPOB_03663", "MPOB_04123", "MPOB_03330", "MPOB_01563",
"MPOB_00432", "MPOB_02017",
                 "MPOB_03626", "MPOB_02720", "MPOB_02271", "MPOB_00245", "MPOB_02733", "MPOB_00984",
"MPOB_00977", "MPOB_03783",
                 "MPOB_03051", "MPOB_00557", "MPOB_00120",  "MPOB_02419", "MPOB_02422", "MPOB_02425",
"MPOB_02358", "MPOB_02219",
                 "MPOB_01147", "MPOB_01148", "MPOB_01149", "MPOB_00341", "MPOB_02677", "MPOB_00836",
"MPOB_01457", "MPOB_00747",
                 "MPOB_01566", "MPOB_01973", "MPOB_03338", "MPOB_01058")

#match the list of interesting genes in the order given in vector to select these genes from the vsd of the
DEseq
mpob.second.mmh.ordered <- mpob_mmh_heatmap_data[mpob.second,]

# for selecting rownames of the future heatmap to have other values than only your locus_tags (current
rowname)
mpob.second.mmh.rownames <- mpob.second.mmh.ordered %>%
  rownames_to_column(., var = "locus_tag") %>%
  select(c("locus_tag")) %>%
```

````
  unite(., col='rownames', c("locus_tag"))

#print heatmap in screen
heat_second_mpob_mmh <- pheatmap(mpob.second.mmh.ordered[,1:6], cluster_rows=FALSE, show_rownames=TRUE,
         cluster_cols=FALSE,
         main = "Expression of genes from secondary metabolism of S. fumaroxidans in Sf-Mh",
         labels_row = mpob.second.mmh.rownames[,1])#,
         #annotation_row adds another column with e.g. calculated LFC or p-values
         #annotation_row = mpob.prop.ox.mmh[,c("log2FoldChange", "padj")])

#save heatmap as png
png("20230530_secondary_MPOB_inMMH.png", width = 3000, height = 5200, res = 250)
pheatmap(mpob.second.mmh.ordered[,1:6], cluster_rows=FALSE, show_rownames=TRUE,
         cluster_cols=FALSE,
         main = "Expression of genes from secondary metabolism of S. fumaroxidans in Sf-Mh",
         labels_row = mpob.second.mmh.rownames[,1],
         gaps_row = c(54, 75, 108, 116),
         #annotation_row adds another column with e.g. calculated LFC or p-values)
         annotation_row = mpob.second.mmh.ordered[,c("logFC", "p_adj")],
         annotation_colors = ann_colors,
         border_color = NA
         )
dev.off()

###or save the heatmap as svg
imageHeatmap.MPOB.MMH.second = pheatmap(mpob.second.mmh.ordered[,1:6], cluster_rows=FALSE, show_rownames=TRUE,
         cluster_cols=FALSE,
         main = "Expression of genes from secondary metabolism of S. fumaroxidans in Sf-Mh",
         labels_row = mpob.second.mmh.rownames[,1],
         gaps_row = c(54, 75, 108, 116),
         #annotation_row adds another column with e.g. calculated LFC or p-values)
         annotation_row = mpob.second.mmh.ordered[,c("logFC", "p_adj")],
         annotation_colors = ann_colors,
         border_color = NA
         )
ggsave("Heatmap_MPOB_MMH_secondary.svg", plot = imageHeatmap.MPOB.MMH.second, width=15, height=15)

```



```{r Dseq Mhung data for Sf-Mh_cycle1 and Sf-Mh_cycle23 sets}

#put together

#global condition
condition_hung_mmh<-as.factor(c("null","MMH1","MMH1","MMH1","MMH23","MMH23","MMH23"))
file_hung_mmh<- colnames(df_counts_hung)


rep_condi_hung_mmh<-data_frame(file_hung_mmh[c(8:13)],condition_hung_mmh[c(-1)])
#rep_condi_hung<-data_frame(file_hung[-1],condition_hung[-1])
colnames(rep_condi_hung_mmh)<-c("replicates","conditions")

#df.counts.hung<-df.counts.amp.1[c(1,8:10,15:20)] %>% remove_rownames %>%
#df.counts.hung<-df.counts.amp[c(-3:-5,-11:-13,-21)] %>% remove_rownames %>%
df.counts.hung.3<-df_counts_hung %>% remove_rownames %>%
column_to_rownames(var="locus_tag")
#df.counts.hung.1<-df.counts.amp.1[] %>% remove_rownames %>% column_to_rownames(var="locus_tag")
df.counts.hung.3[is.na(df.counts.hung.3)] <- 0

dds_hung_mmh <- DESeqDataSetFromMatrix(countData = df.counts.hung.3[,c(7:12)],
                              colData = rep_condi_hung_mmh,
                              design= ~ conditions)
dds_hung_mmh$conditions <- relevel(dds_hung_mmh$conditions, ref = "MMH1")

dds_hung_mmh <- DESeq(dds_hung_mmh)
resultsNames(dds_hung_mmh)

#Plot PCA
 vsd_hung_mmh <- vst(dds_hung_mmh, blind=FALSE)
# # varianceStabilizingTransformation(dds_hung, blind=FALSE)
 plotPCA(vsd_hung_mmh, intgroup=c("conditions"))
# plotPCA(vsd_hung, intgroup=c("conditions"))

#Percentage of Normalized counts
norm.counts<- counts(dds_hung_mmh,normalized=TRUE)
df.norm.counts.hung.mmh<-data.frame(norm.counts)

write.csv(df.norm.counts.hung.mmh, file ="DEseq_normCounts_mhung_inMMH_flocs.csv", row.names = TRUE)
````

````
```




```{r Mhung data for Sf-Mh_cycle1 and Sf-Mh_cycle23 sets}


results_MMH1vsMMH23_hung <- results(dds_hung_mmh, contrast=c("conditions","MMH1","MMH23"))

#renaming the row names for the nice Volcano Plot
row.names(results_MMH1vsMMH23_hung) <- gsub("MEFAFJCG", "MHUNG", row.names(results_MMH1vsMMH23_hung))


#tiff("DEseq_MMH1vsMMH23_hung.tiff", units="in", width=15, height=15, res=300)
image2=EnhancedVolcano(results_MMH1vsMMH23_hung,
    lab = rownames(results_MMH1vsMMH23_hung),
    x = 'log2FoldChange',
    y = 'padj',
    FCcutoff = 1,
    legendPosition = "right")
ggsave("DEseq_MMH1vsMMH23_hung.svg", plot = image2, width=15, height=15)
#dev.off()

#renaming the row names back to match the TPM data
row.names(results_MMH1vsMMH23_hung) <- gsub("MHUNG", "MEFAFJCG", row.names(results_MMH1vsMMH23_hung))

df.res.MMH1vsMMH23.hung<- data.frame(results_MMH1vsMMH23_hung) %>%
  rownames_to_column(var="locus_tag")


df.res.MMH1vsMMH23.hung.exp.all<-df.res.MMH1vsMMH23.hung%>%
  #left_join(df.norm.counts.ace.1,by="locus_tag") %>%
  left_join(df.tpm.analysis.hung.mmh,by="locus_tag")#%>%
  #distinct(locus_tag,.keep_all=T)

write_rds(df.res.MMH1vsMMH23.hung.exp.all,file.path(data.tables.dir,"Differential_analysis.hung.mmh.rds"))

write.csv(df.res.MMH1vsMMH23.hung.exp.all, file ="DEseq_all_hung_inMMH_flocs.csv", row.names = FALSE)

```



```{r Heatmap Mhung in Sf-Mh_cycle1 VS Mhung in Sf-Mh_cycle23}
#Create a heatmap usable data from normalized vsd_* expression from DEseq
#Make the locus_tags the rownames
##the command left_join below used "all.x=TRUE" before 2023 R version. Now it is deprecated, and you do not
need to pass that argument any more.
mhung_mmh_heatmap_data <- data.frame(assay(vsd_hung_mmh)) %>%
  rownames_to_column(var="locus_tag")  %>%
  left_join(df.res.MMH1vsMMH23.hung.exp.all[,c("locus_tag", "baseMean", "log2FoldChange", "padj", "gp_name")],
by="locus_tag") %>%
  #Make the locus_tags the rownames
 'rownames<-'(.$locus_tag) %>%
  select(-"locus_tag") %>%
  mutate_at(c(1:9), as.numeric) %>%
  #Make categories for log2foldchange and adjusted p-values to include in heatmaps
  mutate(logFC = case_when(log2FoldChange < 0.585 & log2FoldChange >= -0.585 ~ '<1.5',
                           log2FoldChange < 1.0  & log2FoldChange >= 0.585 ~ '1.5-2.0',
                           log2FoldChange > -1.0  & log2FoldChange <= -0.585 ~ '1.5-2.0',
                           log2FoldChange >= 1.0 ~ '>2',
                           log2FoldChange <= -1.0 ~ '>2')) %>%
  mutate(p_adj = case_when(padj >= 0.05  ~ '>0.05',
                            padj < 0.05 & padj >= 0.01 ~ '0.01-0.05',
                            padj < 0.01 ~ '<0.01'))

row.names(mhung_mmh_heatmap_data) <- gsub("MEFAFJCG", "MHUNG", row.names(mhung_mmh_heatmap_data))

#making a colour palette for the LogFC and p_adj to be used in th heatmap
ann_colors <- list(logFC=c('<1.5'="#fff0f0", '1.5-2.0'="#ffa5c6",'>2'="#ef447b"),
                   p_adj=c('>0.05'="#c5e8c6", '0.01-0.05'="#60851f", '<0.01'="#015a51"))


#####central metabolism of M. hungatei JF1 (hydrogenotrophic methanogenesis, hydrogenases, formate
dehydrogenases )
mhung.central <- c("MHUNG_02124", "MHUNG_02125", "MHUNG_02126", "MHUNG_02127", "MHUNG_02128",
"MHUNG_02130","MHUNG_02131", "MHUNG_02132", "MHUNG_02133",
                   "MHUNG_02134", "MHUNG_02135", "MHUNG_02136", "MHUNG_02137", "MHUNG_02258", "MHUNG_02259",
"MHUNG_02260", "MHUNG_02261", "MHUNG_02262",
                   "MHUNG_02263", "MHUNG_02264", "MHUNG_00021", "MHUNG_00487", "MHUNG_02552", "MHUNG_01949",
````

```
"MHUNG_02413", "MHUNG_02415", "MHUNG_02323",
                   "MHUNG_02324", "MHUNG_02325", "MHUNG_02326", "MHUNG_02328", "MHUNG_02329", "MHUNG_02330",
"MHUNG_02297", "MHUNG_02298", "MHUNG_02299",
                   "MHUNG_02300", "MHUNG_02301", "MHUNG_01975", "MHUNG_01976", "MHUNG_01977", "MHUNG_01978",
"MHUNG_01979", "MHUNG_01980",

                   "MHUNG_01880", "MHUNG_01881", "MHUNG_01882", "MHUNG_01883", "MHUNG_01884", "MHUNG_01885",
"MHUNG_01886", "MHUNG_01958", "MHUNG_01959",
                   "MHUNG_01960", "MHUNG_01961", "MHUNG_01962", "MHUNG_01963", "MHUNG_02245", "MHUNG_02246",
"MHUNG_02247", "MHUNG_02248", "MHUNG_02249",
                   "MHUNG_02250", "MHUNG_02251", "MHUNG_02253", "MHUNG_02254", "MHUNG_02255", "MHUNG_02256",
"MHUNG_02257", "MHUNG_02758", "MHUNG_02759",
                   "MHUNG_02760", "MHUNG_02761", "MHUNG_02762", "MHUNG_02763", "MHUNG_02764", "MHUNG_02765",
"MHUNG_02766", "MHUNG_02767", "MHUNG_02768",
                   "MHUNG_02769", "MHUNG_02770", "MHUNG_02771", "MHUNG_02492", "MHUNG_02493", "MHUNG_02494",
"MHUNG_02495",

                   "MHUNG_01954", "MHUNG_01955", "MHUNG_01973", "MHUNG_01974", "MHUNG_02165", "MHUNG_02166",
"MHUNG_02167", "MHUNG_02168", "MHUNG_03463",
                   "MHUNG_03464",

                   "MHUNG_00701", "MHUNG_00379", "MHUNG_00622", "MHUNG_01860", "MHUNG_00653", "MHUNG_02559",
"MHUNG_02560", "MHUNG_02561", "MHUNG_02562",
                   "MHUNG_02563", "MHUNG_00493", "MHUNG_00494", "MHUNG_00495", "MHUNG_00496", "MHUNG_03404",
"MHUNG_03405", "MHUNG_02315", "MHUNG_01237",
                   "MHUNG_00188",

                  "MHUNG_02432")

#match the list of interesting genes in the order given in vector to select these genes from the vsd of the
DEseq
mhung.central.mmh.ordered <- mhung_mmh_heatmap_data[mhung.central,]

# for selecting rownames of the future heatmap to have other values than only your locus_tags (current
rowname)mhung.hyd.mmh.ordered$gp_name[row.names(mhung.hyd.mmh.ordered)=="MHUNG_02758"] <- " Coenzyme F420
hydrogenase subunit alpha"
mhung.central.mmh.rownames <- mhung.central.mmh.ordered %>%
  rownames_to_column(., var = "locus_tag") %>%
  select(c("locus_tag")) %>%
  unite(., col='rownames', c("locus_tag"))

#print heatmap in screen
heat_central_mhung_mmh <- pheatmap(mhung.central.mmh.ordered[,1:6], cluster_rows=FALSE, show_rownames=TRUE,
         cluster_cols=FALSE,
         main = "Expression of genes from central metabolism of M. hungatei in Sf-Mh",
         labels_row = mhung.central.mmh.rownames[,1])#,
         #annotation_row adds another column with e.g. calculated LFC or p-values
         #annotation_row = mpob.prop.ox.mmh[,c("log2FoldChange", "padj")])

#save heatmap as png
png("20230531_Central_Mhung_inMMH.png", width = 3000, height = 5200, res = 250)
pheatmap(mhung.central.mmh.ordered[,1:6], cluster_rows=FALSE, show_rownames=TRUE,
         #cellheight = 10,
         #cellwidth = 35,
         cluster_cols=FALSE,
         main = "Expression of genes from central metabolism of M. hungatei in Sf-Mh",
         labels_row = mhung.central.mmh.rownames[,1],
         gaps_row = c(44, 87, 97),
         #annotation_row adds another column with e.g. calculated LFC or p-values)
         annotation_row = mhung.central.mmh.ordered[,c("logFC", "p_adj")],
         annotation_colors = ann_colors,
         border_color = NA
         )
dev.off()


###or save the heatmap as svg
imageHeatmap.Mhung.MMH.central = pheatmap(mhung.central.mmh.ordered[,1:6], cluster_rows=FALSE,
show_rownames=TRUE,
         cluster_cols=FALSE,
         main = "Expression of genes from central metabolism of M. hungatei in Sf-Mh",
         labels_row = mhung.central.mmh.rownames[,1],
         gaps_row = c(44, 87, 97),
         #annotation_row adds another column with e.g. calculated LFC or p-values)
         annotation_row = mhung.central.mmh.ordered[,c("logFC", "p_adj")],
         annotation_colors = ann_colors,
         border_color = NA)
ggsave("Central.Mhung.MMH_0531.svg", plot = imageHeatmap.Mhung.MMH.central, width=15, height=15)

###Secondary metabolism M. hungatei JF1 (EPS, flagella, pili, chemotaxism metal transporters, signal
transduction)
```

```
mhung.second1 <- c("MHUNG_03052", "MHUNG_03053", "MHUNG_03054", "MHUNG_03055", "MHUNG_03283", "MHUNG_03284",
"MHUNG_03285", "MHUNG_03286", "MHUNG_03311",
                   "MHUNG_03317", "MHUNG_00430", "MHUNG_02293", "MHUNG_02368",

                  "MHUNG_00200", "MHUNG_00201", "MHUNG_00202", "MHUNG_00237", "MHUNG_00238", "MHUNG_00239",
"MHUNG_00264", "MHUNG_00265", "MHUNG_00266",
                  "MHUNG_00278", "MHUNG_00279", "MHUNG_00280", "MHUNG_00303", "MHUNG_00310", "MHUNG_00313",
"MHUNG_00314", "MHUNG_01435", "MHUNG_01437",
                  "MHUNG_01439", "MHUNG_01440", "MHUNG_01442", "MHUNG_02138", "MHUNG_02142", "MHUNG_02162",
"MHUNG_02163", "MHUNG_00512", "MHUNG_00513",
                  "MHUNG_01462", "MHUNG_01463", "MHUNG_01464", "MHUNG_02312", "MHUNG_02313", "MHUNG_01600",
"MHUNG_01601", "MHUNG_01603", "MHUNG_03430",
                  "MHUNG_03431", "MHUNG_03432", "MHUNG_03433", "MHUNG_00572", "MHUNG_00573", "MHUNG_00574",
"MHUNG_01426", "MHUNG_01427", "MHUNG_01452",
                  "MHUNG_01453", "MHUNG_02123", "MHUNG_02152", "MHUNG_02153", "MHUNG_01051", "MHUNG_02238",
"MHUNG_02239",

                  "MHUNG_00110", "MHUNG_00111", "MHUNG_00112", "MHUNG_00113", "MHUNG_00114", "MHUNG_01353",
"MHUNG_03354", "MHUNG_03355", "MHUNG_00322",
                  "MHUNG_00338", "MHUNG_00323", "MHUNG_00269", "MHUNG_01333", "MHUNG_01334", "MHUNG_02888",
"MHUNG_02947", "MHUNG_02887", "MHUNG_02595",
                  "MHUNG_02857", "MHUNG_00289", "MHUNG_02582", "MHUNG_00336", "MHUNG_02865", "MHUNG_00335",
"MHUNG_00320", "MHUNG_00290", "MHUNG_00321",
                  "MHUNG_02779")

mhung.second2 <- c("MHUNG_00007", "MHUNG_00014", "MHUNG_00015", "MHUNG_00358", "MHUNG_00374", "MHUNG_00381",
"MHUNG_00544", "MHUNG_00548", "MHUNG_00749",
                  "MHUNG_01032", "MHUNG_01073", "MHUNG_01277", "MHUNG_01480", "MHUNG_01528", "MHUNG_01534",
"MHUNG_01545", "MHUNG_01776", "MHUNG_01784",
                  "MHUNG_01986", "MHUNG_02100", "MHUNG_02178", "MHUNG_02220", "MHUNG_02725", "MHUNG_02851",
"MHUNG_03146", "MHUNG_03150", "MHUNG_03183",
                  "MHUNG_00119", "MHUNG_00543", "MHUNG_01072", "MHUNG_00118", "MHUNG_00967", "MHUNG_01033",
"MHUNG_01034", "MHUNG_01071", "MHUNG_00121",
                  "MHUNG_01248", "MHUNG_02870", "MHUNG_02867", "MHUNG_00120", "MHUNG_00134", "MHUNG_01042",
"MHUNG_01076", "MHUNG_00117", "MHUNG_00136",
                  "MHUNG_00341", "MHUNG_01309", "MHUNG_01562", "MHUNG_01844", "MHUNG_02109", "MHUNG_02819",
"MHUNG_02869", "MHUNG_02876", "MHUNG_03079",
                  "MHUNG_03243", "MHUNG_03249", "MHUNG_03250", "MHUNG_00006", "MHUNG_00545", "MHUNG_00978",
"MHUNG_01075", "MHUNG_01077", "MHUNG_01546",
                  "MHUNG_01778", "MHUNG_02067", "MHUNG_02565", "MHUNG_02706", "MHUNG_02707", "MHUNG_02724",

                  "MHUNG_01927", "MHUNG_01928", "MHUNG_02620", "MHUNG_03373", "MHUNG_03416", "MHUNG_00207",
"MHUNG_00210", "MHUNG_00211", "MHUNG_00212",
                  "MHUNG_00217", "MHUNG_00218", "MHUNG_00222", "MHUNG_00227", "MHUNG_00228", "MHUNG_00229",
"MHUNG_00231", "MHUNG_00232", "MHUNG_00233",
                  "MHUNG_00235", "MHUNG_00254", "MHUNG_00255", "MHUNG_00256", "MHUNG_00257", "MHUNG_03344",
"MHUNG_03345", "MHUNG_02388", "MHUNG_03230",
                  "MHUNG_03231")

#match the list of interesting genes in the order given in vector to select these genes from the vsd of the
DEseq
mhung.second1.mmh.ordered <- mhung_mmh_heatmap_data[mhung.second1,]

# for selecting rownames of the future heatmap to have other values than only your locus_tags (current
rowname)mhung.hyd.mmh.ordered$gp_name[row.names(mhung.hyd.mmh.ordered)=="MHUNG_02758"] <- " Coenzyme F420
hydrogenase subunit alpha"
mhung.second1.mmh.rownames <- mhung.second1.mmh.ordered %>%
  rownames_to_column(., var = "locus_tag") %>%
  select(c("locus_tag")) %>%
  unite(., col='rownames', c("locus_tag"))

#print heatmap in screen
heat_second1_mhung_mmh <- pheatmap(mhung.second1.mmh.ordered[,1:6], cluster_rows=FALSE, show_rownames=TRUE,
         cluster_cols=FALSE,
         main = "Expression of genes from secondary metabolism of M. hungatei in Sf-Mh (EPS, flagella, pili)",
         labels_row = mhung.second1.mmh.rownames[,1])#,
         #annotation_row adds another column with e.g. calculated LFC or p-values
         #annotation_row = mpob.prop.ox.mmh[,c("log2FoldChange", "padj")])

#save heatmap as png
png("20230530_Secondary1_Mhung_inMMH.png", width = 3000, height = 5200, res = 250)
pheatmap(mhung.second1.mmh.ordered[,1:6], cluster_rows=FALSE, show_rownames=TRUE,
         #cellheight = 10,
         #cellwidth = 35,
         cluster_cols=FALSE,
         main = "Expression of genes from secondary metabolism of M. hungatei in Sf-Mh (EPS, flagella, pili)",
         labels_row = mhung.second1.mmh.rownames[,1],
         gaps_row = c(13, 64),
         #annotation_row adds another column with e.g. calculated LFC or p-values)
         annotation_row = mhung.second1.mmh.ordered[,c("logFC", "p_adj")],
         annotation_colors = ann_colors,
```

```
         border_color = NA
         )
dev.off()


###or save the heatmap as svg
imageHeatmap.Mhung.MMH.second1 = pheatmap(mhung.second1.mmh.ordered[,1:6], cluster_rows=FALSE,
show_rownames=TRUE,
         cluster_cols=FALSE,
         main = "Expression of genes from secondary metabolism of M. hungatei in Sf-Mh (EPS, flagella, pili)",
         labels_row = mhung.second1.mmh.rownames[,1],
         gaps_row = c(13, 64),
         #annotation_row adds another column with e.g. calculated LFC or p-values)
         annotation_row = mhung.second1.mmh.ordered[,c("logFC", "p_adj")],
         annotation_colors = ann_colors,
         border_color = NA)
ggsave("Secondary1.Mhung.MMH.svg", plot = imageHeatmap.Mhung.MMH.second1, width=15, height=15)

####secondary 2
#match the list of interesting genes in the order given in vector to select these genes from the vsd of the
DEseq
mhung.second2.mmh.ordered <- mhung_mmh_heatmap_data[mhung.second2,]

# for selecting rownames of the future heatmap to have other values than only your locus_tags (current
rowname)mhung.hyd.mmh.ordered$gp_name[row.names(mhung.hyd.mmh.ordered)=="MHUNG_02758"] <- " Coenzyme F420
hydrogenase subunit alpha"
mhung.second2.mmh.rownames <- mhung.second2.mmh.ordered %>%
  rownames_to_column(., var = "locus_tag") %>%
  select(c("locus_tag")) %>%
  unite(., col='rownames', c("locus_tag"))

#print heatmap in screen
heat_second2_mhung_mmh <- pheatmap(mhung.second2.mmh.ordered[,1:6], cluster_rows=FALSE, show_rownames=TRUE,
         cluster_cols=FALSE,
         main = "Expression of genes from secondary metabolism of M. hungatei in Sf-Mh (Chemotaxis,
signalling)",
         labels_row = mhung.second2.mmh.rownames[,1])#,
         #annotation_row adds another column with e.g. calculated LFC or p-values
         #annotation_row = mpob.prop.ox.mmh[,c("log2FoldChange", "padj")])

#save heatmap as png
png("20230526_Secondary2_Mhung_inMMH.png", width = 3000, height = 5200, res = 250)
pheatmap(mhung.second2.mmh.ordered[,1:6], cluster_rows=FALSE, show_rownames=TRUE,
         #cellheight = 10,
         #cellwidth = 35,
         cluster_cols=FALSE,
         main = "Expression of genes from secondary metabolism of M. hungatei in Sf-Mh (Chemotaxis,
signalling)",
         labels_row = mhung.second2.mmh.rownames[,1],
         gaps_row = c(69),
         #annotation_row adds another column with e.g. calculated LFC or p-values)
         annotation_row = mhung.second2.mmh.ordered[,c("logFC", "p_adj")],
         annotation_colors = ann_colors,
         border_color = NA
         )
dev.off()


###or save the heatmap as svg
imageHeatmap.Mhung.MMH.second2 = pheatmap(mhung.second2.mmh.ordered[,1:6], cluster_rows=FALSE,
show_rownames=TRUE,
         cluster_cols=FALSE,
         main = "Expression of genes from secondary metabolism of M. hungatei in Sf-Mh (Chemotaxis,
signalling)",
         labels_row = mhung.second2.mmh.rownames[,1],
          gaps_row = c(69),
         #annotation_row adds another column with e.g. calculated LFC or p-values)
         annotation_row = mhung.second2.mmh.ordered[,c("logFC", "p_adj")],
         annotation_colors = ann_colors,
         border_color = NA)
ggsave("Secondary2.Mhung.MMH.svg", plot = imageHeatmap.Mhung.MMH.second2, width=15, height=15)
```

Study 2 Differential Expression analysis: cocultures of Syntrophobacter fumaroxidans MPOB and Methanobacterium formicicum

```{r DEseq Sfum-MPOB for Sf-Mf_cycle1 and Sf-Mf_cycle20 data}
#Look at DSEQ guide for how to use this commands http://bioconductor.org/packages/release/bioc/vignettes
/DESeq2/inst/doc/DESeq2.html
```

````
#global condition
condition_mpob_mmk<-as.factor(c("null","MMK1","MMK1","MMK1","MMK20","MMK20","MMK20"))
file_mpob_mmk<- colnames(df_counts_mpob)


rep_condt_mpob_mmk<-data_frame(file_mpob_mmk[c(14:19)],condition_mpob_mmk[c(-1)])
colnames(rep_condt_mpob_mmk)<-c("replicates","conditions")


df.counts.mpob.2<-df_counts_mpob %>% remove_rownames %>%
column_to_rownames(var="locus_tag")

df.counts.mpob.2[is.na(df.counts.mpob.2)] <- 0


##DESeq is coming now
dds_mpob_mmk <- DESeqDataSetFromMatrix(countData = df.counts.mpob.2[,c(13:18)] ,
                              colData = rep_condt_mpob_mmk,
                              design= ~ conditions)
dds_mpob_mmk$conditions <- relevel(dds_mpob_mmk$conditions, ref = "MMK1")

dds_mpob_mmk <- DESeq(dds_mpob_mmk)
resultsNames(dds_mpob_mmk)

#Plot PCA
 vsd_mpob_mmk <- vst(dds_mpob_mmk, blind=FALSE)
tiff("PCA_MMK1vsMMK20_mpob.may1.tiff", units="in", width=10, height=2, res=300)
 plotPCA(vsd_mpob_mmk, intgroup=c("conditions"))
dev.off()


norm.counts<- counts(dds_mpob_mmk,normalized=TRUE)
df.norm.counts.mpob.mmk<-data.frame(norm.counts)


write.csv(df.norm.counts.mpob.mmk, file ="DEseq_normCounts_mpob_inMMK_flocs.may1.csv", row.names = TRUE)

```


```{r Sfumaroxidans MPOB in Sf-Mf_cycle1 VS MPOB in Sf-Mf_cycle20}
# TPMs are used here now, calculated earlier

# this a function to obtain the results as a dataframe.
results_MMK1vsMMK20_mpob <- results(dds_mpob_mmk, contrast=c("conditions","MMK1","MMK20"))

#renaming the rownames for the properly lookin glocus tag
row.names(results_MMK1vsMMK20_mpob) <- gsub("EPMPDPEG", "MPOB", row.names(results_MMK1vsMMK20_mpob))


#create a volcano plot of the results
#tiff("DEseq_MMK1vsMMK20_mpob.tiff", units="in", width=15, height=15, res=300)
image3 = EnhancedVolcano(results_MMK1vsMMK20_mpob,
    lab = rownames(results_MMK1vsMMK20_mpob),
    x = 'log2FoldChange',
    y = 'padj',
    FCcutoff = 1,
    legendPosition = "right")
ggsave("DEseq_MMK1vsMMK20_mpob.svg", plot = image3, width=15, height=15)
#dev.off()

#renaming the rownames back to match the tpm analysis files
row.names(results_MMK1vsMMK20_mpob) <- gsub("MPOB", "EPMPDPEG", row.names(results_MMK1vsMMK20_mpob))


#convert the locus_tags from the rows into a column of there own.
df.res.MMK1vsMMK20.mpob<- data.frame(results_MMK1vsMMK20_mpob) %>%
  rownames_to_column(var="locus_tag")



df.res.MMK1vsMMK20.mpob.exp.all<-df.res.MMK1vsMMK20.mpob%>%
  #left_join(df.norm.counts.ace.1,by="locus_tag") %>%
  left_join(df.tpm.analysis.mpob.mmk,by="locus_tag") %>%
  distinct(locus_tag,.keep_all=TRUE) #%>%
   #select(-8,-10,-12:-14,-18,-20,-24:-29)

#store the rds for later use.
write_rds(df.res.MMK1vsMMK20.mpob.exp.all,file.path(data.tables.dir,"Differential_analysis.mpob.mmk.rds"))

write.csv(df.res.MMK1vsMMK20.mpob.exp.all, file ="DEseq_all_mpob_inMMK_flocs.csv", row.names = FALSE)
````

````
```


``` {r Heatmaps for Sfumaroxidans MPOB in Sf-Mf_cycle1 and Sf-Mf_cycle20 data}

##A heatmap of the interesting DEseq genes per set of functions
#Create a heatmap usable data from normalized vsd_* expression from DEseq
#Make the locus_tags the rownames
##the command left_join below used "all.x=TRUE" before 2023 R version. Now it is deprecated, and you do not
need to pass that argument any more.
mpob_mmk_heatmap_data <- data.frame(assay(vsd_mpob_mmk)) %>%
  rownames_to_column(var="locus_tag")  %>%
  left_join(df.res.MMK1vsMMK20.mpob.exp.all[,c("locus_tag", "baseMean", "log2FoldChange", "padj", "gp_name")],
by="locus_tag") %>%
  #Make the locus_tags the rownames
 'rownames<-'(.$locus_tag) %>%
  select(-"locus_tag") %>%
  mutate_at(c(1:9), as.numeric) %>%
  #Make categories for log2foldchange and adjusted p-values to include in heatmaps
  mutate(logFC = case_when(log2FoldChange < 0.585 & log2FoldChange >= -0.585 ~ '<1.5',
                           log2FoldChange < 1.0  & log2FoldChange >= 0.585 ~ '1.5-2.0',
                           log2FoldChange > -1.0  & log2FoldChange <= -0.585 ~ '1.5-2.0',
                           log2FoldChange >= 1.0 ~ '>2',
                           log2FoldChange <= -1.0 ~ '>2')) %>%
  mutate(p_adj = case_when(padj >= 0.05  ~ '>0.05',
                            padj < 0.05 & padj >= 0.01 ~ '0.01-0.05',
                            padj < 0.01 ~ '<0.01'))

row.names(mpob_mmk_heatmap_data) <- gsub("EPMPDPEG", "MPOB", row.names(mpob_mmk_heatmap_data))

#making a colour palette for the LogFC and p_adj to be used in th heatmap
ann_colors <- list(logFC=c('<1.5'="#fff0f0", '1.5-2.0'="#ffa5c6",'>2'="#ef447b"),
                   p_adj=c('>0.05'="#c5e8c6", '0.01-0.05'="#60851f", '<0.01'="#015a51"))


###Central metabolism of Sfumaroxidans MPOB (oxidation of propionate, hydrogenases, formate dehydrogenases)
mpob.central <- c("MPOB_04019", "MPOB_04020", "MPOB_04021", "MPOB_04022", "MPOB_04023", "MPOB_04024",
"MPOB_04025", "MPOB_04026",
                  "MPOB_00467", "MPOB_00468", "MPOB_00469", "MPOB_00470", "MPOB_01750", "MPOB_01751",
"MPOB_02057", "MPOB_02058",
                  "MPOB_02059", "MPOB_04186", "MPOB_04187", "MPOB_04188", "MPOB_04189", "MPOB_00186",
"MPOB_00187", "MPOB_02164",
                  "MPOB_02165", "MPOB_02162", "MPOB_02163", "MPOB_02395", "MPOB_00473", "MPOB_00474",
"MPOB_01267", "MPOB_02868",
                  "MPOB_02869", "MPOB_02870", "MPOB_02871", "MPOB_00399", "MPOB_00400", "MPOB_00775",
"MPOB_01322", "MPOB_03151",
                  "MPOB_00843", "MPOB_00844", "MPOB_00845", "MPOB_01176", "MPOB_01177", "MPOB_01178",

                  "MPOB_00878", "MPOB_00879", "MPOB_00880", "MPOB_01840", "MPOB_01841", "MPOB_01843",
"MPOB_01844", "MPOB_02285",
                  "MPOB_02286", "MPOB_02287", "MPOB_02288", "MPOB_02788", "MPOB_02789", "MPOB_02790",
"MPOB_02791", "MPOB_02792",
                  "MPOB_03618", "MPOB_03619", "MPOB_03620", "MPOB_04046", "MPOB_04047", "MPOB_04049",
"MPOB_04048", "MPOB_00881",
                  "MPOB_00882", "MPOB_03032", "MPOB_03033",

                  "MPOB_01845", "MPOB_01846", "MPOB_01847", "MPOB_01851", "MPOB_01852", "MPOB_01854",
"MPOB_01855", "MPOB_01856",
                  "MPOB_01857", "MPOB_01858", "MPOB_02779", "MPOB_02780", "MPOB_02781", "MPOB_02782",
"MPOB_00033", "MPOB_00034",
                  "MPOB_00039", "MPOB_00040", "MPOB_00041", "MPOB_01318", "MPOB_01319", "MPOB_01320",
"MPOB_03593", "MPOB_03594",
                  "MPOB_02783")

#match the list of interesting genes in the order given in vector to select these genes from the vsd of the
DEseq
mpob.central.mmk.ordered <- mpob_mmk_heatmap_data[mpob.central,]

# for selecting rownames of the future heatmap to have other values than only your locus_tags (current
rowname)
mpob.central.mmk.rownames <- mpob.central.mmk.ordered %>%
  rownames_to_column(., var = "locus_tag") %>%
  select(c("locus_tag")) %>%
  unite(., col='rownames', c("locus_tag"))

#print heatmap in screen
heat_central_mpob_mmk <- pheatmap(mpob.central.mmk.ordered[,1:6], cluster_rows=FALSE, show_rownames=TRUE,
         cluster_cols=FALSE,
         main = "Expression of genes from central metabolism of S. fumaroxidans in Sf-Mf",
         labels_row = mpob.central.mmk.rownames[,1])#,
````

```
         #annotation_row adds another column with e.g. calculated LFC or p-values
         #annotation_row = mpob.prop.ox.mmh[,c("log2FoldChange", "padj")])

#save heatmap as png
png("20230526_central_MPOB_inMMK.png", width = 3000, height = 5200, res = 250)
pheatmap(mpob.central.mmk.ordered[,1:6], cluster_rows=FALSE, show_rownames=TRUE,
         cluster_cols=FALSE,
         main = "Expression of genes from central metabolism of S. fumaroxidans in Sf-Mf",
         labels_row = mpob.central.mmk.rownames[,1],
         gaps_row = c(46, 73),
         #annotation_row adds another column with e.g. calculated LFC or p-values)
         annotation_row = mpob.central.mmk.ordered[,c("logFC", "p_adj")],
         annotation_colors = ann_colors,
         border_color = NA
         )
dev.off()

###or save the heatmap as svg
imageHeatmap.MPOB.MMK.central = pheatmap(mpob.central.mmk.ordered[,1:6], cluster_rows=FALSE,
show_rownames=TRUE,
         cluster_cols=FALSE,
         main = "Expression of genes from central metabolism of S. fumaroxidans in Sf-Mf",
         labels_row = mpob.central.mmk.rownames[,1],
         gaps_row = c(46, 73),
         #annotation_row adds another column with e.g. calculated LFC or p-values)
         annotation_row = mpob.central.mmk.ordered[,c("logFC", "p_adj")],
         annotation_colors = ann_colors,
         border_color = NA
         )
ggsave("Heatmap_MPOB_MMK_central.svg", plot = imageHeatmap.MPOB.MMK.central, width=15, height=15)


##Secondary metabolism in Sfumaroxidans MPOB (EPS, metal transporters, signal transduction, pili, flagella,
chemotaxis)
mpob.second <- c("MPOB_03180", "MPOB_02373", "MPOB_02343", "MPOB_02204", "MPOB_00892", "MPOB_00871",
"MPOB_00324", "MPOB_03438", "MPOB_03439",
                 "MPOB_00203", "MPOB_00202", "MPOB_02131", "MPOB_00682", "MPOB_00684", "MPOB_02566",
"MPOB_03841", "MPOB_03842",
                 "MPOB_02243", "MPOB_02244", "MPOB_02245", "MPOB_02246", "MPOB_02247", "MPOB_02248",
"MPOB_02249", "MPOB_02250", "MPOB_02251", "MPOB_02252", "MPOB_00999", "MPOB_03415", "MPOB_00130",
"MPOB_01009",
                 "MPOB_01010", "MPOB_01011", "MPOB_01012", "MPOB_01013", "MPOB_01014", "MPOB_01015",
"MPOB_01016", "MPOB_00806",
                 "MPOB_03402", "MPOB_03403", "MPOB_03404", "MPOB_03405", "MPOB_03406", "MPOB_03407",
"MPOB_03408", "MPOB_03409", "MPOB_03410",
                 "MPOB_02703", "MPOB_02704", "MPOB_00495", "MPOB_00613", "MPOB_01373", "MPOB_03455",

                 "MPOB_00030", "MPOB_00031", "MPOB_00032", "MPOB_00043", "MPOB_03786", "MPOB_03787",
"MPOB_02014", "MPOB_02976",
                 "MPOB_02977", "MPOB_02978", "MPOB_02559", "MPOB_02560", "MPOB_02561", "MPOB_02826",
"MPOB_01141", "MPOB_01798",
                 "MPOB_00512", "MPOB_00028", "MPOB_02810", "MPOB_02809", "MPOB_03310",

                 "MPOB_01256", "MPOB_02599", "MPOB_02130", "MPOB_01429", "MPOB_04042", "MPOB_01428",
"MPOB_03379", "MPOB_02631",
                 "MPOB_02632", "MPOB_02633", "MPOB_02634", "MPOB_01742", "MPOB_01743", "MPOB_00331",
"MPOB_01704", "MPOB_00553",
                 "MPOB_00554", "MPOB_00131", "MPOB_02153", "MPOB_00138", "MPOB_00555", "MPOB_00129",
"MPOB_00347", "MPOB_00443",
                 "MPOB_00552", "MPOB_00957", "MPOB_00985", "MPOB_01533", "MPOB_02403", "MPOB_03468",
"MPOB_03750", "MPOB_03975",
                 "MPOB_03976",

                 "MPOB_01696", "MPOB_01697", "MPOB_01698", "MPOB_01699", "MPOB_01700", "MPOB_01701",
"MPOB_01702","MPOB_03377",

                 "MPOB_03835","MPOB_02531", "MPOB_03663", "MPOB_04123", "MPOB_03330", "MPOB_01563",
"MPOB_00432", "MPOB_02017",
                 "MPOB_03626", "MPOB_02720", "MPOB_02271", "MPOB_00245", "MPOB_02733", "MPOB_00984",
"MPOB_00977", "MPOB_03783",
                 "MPOB_03051", "MPOB_00557", "MPOB_00120",  "MPOB_02419", "MPOB_02422", "MPOB_02425",
"MPOB_02358", "MPOB_02219",
                 "MPOB_01147", "MPOB_01148", "MPOB_01149", "MPOB_00341", "MPOB_02677", "MPOB_00836",
"MPOB_01457", "MPOB_00747",
                 "MPOB_01566", "MPOB_01973", "MPOB_03338", "MPOB_01058")



#match the list of interesting genes in the order given in vector to select these genes from the vsd of the
DEseq
mpob.second.mmk.ordered <- mpob_mmk_heatmap_data[mpob.second,]
```

````
# for selecting rownames of the future heatmap to have other values than only your locus_tags (current
rowname)
mpob.second.mmk.rownames <- mpob.second.mmk.ordered %>%
  rownames_to_column(., var = "locus_tag") %>%
  select(c("locus_tag")) %>%
  unite(., col='rownames', c("locus_tag"))

#print heatmap in screen
heat_second_mpob_mmk <- pheatmap(mpob.second.mmk.ordered[,1:6], cluster_rows=FALSE, show_rownames=TRUE,
         cluster_cols=FALSE,
         main = "Expression of genes from secondary metabolism of S. fumaroxidans in Sf-Mf",
         labels_row = mpob.second.mmk.rownames[,1])#,
         #annotation_row adds another column with e.g. calculated LFC or p-values
         #annotation_row = mpob.prop.ox.mmh[,c("log2FoldChange", "padj")])

#save heatmap as png
png("20230530_secondary_MPOB_inMMK.png", width = 3000, height = 5200, res = 250)
pheatmap(mpob.second.mmk.ordered[,1:6], cluster_rows=FALSE, show_rownames=TRUE,
         cluster_cols=FALSE,
         main = "Expression of genes from secondary metabolism of S. fumaroxidans in Sf-Mf",
         labels_row = mpob.second.mmk.rownames[,1],
         gaps_row = c(54, 75, 108, 116),
         #annotation_row adds another column with e.g. calculated LFC or p-values)
         annotation_row = mpob.second.mmk.ordered[,c("logFC", "p_adj")],
         annotation_colors = ann_colors,
         border_color = NA
         )
dev.off()

###or save the heatmap as svg
imageHeatmap.MPOB.MMK.second = pheatmap(mpob.second.mmk.ordered[,1:6], cluster_rows=FALSE, show_rownames=TRUE,
         cluster_cols=FALSE,
         main = "Expression of genes from secondary metabolism of S. fumaroxidans in Sf-Mf",
         labels_row = mpob.second.mmk.rownames[,1],
         gaps_row = c(54, 75, 108, 116),
         #annotation_row adds another column with e.g. calculated LFC or p-values)
         annotation_row = mpob.second.mmk.ordered[,c("logFC", "p_adj")],
         annotation_colors = ann_colors,
         border_color = NA
         )
ggsave("Heatmap_MPOB_MMK_secondary.svg", plot = imageHeatmap.MPOB.MMK.second, width=15, height=15)
```

```{r DEseq Mformicicum for Sf-Mf_cycle1 (MMK1) and Sf-Mf_cycle20 (MMK20) data}
##
#global condition
condition_form_mmk<-as.factor(c("null","MMK1","MMK1","MMK1","MMK20","MMK20","MMK20"))
file_form_mmk<- colnames(df_counts_form)


rep_condt_form_mmk<-data_frame(file_form_mmk[c(14:19)],condition_form_mmk[c(-1)])
colnames(rep_condt_form_mmk)<-c("replicates","conditions")


df.counts.form.4<-df_counts_form %>% remove_rownames %>%
column_to_rownames(var="locus_tag")

df.counts.form.4[is.na(df.counts.form.4)] <- 0


##DESeq is coming now
dds_form_mmk <- DESeqDataSetFromMatrix(countData = df.counts.form.4[,c(13:18)] ,
                              colData = rep_condt_form_mmk,
                              design= ~ conditions)
dds_form_mmk$conditions <- relevel(dds_form_mmk$conditions, ref = "MMK1")

dds_form_mmk <- DESeq(dds_form_mmk)
resultsNames(dds_form_mmk)

#Plot PCA
vsd_form_mmk <- vst(dds_form_mmk, blind=FALSE)
tiff("PCA_MMK1vsMMK20_form.may1.tiff", units="in", width=10, height=2, res=300)
 plotPCA(vsd_form_mmk, intgroup=c("conditions"))
dev.off()


#Extracting normalized counts of the DESeq
norm.counts<- counts(dds_form_mmk,normalized=TRUE)
df.norm.counts.form.mmk<-data.frame(norm.counts)
````

````
write.csv(df.norm.counts.form.mmk, file ="DEseq_normCounts_form_inMMK_flocs.may1.csv", row.names = TRUE)

```

```{r Mformicicum in Sf-Mf_cycle1 (MMK1) VS Mformicicum in Sf-Mf_cycle20 (MMK20)}
# TPMs are used here now, calculated earlier


# this a function to obtain the results as a dataframe.
results_MMK1vsMMK20_form <- results(dds_form_mmk, contrast=c("conditions", "MMK1", "MMK20" ))

#renaming the rownames for the properly looking locus tag
row.names(results_MMK1vsMMK20_form) <- gsub("PHHPAKOO","MFORM", row.names(results_MMK1vsMMK20_form))


#create a volcano plot of the results
#tiff("DEseq_MMK1vsMMK20_form.tiff", units="in", width=15, height=15, res=300)
image4 = EnhancedVolcano(results_MMK1vsMMK20_form,
    lab = rownames(results_MMK1vsMMK20_form),
    x = 'log2FoldChange',
    y = 'padj',
    FCcutoff = 1,
    legendPosition = "right" )
ggsave("DEseq_MMK1vsMMK20_form.may1.svg", plot = image4, width=15, height=15)
#dev.off()

#renaming back the rownames to match TPM data
row.names(results_MMK1vsMMK20_form) <- gsub("MFORM", "PHHPAKOO", row.names(results_MMK1vsMMK20_form))


#convert the locus_tags from the rows into a column of there own.
df.res.MMK1vsMMK20.form<- data.frame(results_MMK1vsMMK20_form) %>%
  rownames_to_column(var="locus_tag")



df.res.MMK1vsMMK20.form.exp.all<-df.res.MMK1vsMMK20.form%>%
  #left_join(df.norm.counts.ace.1,by="locus_tag") %>%
  left_join(df.tpm.analysis.form.mmk,by="locus_tag") %>%
  distinct(locus_tag,.keep_all=TRUE) #%>%
   #select(-8,-10,-12:-14,-18,-20,-24:-29)

#store the rds for later use.
write_rds(df.res.MMK1vsMMK20.form.exp.all,file.path(data.tables.dir,"Differential_analysis.form.mmk.may1.rds"))

write.csv(df.res.MMK1vsMMK20.form.exp.all, file ="DEseq_all_form_inMMK_flocs.may1.csv", row.names = FALSE)
```


```{r Heatmap Mform Sf-Mf_cycle1 (MMK1) VS Mformicicum in Sf-Mf_cycle20 (MMK20)}
#Create a heatmap usable data from normalized vsd_* expression from DEseq
#Make the locus_tags the rownames
##the command left_join below used "all.x=TRUE" before 2023 R version. Now it is deprecated, and you do not
need to pass that argument any more.
mform_mmk_heatmap_data <- data.frame(assay(vsd_form_mmk)) %>%
  rownames_to_column(var="locus_tag")  %>%
  left_join(df.res.MMK1vsMMK20.form.exp.all[,c("locus_tag", "baseMean", "log2FoldChange", "padj", "gp_name")],
by="locus_tag") %>%
  #Make the locus_tags the rownames
  'rownames<-'(.$locus_tag) %>%
  select(-"locus_tag") %>%
  mutate_at(c(1:9), as.numeric) %>%
  #Make categories for log2foldchange and adjusted p-values to include in heatmaps
  mutate(logFC = case_when(log2FoldChange < 0.585 & log2FoldChange >= -0.585 ~ '<1.5',
                           log2FoldChange < 1.0  & log2FoldChange >= 0.585 ~ '1.5-2.0',
                           log2FoldChange > -1.0  & log2FoldChange <= -0.585 ~ '1.5-2.0',
                           log2FoldChange >= 1.0 ~ '>2',
                           log2FoldChange <= -1.0 ~ '>2')) %>%
  mutate(p_adj = case_when(padj >= 0.05  ~ '>0.05',
                            padj < 0.05 & padj >= 0.01 ~ '0.01-0.05',
                            padj < 0.01 ~ '<0.01'))

row.names(mform_mmk_heatmap_data) <- gsub("PHHPAKOO", "Mform", row.names(mform_mmk_heatmap_data))

#making a colour palette for the LogFC and p_adj to be used in th heatmap
ann_colors <- list(logFC=c('<1.5'="#fff0f0", '1.5-2.0'="#ffa5c6",'>2'="#ef447b"),
                   p_adj=c('>0.05'="#c5e8c6", '0.01-0.05'="#60851f", '<0.01'="#015a51"))


###Central metabolism M. formicicum (hydrogenotrophic methanogenesis, hydrogenases, formate dehydrogenases,
````

```
assimilation of CO2/acetate into biomass)

mform.central <- c("Mform_01910", "Mform_01911", "Mform_01517", "Mform_01518", "Mform_00112", "Mform_01164",
               "Mform_01163", "Mform_01162", "Mform_01181", "Mform_01182", "Mform_01183", "Mform_01184",
               "Mform_01185", "Mform_01186", "Mform_01187", "Mform_01188", "Mform_01189", "Mform_01190",
               "Mform_01191", "Mform_01192", "Mform_01193", "Mform_01194", "Mform_00614", "Mform_00612",
               "Mform_00611", "Mform_00605", "Mform_00473", "Mform_01048", "Mform_01044", "Mform_01752",
               "Mform_01711", "Mform_01710", "Mform_01709", "Mform_01708", "Mform_01707", "Mform_01706",
               "Mform_01684", "Mform_01675", "Mform_00845", "Mform_00256", "Mform_00281", "Mform_00282",
               "Mform_00289", "Mform_00732",

               "Mform_00238", "Mform_00239", "Mform_00240", "Mform_00241", "Mform_00242", "Mform_00243",
               "Mform_00244", "Mform_00245", "Mform_00246", "Mform_00247", "Mform_00248", "Mform_00249",
               "Mform_00250", "Mform_00251", "Mform_00252", "Mform_00253", "Mform_00254", "Mform_00255",
               "Mform_00638", "Mform_00637", "Mform_00636", "Mform_00635", "Mform_00634", "Mform_00633",
               "Mform_00632", "Mform_00631", "Mform_00630", "Mform_00629", "Mform_00628", "Mform_00627",
               "Mform_00626", "Mform_00625", "Mform_00624", "Mform_00623", "Mform_01075",

               "Mform_01717", "Mform_01718", "Mform_01720", "Mform_01697",

               "Mform_02036", "Mform_00872", "Mform_02234", "Mform_01374", "Mform_01648", "Mform_00653",
               "Mform_00654", "Mform_00655", "Mform_00656", "Mform_00681", "Mform_00682", "Mform_00684",
               "Mform_00685", "Mform_00686", "Mform_00657", "Mform_00658",

               "Mform_02204", "Mform_01534")


mform.central.mmk.ordered <- mform_mmk_heatmap_data[mform.central,]
mform.central.mmk.rownames <- mform.central.mmk.ordered %>%
  rownames_to_column(., var = "locus_tag") %>%
  select(c("locus_tag")) %>%
  unite(., col='rownames', c("locus_tag"))

png("20230526_central_Mform_inMMK.png", width = 3000, height = 4200, res = 250)
pheatmap(mform.central.mmk.ordered[,1:6], cluster_rows=FALSE, show_rownames=TRUE,
         #cellheight = 10,
         #cellwidth = 35,
         cluster_cols=FALSE,
         main = "Transcription of genes from central metabolism in M. formicicum",
         labels_row = mform.central.mmk.rownames[,1],
         gaps_row = c(44, 79, 83, 99),
         #annotation_row adds another column with e.g. calculated LFC or p-values)
         annotation_row = mform.central.mmk.ordered[,c("logFC", "p_adj")],
         annotation_colors = ann_colors,
         border_color = NA
         )
dev.off()

###or save the heatmap as svg
imageHeatmap.Mform.MMK.central = pheatmap(mform.central.mmk.ordered[,1:6], cluster_rows=FALSE,
show_rownames=TRUE,
         cluster_cols=FALSE,
         main = "Transcription of genes from central metabolism in M. formicicum",
         labels_row = mform.central.mmk.rownames[,1],
         gaps_row = c(44, 79, 83, 99),
         #annotation_row adds another column with e.g. calculated LFC or p-values)
         annotation_row = mform.central.mmk.ordered[,c("logFC", "p_adj")],
         annotation_colors = ann_colors,
         border_color = NA)
ggsave("Central.SystemsGenes.Mform.MMK.svg", plot = imageHeatmap.Mform.MMK.central, width=15, height=15)

###Secondary metabolism of M. formicicum (EPS, flagella, metal transporters, signal transduction)
##gaps after 13, 39, 44, 52,
mform.second <- c("Mform_00409", "Mform_00842", "Mform_00836", "Mform_00331", "Mform_01656", "Mform_00604",
               "Mform_01653", "Mform_01154", "Mform_01462", "Mform_01461", "Mform_01018", "Mform_00013",
               "Mform_00235",

               "Mform_01376", "Mform_00771", "Mform_00770", "Mform_00769", "Mform_01698", "Mform_01699",
               "Mform_01049", "Mform_01680", "Mform_01679", "Mform_01678", "Mform_01052", "Mform_01051",
               "Mform_00962", "Mform_00963", "Mform_00964", "Mform_01480", "Mform_01479", "Mform_01478",
               "Mform_01477", "Mform_01476", "Mform_01475", "Mform_00765", "Mform_00762", "Mform_00761",
               "Mform_00760", "Mform_00759",

               "Mform_00688", "Mform_00687", "Mform_02311", "Mform_01543", "Mform_01544",

               "Mform_01307", "Mform_02114", "Mform_00118", "Mform_01967", "Mform_01966", "Mform_02148",
               "Mform_00499", "Mform_00875",

               "Mform_00667", "Mform_00668", "Mform_02294", "Mform_01579", "Mform_02197", "Mform_01955",
               "Mform_00398", "Mform_00396", "Mform_01585", "Mform_00780", "Mform_01670", "Mform_02036",
               "Mform_01953", "Mform_01979", "Mform_01978", "Mform_01954", "Mform_01352", "Mform_00360",
```

```
                "Mform_02013", "Mform_01229", "Mform_01071", "Mform_00030", "Mform_00031", "Mform_01660",
                "Mform_01561", "Mform_01568", "Mform_01569", "Mform_01421", "Mform_01420", "Mform_00161",
                "Mform_01488", "Mform_01789", "Mform_01062", "Mform_02148", "Mform_00875", "Mform_00499")


mform.second.mmk.ordered <- mform_mmk_heatmap_data[mform.second,]
mform.second.mmk.rownames <- mform.second.mmk.ordered %>%
  rownames_to_column(., var = "locus_tag") %>%
  select(c("locus_tag")) %>%
  unite(., col='rownames', c("locus_tag"))

png("20230526_secondary_Mform_inMMK.png", width = 3000, height = 4200, res = 250)
pheatmap(mform.second.mmk.ordered[,1:6], cluster_rows=FALSE, show_rownames=TRUE,
         #cellheight = 10,
         #cellwidth = 35,
         cluster_cols=FALSE,
         main = "Transcription of genes from secondary metabolism in M. formicicum",
         labels_row = mform.second.mmk.rownames[,1],
         gaps_row = c(13, 39, 44, 52),
         #annotation_row adds another column with e.g. calculated LFC or p-values)
         annotation_row = mform.second.mmk.ordered[,c("logFC", "p_adj")],
         annotation_colors = ann_colors,
         border_color = NA
         )
dev.off()

###or save the heatmap as svg
imageHeatmap.Mform.MMK.second = pheatmap(mform.second.mmk.ordered[,1:6], cluster_rows=FALSE,
show_rownames=TRUE,
         cluster_cols=FALSE,
         main = "Transcription of genes from secondary metabolism in M. formicicum",
         labels_row = mform.second.mmk.rownames[,1],
         gaps_row = c(13, 39, 44, 52),
         #annotation_row adds another column with e.g. calculated LFC or p-values)
         annotation_row = mform.second.mmk.ordered[,c("logFC", "p_adj")],
         annotation_colors = ann_colors,
         border_color = NA)
ggsave("Secondary.SystemsGenes.Mform.MMK.svg", plot = imageHeatmap.Mform.MMK.second, width=15, height=15)
```

```r
# General settings

## General directories
root.dir <- here::here()
data.dir <- file.path(root.dir, "data")
data.raw.dir  <- file.path(data.dir, "raw")
data.processed.dir  <- file.path(data.dir, "processed")
data.script<- file.path(root.dir,"scripts")
functions.dir <- file.path(root.dir, "functions")

#Raw directories
data.interpro.dir<-file.path(data.raw.dir,"interproscan_domains")
data.kegg.dir<-file.path(data.raw.dir,"KEGG_KO")
data.raw.counts.dir<-file.path(data.raw.dir,"prokka_counts")
data.ghost.dir<-file.path(data.raw.dir,"Prokka_ghost")
data.hmm.dir<-file.path(data.raw.dir,"Prokka_HMM")
data.protein.dir<-file.path(data.raw.dir,"prokka_protein_files")
data.gene.dir<-file.path(data.raw.dir,"prokka_gene_files")
data.raw.gff.dir<-file.path(data.raw.dir,"prokka_gff")

#processed directories
data.p.interpro.dir<-file.path(data.processed.dir,"interpro")
data.counts.dir<-file.path(data.processed.dir,"counts")
data.reference.dir<-file.path(data.processed.dir,"ref_files")
data.p.gff.dir<-file.path(data.processed.dir,"gff")
data.p.kegg.dir<-file.path(data.processed.dir,"kegg")
data.tpm.dir<-file.path(data.processed.dir,"tpm")
data.tables.dir<-file.path(data.processed.dir,"tables_figs")
```

```r
#Script with all functions to run transcriptome analysis in .Rmd file


# Function combining previous scripts "reference_files_genes.R" and "reference_files.R"
ref.files <- function(ffn, faa) {
  ref_seq_org<-readAAStringSet(file.path(data.gene.dir,ffn))
  #convert to a dataframe both files
  ref_seq_org.1<-data.frame(ref_seq_org)

  ref_org_sequence_genes <-  tibble::rownames_to_column(ref_seq_org.1,"names") %>%
    mutate(locus_tag=str_extract(names,"([A-Z]+_[A-Z]?[A-Z]?[0-9]+)")) %>%
    mutate(names=str_replace(names,locus_tag,"")) %>%
    select(3,1) %>%
    dplyr::rename(gp_name=names)

  ref_seq_org_2<-readAAStringSet(file.path(data.protein.dir,faa))
  #convert to a dataframe both files
  ref_seq_org_2.1<-data.frame(ref_seq_org_2)

  ref_org_sequence <-  tibble::rownames_to_column(ref_seq_org_2.1,"names") %>%
    mutate(locus_tag=str_extract(names,"([A-Z]+_[A-Z]?[A-Z]?[0-9]+)")) %>%
    mutate(names=str_replace(names,locus_tag,"")) %>%
    select(3,2) %>%
    left_join(ref_org_sequence_genes,by="locus_tag") %>%
    select(1,3,2) %>%
    dplyr::rename(prot_seq=ref_seq_org_2) %>%
    mutate(prot_len=nchar(prot_seq))

}


#function replacing the script "reference_files_genes"
#call this function for all fnn files of organisms in your study
ref.files.genes <- function(ffn) {

  ref_seq_org<-readAAStringSet(file.path(data.gene.dir,ffn))
  #convert to a dataframe both files
  ref_seq_org.1<-data.frame(ref_seq_org)

  ref_org_sequence_genes <-  tibble::rownames_to_column(ref_seq_org.1,"names") %>%
    mutate(locus_tag=str_extract(names,"([A-Z]+_[A-Z]?[A-Z]?[0-9]+)")) %>%
    mutate(names=str_replace(names,locus_tag,"")) %>%
    select(3,1) %>%
    dplyr::rename(gp_name=names)


}

#Open the gff files of reference sequences
gff.org <- function(gff) {
  gff_org<-read.delim(file.path(data.raw.gff.dir, gff), header=F, comment.char="#") %>%
    mutate(info=V9) %>%
    separate(V9,c("locus_tag"),sep=";") %>%
    mutate(locus_tag=str_replace(locus_tag,"ID=","")) %>%
    mutate(gene_name=str_extract(info,"Name=[a-z][a-z][a-z][A-Z]_?[0-9]?")) %>%
    mutate(COG=str_extract(info,"db_xref=COG:COG[0-9][0-9]?[0-9]?[0-9]?[0-9]?[0-9]?")) %>%
    mutate(uniprot=str_extract(info,"UniProtKB:[A-Z]?[0-9]?[A-Z]?[0-9]?[A-Z]?[0-9]?[A-Z]?[0-9]?[A-Z]?[0-9]?[A-Z]?[0-9]?")) %>%
    mutate(ec_num=str_extract(info,"eC_number=[0-9].[0-9]?[0-9]?[0-9]?[0-9]?.[0-9]?[0-9]?[0-9]?.[0-9]?[0-9]?[0-9]?[0-9]?")) %>%
    dplyr::rename(id=V1,start=V4,end=V5,strand=V7) %>%
    select(locus_tag,id,start,end,strand,gene_name,COG,uniprot,ec_num) %>%
    filter(end!= is.na(end))

}

inter.org <- function(faa.tsv) {
  inter_org<-read.delim(file.path(data.interpro.dir, faa.tsv),header = FALSE) %>%
    dplyr::select(-V2,-V10,-V11) %>%
    dplyr::rename(locus_tag=V1,prot_len_id=V3,db=V4,db_id=V5,domain_id=V6,start_do=V7,end_do=V8,e_val=V9,IPR_id=V12,dom_name=V13,go_id=V14) #,Metacys=V15)

}

tpm_cal<-function(counts_l,gff_file){

  # Divide the read counts by the length of each gene in kilobases. This gives you reads per kilobase (RPK).
  # part 1

  # part 2
  df.tpm<-counts_l %>%
    left_join(gff_file, by="locus_tag") %>%
    mutate(length = end-start) %>%
    select(1:ncol(counts_l),length) %>%
    mutate(length=length/1000) %>%
    distinct(locus_tag,.keep_all = TRUE)
  df.tpm.2<-df.tpm[,2:ncol(counts_l)]/df.tpm[,"length"]
  df.tpm.2[is.na(df.tpm.2)] <- 0

  # calculate scalling factor Count up all the RPK values in a sample and divide this number by 1,000,000. This is your “per million” scaling factor.
  df.tpm.sum.2<-df.tpm.2 %>%
    summarise_all(funs(sum))
  df.tpm.sum.3<-df.tpm.sum.2/1000000

  # Divide the RPK values by the “per million” scaling factor. This gives you TPM
  df.tpm.3<- cbind(df.tpm.2,df.tpm.sum.3)
  df.tpm.4<-df.tpm.3[,1:(ncol(df.tpm.3)/2)]/df.tpm.3[,((ncol(df.tpm.3)/2)+1):ncol(df.tpm.3)]

  df.tpm.5<-cbind(df.tpm[,1],df.tpm.4)

  colnames(df.tpm.5)[1]<-"locus_tag"

  return(df.tpm.5)
}
```

```r
#this makes a list of all the counts files
list_of_files <- list.files(path = file.path(data.raw.dir, "raw_counts"), full.names = TRUE)

#function to read the counts data. files location are already specified line 2.
read.counts.data <- function(path){

  file.name <- str_split(path, "/") %>% map(., last)
  sample.name <- str_split(file.name, "\\.") %>% map(., dplyr::first) %>% unlist() %>%
    str_remove("tri_")
  df.out <-read.table(path, header = FALSE, skip = 1, sep = "",
                      col.names = c("locus_tag", "counts")) %>%
                                mutate(sample_name = sample.name) %>%
                                mutate(locus_tag=as.character(locus_tag))

  return(df.out)
}

df_counts_raw <- map_dfr(list_of_files, read.counts.data) %>%
  separate(sample_name, into = c("condition", "replicate", "org"), remove = F)




open.counts.data <- function(org_id, orgs_to_exlude, ref_genes) {

  df.counts.org <- df_counts_raw %>%
  filter(org  %in% c(org_id)) %>%
  select(-c(sample_name )) %>%
  unite('sampleAndReplicate', condition:replicate, remove=TRUE) %>%
  spread(sampleAndReplicate, counts) %>%
  filter(!str_detect(locus_tag,orgs_to_exlude)) %>%
  select(-2)

  df.counts.org2<-ref_genes %>%
  left_join(df.counts.org,by="locus_tag") %>%
  select(-2) %>%
  mutate_if(., is.numeric, ~replace(., is.na(.), 0))


}
```
